# *Wwc2* Is a Novel Cell Division Regulator During Preimplantation Mouse Embryo Lineage Formation and Oogenesis

**DOI:** 10.3389/fcell.2020.00857

**Published:** 2020-09-17

**Authors:** Giorgio Virnicchi, Pablo Bora, Lenka Gahurova, Andrej Šušor, Alexander W. Bruce

**Affiliations:** ^1^Laboratory of Early Mammalian Developmental Biology, Department of Molecular Biology and Genetics, Faculty of Science, University of South Bohemia, České Budějovice, Czechia; ^2^Laboratory of Biochemistry and Molecular Biology of Germ Cells, Institute of Animal Physiology and Genetics, Czech Academy of Sciences, Liběchov, Czechia

**Keywords:** oocyte maturation, preimplantation mouse embryo, blastocyst cell number, cell-fate, cell lineage decision, cell division

## Abstract

Formation of the hatching mouse blastocyst marks the end of preimplantation development, whereby previous cell cleavages culminate in the formation of three distinct cell lineages (trophectoderm, primitive endoderm and epiblast). We report that dysregulated expression of *Wwc2*, a genetic paralog of *Kibra/Wwc1* (a known activator of Hippo-signaling, a key pathway during preimplantation development), is specifically associated with cell autonomous deficits in embryo cell number and cell division abnormalities. Division phenotypes are also observed during mouse oocyte meiotic maturation, as *Wwc2* dysregulation blocks progression to the stage of meiosis II metaphase (MII) arrest and is associated with spindle defects and failed Aurora-A kinase (AURKA) activation. Oocyte and embryo cell division defects, each occurring in the absence of centrosomes, are fully reversible by expression of recombinant HA-epitope tagged WWC2, restoring activated oocyte AURKA levels. Additionally, clonal embryonic dysregulation implicates *Wwc2* in maintaining the pluripotent epiblast lineage. Thus, *Wwc2* is a novel regulator of meiotic and early mitotic cell divisions, and mouse blastocyst cell fate.

## Introduction

Following fertilization of metaphase II arrested (MII) mouse oocytes, a series of asynchronous cleavage divisions leads to the formation of blastocyst embryos (at E4.5) that comprise three distinct cell lineages. These include an outer-residing, differentiating and apical-basolaterally polarized epithelium called the trophectoderm (TE; a progenitor of placental tissue), another polarized mono-layer of differentiating inner-cell mass (ICM) cells in contact with the fluid filled cavity called the primitive endoderm (PrE; progenitors of yolk sac membranes) and pluripotent cells that are completely encapsulated within the ICM called the epiblast (EPI; fetal cell progenitors) (see reviews – Frum and Ralston, 2015; Chazaud and Yamanaka, 2016; Rossant, 2016, 2018; Mihajlovic and Bruce, 2017; White et al., 2018). The differential regulation of Hippo-signaling [originally identified in *Drosophila* as a cell proliferation and tissue growth regulating pathway, now implicated in varied developmental/pathological paradigms (Davis and Tapon, 2019)] has been identified as an important mechanism of blastocyst lineage specification. Without listing all involved molecular players [see reviews (Hirate et al., 2015; Chazaud and Yamanaka, 2016; Sasaki, 2017)], polarity dependent Hippo-pathway suppression in outer cells enables formation of activating TEAD4 transcriptional complexes (involving nuclear localisation of specific co-factors, YAP and WWTR1/TAZ, collectively referred to here as YAP) to potentiate TE specific gene expression, whereas activated Hippo-signaling in apolar inner cells inhibits this process (via activating LATS1/2 kinases to prevent YAP nuclear localisation in a phosphorylation dependent manner) (Nishioka et al., 2009). TEAD4-YAP complexes also simultaneously suppress pluripotent gene expression (e.g., *Sox2*) in derived outer-cells (Wicklow et al., 2014; Frum et al., 2018) and prevent precocious *Sox2* expression prior to the 16-cell stage (Frum et al., 2019). However, eventual EPI specification by the late blastocyst stage, actually requires ICM cell YAP redistribution to the nucleus (implying suppression of Hippo-signaling) in an inherently heterogeneous process that causes competitive apoptotic elimination of EPI progenitors of reduced naïve pluripotency (Hashimoto and Sasaki, 2019). Collectively, these data illustrate the important and integral nature of Hippo-signaling in regulating key cell fate events in preimplantation mouse embryo development. We hypothesize they also indicate potential roles for other functionally upstream, uncharacterised and potentially novel factors (related to the core Hippo-pathway machinery) that may be functionally important during early mouse embryogenesis.

The *Drosophila* WW- and C2-domain containing (WWC-domain) gene *Kibra* is a positive regulator of Hippo-signaling, causing phosphorylation of the fly ortholog of mammalian LATS1/2 (warts/Wts) (Baumgartner et al., 2010; Genevet et al., 2010; Yu et al., 2010); a role confirmed in mammalian cell lines (Xiao et al., 2011a). Unlike *Drosophila*, tetrapod genomes typically encode three WWC-domain paralogs, called *KIBRA/WWC1*, *WWC2* and *WWC3*, although the *Mus musculus* genome does not contain an equivalent *Wwc3* gene due to an evolutionarily recent chromosomal deletion. The three paralogous human WWC-domain proteins are highly conserved, cable of homo- and hetero-dimerisation, can all activate Hippo-signaling (causing LATS1/2 and YAP phosphorylation) and result in the Hippo-related *Drosophila* ‘rough-eye’ phenotype, caused by reduced cell proliferation, when over-expressed in the developing fly eye (Wennmann et al., 2014). Despite a comparatively large and pan-model KIBRA-related literature, the roles of WWC2/3 are considerably understudied and restricted to limited prognostic reports consistent of tumor suppressor function in specific cancers [e.g., hepatocellular carcinoma (Zhang et al., 2017) and epithelial-mesenchymal lung cancers (Han et al., 2018)]. There are no reports of any functional roles for WWC-domain containing genes during mammalian preimplantation development.

Mouse MII oocytes arise from the maturation of subpopulations of meiosis I (MI) prophase arrested primary oocytes, stimulated to re-enter meiosis by maternal reproductive hormones [reviewed (Sanders and Jones, 2018)]. Failed bivalent chromosome segregation, resulting in egg and/or zygotic aneuploidy, has usually terminal consequences for embryonic development and aneuploidy attributable to the human female germline is recorded as the leading single cause of spontaneously aborted pregnancy (Hassold and Hunt, 2001; Nagaoka et al., 2012). An extensive literature covering many aspects of the germane segregation of homologous chromosomes during MI exists [see comprehensive reviews (Bennabi et al., 2016; Mihajlovic and Fitzharris, 2018; Mogessie et al., 2018; Namgoong and Kim, 2018; Sanders and Jones, 2018)]. As in all mammals, and unlike most mitotic somatic cells, mouse meiotic spindle formation occurs in the absence of centrioles/centrosomes and is initiated around condensed chromosomes from coalescing microtubule organising centres (MTOCs) that are further stabilized by chromosome derived RAN-GTP gradients (Bennabi et al., 2016; Severson et al., 2016; Gruss, 2018; Mogessie et al., 2018; Namgoong and Kim, 2018). Transition from MTOC initiated spindle formation to centrosomal control in mice only occurs by the mid-blastocysts (E4.0) stage, when centrosomes appear *de novo* (Courtois et al., 2012), and contrasts with other mammalian species in which the fertilizing sperm provides a founder centriole that duplicates and ensures the first mitotic spindle is assembled centrosomally (Sathananthan et al., 1991; Schatten and Sun, 2009). Amongst the known key regulators of meiotic/mitotic spindle dynamics are the conserved Aurora-kinase family (AURKA, AURKB, and AURKC, collectively referred to here as AURKs) that all exhibit germ cell and early embryonic expression [AURKC is not expressed in other somatic cells (Li et al., 2017)]. During meiosis, AURKs have important regulatory roles during MTOC clustering, spindle formation/organization, chromosome condensation and alignment. Moreover, in mitosis, AURKs are also known to regulate correct microtubule-kinetochore attachment, chromosomal cohesion and cytokinesis (reviewed in Nguyen and Schindler, 2017). Specifically, the AURKA protein is essential for oocyte meiotic progression (Saskova et al., 2008) and continually localizes with MTOCs and MI/MII spindle poles, regulating MTOC clustering and initiating microtubule nucleation/dynamics (Swain et al., 2008; Solc et al., 2012; Nguyen and Schindler, 2017; Nguyen et al., 2018); with similar roles in post-fertilization zygotes (Kovarikova et al., 2016).

Here we report that impaired expression of the Hippo-pathway related WWC-domain gene paralog *Wwc2* in mouse embryo blastomeres causes cell autonomous cleavage division phenotypes, yielding blastocysts with fewer overall cells, cell division phenotypes and nuclear abnormalities (not replicated by targeted knockdown of *Kibra* expression). Cell division phenotypes in meiotically maturating mouse oocytes are also caused by RNAi mediated knockdown of *Wwc2* expression and are coincident with spindle defects and blocked activation of AURKA. Such phenotypes are rescuable by the expression of an epitope-tagged recombinant WWC2 protein, that in embryos localizes with mid-body structures and restores appropriate cellular contribution to the ICM, normally lacking and associated with reduced EPI derivation after *Wwc2* dysregulation.

## Materials and Methods

Mouse embryo related experimental procedures were approved and licensed by local ethics committees at the Biology Centre (in České Budějovice) of the Czech Academy of Sciences in accordance with Act No 246/1992 for the protection of animals against cruelty; issued by Ministry of Agriculture of Czechia, 25.09.2014, number CZ02389.

### Embryo and Oocyte Culture and Microinjection

2-cell stage (E1.5) embryo collection from super-ovulated and mated 10 week old F1 hybrid (C57Bl6 x CBA/W) female mice and *in vitro* culture in mineral oil covered drops (∼20 μL/ ∼15 embryos) of commercial KSOM (EmbryoMax^®^ KSOM without Phenol Red; Merck, MR-020P-5F) was conducted as previously described (Mihajlovic et al., 2015). Germinal vesicle (GV) stage oocytes were mechanically recovered into M2 media (containing 100 μM 3-isobutyl-1-methylxanthine/IBMX; cAMP/cGMP phosphodiesterase inhibitor that maintains GV meiosis I arrest) from mature Graafian follicles of dissected ovaries of dams, after pregnant mare serum gonadotrophin (PMSG) hormone stimulation (7.5U intraperitoneal injection of F1 hybrid strain, 48 h prior). Recovered oocytes were placed into pre-warmed (37°C) drops (∼20 μl/ ∼15 oocytes) of commercial CZB media (EmbryoMax^®^ CZB media with Phenol Red; Merck, MR-019-D; +IBMX) overlaid with mineral oil and incubated (37°C in 5% CO_2_) for 2 h prior to any microinjection.

Individual blastomere siRNA/dsRNA or recombinant mRNA microinjections (or combinations thereof) were performed on 2-cell stage embryos (in either one or both blastomeres) according to previously defined protocols (Zernicka-Goetz et al., 1996), using apparatus formerly described (Mihajlovic et al., 2015). Recovered GV staged oocytes were microinjected using a minimally adapted protocol; namely oocytes were microinjected on 37°C heated stage in concaved glass microscope slides containing CZB media (+100 μM IBMX), overlaid with mineral oil. Post-microinjection, oocytes were returned to the incubator for 18 h (to permit microinjected siRNA mediated gene knockdown or mRNA expression; CZB +IBMX media) then transferred to pre-equilibrated CZB media drops (under mineral oil) lacking IBMX, to induce resumption of meiosis I and *in vitro* maturation (IVM; 37°C in 5% CO_2_). Embryos/oocytes were assayed at various developmental points, as dictated by individual experiments. As indicated, rhodamine-conjugated dextran beads (RDBs; 1 μg/μl – ThermoFisher Scientific, D1818) were co-microinjected to confirm successful blastomere/oocyte microinjection. A summary of the origin and concentrations of all microinjected RNA species (including catalog numbers of the commercially available siRNAs) is given in Supplementary Table SM1. Non-microinjected embryos, or oocytes, (2–3 per microinjection experiment, per plate) served as culture sentinels to confirm successful *in vitro* embryo development/oocyte maturation. In cases of AURKA chemical inhibition (to confirm specificity of anti-p-AURKA antisera), recovered GV oocytes were transferred from IMBX containing CZB media to pre-equilibrated CZB drops lacking IBMX but containing the specific AURKA inhibitor, MLN8237 (1μM; Selleckchem, S1133). For each individual experiment three female mice were typically used to recover oocytes/embryos, before being pooled and redistributed between specific experimental or control conditions (thus ensuring oocyte/embryos for a specific experimental or control condition were not derived from one individual female). Moreover, to achieve the n-numbers reported we repeated each experiment at least once.

### dsRNA and Recombinant mRNA Synthesis

Specific long dsRNA molecules targeting coding regions of mouse *Kibra* and *Wwc2* mRNAs (plus negative control GFP) were designed, validated [online E-RNAi resource (Horn and Boutros, 2010)] and synthesized in house. Briefly, gene specific PCR primer pairs, incorporating 5’- T7-derived RNA polymerase promoters and spanning the designed dsRNA complementary sequence, were used to derive *in vitro* transcription (IVT) template, using mouse blastocyst (E3.5) cDNA as a template (or plasmid DNA for GFP control). After agarose gel verification, double stranded DNA templates were used to generate specific dsRNAs in preparatory IVT reactions incorporating DNase I and single-stranded RNase treatments (MEGAscript T7; ThermoFisher Scientific, AMB13345). The integrity of derived *Kibra*-, *Wwc2*- and *GFP*-dsRNAs was confirmed by non-denaturing gel electrophoresis and quantified (Nanodrop). IVT template specific PCR primer sequences are provided in Supplementary Table SM2.

Microinjected mRNA constructs were derived using commercially available IVT reaction kits (for pRN3P/pRN3 vector based constructs; mMACHINE T3, ThermoFisher Scientific - AM1348 & pGEM-T-Easy vector based construct; mMACHINE SP6, ThermoFisher Scientific - AM1340), using restriction enzyme linearized (pRN3P/pRN3 +*SfiI* & pGEMTeasy +*NcoI*) plasmid DNA as template (2 μg) as follows; i. C-terminal GFP-fusion *Gap43* mRNA plasma membrane marker [from pRN3P-C-term-GFP-Gap43 (Morris et al., 2010)], ii. C-terminal RFP-fusion histone H2B mRNA (from pRN3-C-term-RFP-Hist1h2bb, derived in this study) and iii. siRNA-resistant N-terminal HA-tagged *Wwc2* mRNA, *HA-Wwc2* (from pGEM-T-Easy-N-term-HA-siRNAres-Wwc2, derived here). All synthesized mRNAs were subject to post-synthesis 3’ poly-adenylation (Poly(A) Tailing Kit - ThermoFisher Scientific, AM1350) and integrity confirmed by denaturing gel electrophoresis and quantified (Nanodrop).

### Recombinant Plasmid Construct Generation

Three recombinant plasmids (required for IVT) were generated using standard molecular biological protocols. RFP-histone H2B fusion protein (pRN3-C-term-RFP-Hist1h2bb) was derived by in frame cloning of a high-fidelity PCR amplified cDNA (Phusion^®^ DNA polymerase, New England BioLabs, M05305) encoding histone H2B (*Hist1h2bb*, lacking endogenous start and stop codons), flanked by oligonucleotide introduced restriction sites (*NheI*), into the RNA transcription cassette plasmid pRN3-insert-RFP variant (vector encodes required start and stop codons). Thus, generating required H2B-RFP fusion reporter, downstream of vector encoded T3 RNA polymerase promoter (for IVT) and flanked by UTRs from the frog β-globin gene (Lemaire et al., 1995). The IVT plasmid encoding the siRNA-resistant N-terminal HA-tagged *Wwc2* mRNA (pGEM-T-Easy-N-term-HA-siRNAres-Wwc2), *HA-Wwc2*, was created from an annotated full-length Riken mouse *Wwc2* cDNA clone (clone ID: M5C1098O04, Source Bioscience) by derivation of a high fidelity PCR product, with priming oligonucleotide introduced 5’ in frame N-terminal HA-epitope tag, and ‘TA cloned’ (after addition of 3’ adenosine nucleotide overhangs) it into pGEM^®^-T-Easy plasmid vector (Promega, A1360). The derived plasmid (pGEM-T-Easy-N-term-HA-wildtype-Wwc2) was mutagenised (commercial service; EuroFins) to alter nucleotide (but not redundant amino acid codon) sequence of the siRNA recognition motif utilized in this study. The resulting pGEM-T-Easy-N-term-HA-siRNAres-Wwc2 construct was used as template in IVT reaction to generate mRNA resistant to the *Wwc2*-specific siRNA used in this study. All the derived plasmids were sequence verified and the PCR primers (or relevant details) used to generate the required inserts are described in Supplementary Table SM3.

### Q-RT-PCR

Per experimental condition, total RNA was extracted from ∼30 (microinjected) mouse embryos (oocytes) *in vitro* cultured to the desired developmental stage, using the PicoPure RNA isolation kit (Arcturus Biosciences/ThermoFisher Scientific, KIT0204). Eluted RNA (10 μL) was DNase I treated (DNA-*free* kit; ThermoFisher Scientific, AM1906) and used to derive cDNA (30 μL), via oligo-dT (embryos and oocyte) or random hexamer (oocytes only) priming (Superscript-III Reverse Transcriptase; ThermoFisher Scientific, 18080085). 0.5 μl of diluted template cDNA (1:3 dilution in nuclease-free water) per real-time PCR reaction (10 μl – SYBR Green PCR kit, Qiagen, 204143) was used to assay specific transcript abundance (CFX96 Real-Time System, BioRad). *Kibra* and *Wwc2* transcript levels were internally normalized against those for *Tbp* (TATA-binding protein) housekeeping gene and fold changes (± s.e.m.) derived using the ΔΔCt method (Livak and Schmittgen, 2001). A minimum of two biological replicates of at least three technical replicates were employed; specific gene oligo primer sequences (400 nM final reaction concentration) are provided in Supplementary Table SM4.

### Immuno-Fluorescent Staining and Confocal Microscopy Imaging

*In vitro* cultured (microinjected) embryos/oocytes were fixed (at required developmental stages) with 4% para-formaldehyde, immuno-fluorescently (IF) stained and imaged in complete z-series by confocal microscopy (using FV10i confocal microscope, Olympus) as previously described (Mihajlovic et al., 2015); Supplementary Table SM5 summarizes the identity and combinations (plus employed concentrations) of primary and fluorescently conjugated secondary antibodies used. The majority of IF stained embryos/oocytes were counterstained for DNA, using DAPI containing mounting solution (Vectashield plus DAPI, Vector Labs) and as indicated some embryo samples were also counterstained against filamentous-actin, using fluorescently-labeled rhodamine-conjugated phalloidin (ThermoFisher, R415 – described in Mihajlovic and Bruce, 2016).

### Embryo/Oocyte Image Analysis/Cell Counting

The contributions of each individual cell, per fixed embryo (in control or *Kibra/Wwc2* gene RNAi knockdown conditions) to inner (entirely encapsulated) and outer (whereby cells retain cell contactless domains) embryo compartments, blastocyst cell lineages (defined by presence and/or absence of specific and stated lineage marker protein expression – judged by IF), populations defined by the presence of a defining intra-cellular feature (e.g., nuclear morphological abnormalities, apoptosis, polarity marker protein expression or mid-bodies) or presence within or outside a microinjected clone (distinguishable by a fluorescent injection marker signal, i.e., RDBs or expressed mRNA for a recombinant fluorescent fusion protein) was determined by serial inspection of individual confocal micrograph z-sections of each embryo, using commercial Fluoview ver.1.7.a (Olympus), Imaris (Bitplane) and ImageJ software. These calculated contributions were individually tabulated (see Supplementary Tables) and the mean of cells within defined sub-populations, plus standard error of means (mean ± s.e.m.) determined. The statistical significance between relevant experimental and control groups was determined using 2-tailed Student’s t-tests (experiment specific Supplementary Tables provide comprehensive summaries, plus all individual embryo data). Relating to oocytes, a similar approach was used to assay mean frequencies by which maturing oocytes exhibitted intra-cellular phenotypic characteristics (revealed by combined IF staining – i.e., α-TUBULIN and p-AURKA and microinjection of fluorescent reporter – i.e., RFP-histone H2B mRNA) indicative of appropriate or aberrant oocyte maturation at the indicated developmental time-point. These data are individually summarized in relevant Supplementary Tables.

### Western Blotting

Precise numbers of oocytes/embryos per comparable experimental condition (∼15–30) were washed in phosphate buffer saline (PBS; Merck, P5493) containing polyvinyl alcohol (PVA; Merck, 341584), frozen in a residual volume at −80°C, before being lysed by boiling for 5 min in added (10μl) 10x SDS reducing agent/loading buffer (NuPAGE buffer, ThermoFisher Scientific, NP 0004, ThermoFisher Scientific). Lysates were electrophoretically separated on gradient precast 4–12% SDS–PAGE gels (ThermoFisher Scientific, NP0323) and transferred to Immobilon P membranes (Merck group, IVPD00010) using a semi-dry blotting system (Biometra/Analytik Jena) for 25 min at 5 mA/cm^2^. Blotted membranes were blocked in 5% skimmed milk powder dissolved in 0.05% Tween-Tris pH 7.4 buffered saline (TTBS) for 1 h, briefly rinsed in TTBS and then incubated overnight at 4°C in 1% milk/TTBS containing primary antibody. Membranes were washed in three changes of TTBS buffer (20 min each at room temperature) and horse-radish peroxidase conjugated secondary antibody was added to the blot in 1% milk/TTBS, for 1 h (room temperature) and then rinsed in TTBS. Immuno-detected proteins were visualized by chemiluminescence and photographic film exposure (ECL kit; GE Life Sciences, RPN2232), digitally scanned using a GS-800 calibrated densitometer and pixel intensity of non-saturated images recorded with default settings (Bio-Rad Laboratories). Antibody stripped membrane blots were re-probed, for loading controls, in an identical manner. Supplementary Table SM6 details the utilized concentrations of the primary and peroxidase-conjugated secondary antibodies used.

## Results

### WWC-Domain Gene Expression and Knockdown in Preimplantation Mouse Embryos

**FIGURE 1:**
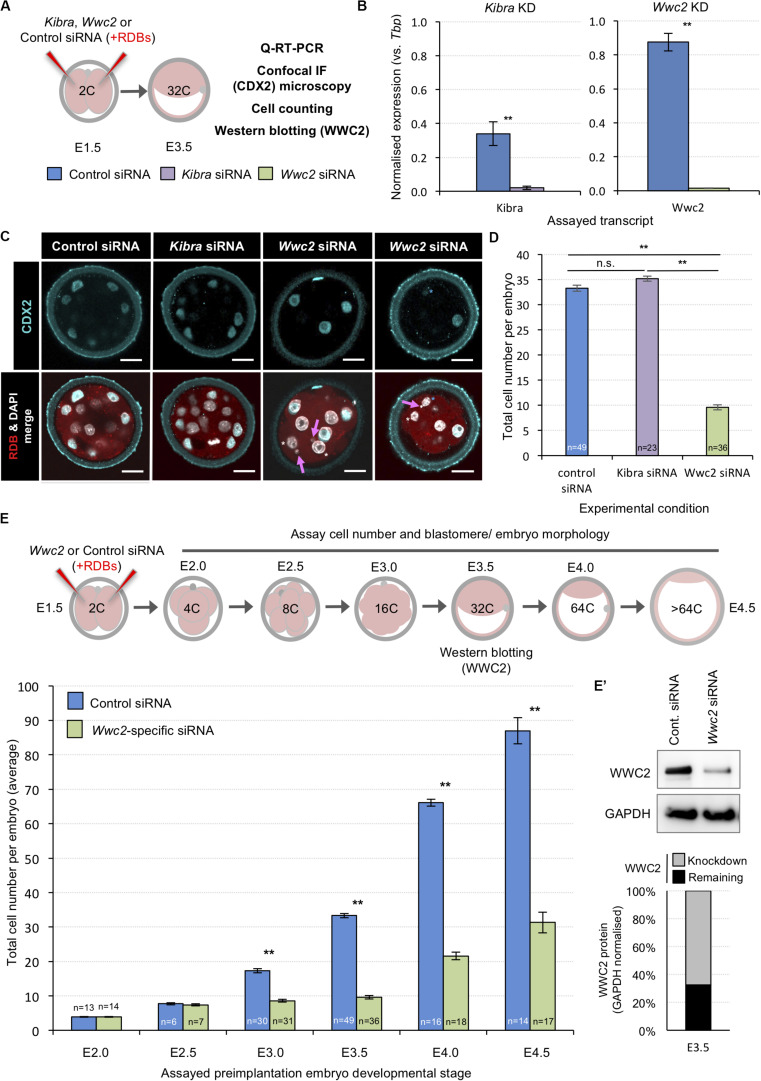
siRNA mediated global *Wwc2* knockdown causes persistent preimplantation embryo cell number deficits from the 8-cell stage. **(A)** Experimental design (relating to panels **B**–**D**, **E’**); non-specific control (color coded blue), *Kibra*-specific (purple) or *Wwc2*-specific (green) siRNA was co-microinjected with rhodamine dextran conjugated beads (RDBs – injection marker) into both blastomeres of 2-cell stage (E1.5) mouse embryos. Microinjected embryos were *in vitro* cultured to the 32-cell stage (E3.5) and either processed for Q-RT-PCR, confocal immuno-fluorescent staining microscopy (assaying CDX2 expression and including a total cell assay count) or western blotting to assay WWC2 protein expression. **(B)** Q-RT-PCR analysis of global siRNA mediated knockdown of endogenous *Kibra* (left) and *Wwc2* (right) mRNA at the 32-cell stage (error bars denote s.e.m. of triplicate measurements, *n* = 3, **p < 0.005). **(C)** Exemplar single *z*-stack micrographs of control, *Kibra*-specific and *Wwc2*-specific siRNA microinjected 2-cell stage embryos cultured to the 32-cell stage and immuno-fluorescently stained for CDX2 expression (CDX2 – cyan, RDBs –red and DNA/DAPI – white; merged images in lower panels). Magenta arrows denote micronuclei specifically observed in the *Wwc2*-siRNA microinjected group and blue and white asterisks outer cells with reduced or absent CDX2 protein expression, respectively; scale bar = 20μm. **(D)** Average total cell number in control, *Kibra*-specific or *Wwc2*-specific siRNA microinjected 2-cell embryos cultured to the 32-cell stage. **(E)** Similar analysis to panel **(D)**, whereby control and *Wwc2*-specific siRNA microinjected 2-cell stage embryos were cultured to the indicated developmental stages and the average total cell number calculated (according to the stated experimental schema – upper panel). In panels **(D,E)** errors represent s.e.m. and 2-tailed student t-test derived statistical significance between control, *Kibra* KD and *Wwc2* KD groups indicated (**p < 0.005); the number of embryos in each group is also indicated. Note, the 32-cell/E3.5 data in both panels is derived from the same analysis. **(E’)** Western blot confirming robust WWC2 protein expression knockdown in 2-cell stage *Wwc2*-specific siRNA microinjected embryos cultured to the 32-cell stage versus the control group. Quantification of the representative western blot (upper panels) is provided in chart form (lower panel) detailing the extent of normalized WWC2 protein knockdown observed. Supplementary Tables ST1–ST6 summarize statistical analyses and individual embryo data. Note, additional data describing frequencies of observable cell morphological/division defects, obtained from this same dataset are also presented in Figure 2 and Supplementary Figure S4.

Within the context of Hippo-signaling during early mouse development (Sasaki, 2017), we investigated the potential role of WWC-domain containing genes, *Kibra* and *Wwc2* (Wennmann et al., 2014), during blastocyst formation using specific siRNA mediated gene knockdown (KD). Preimplantation mouse embryos were recovered at the 2-cell (E1.5) stage and microinjected in both blastomeres with either non-specific control, *Kibra*- or *Wwc2*-specific siRNA constructs (together with rhodamine conjugated dextran beads/RDBs, to act as a fluorescent marker of successful microinjection) and cultured to the 32-cell (E3.5) stage (summarized in Figure 1A; Supplementary Figure S1 details the specific siRNA designs). Q-RT-PCR analysis confirmed the expression of both *Kibra* and *Wwc2* mRNA in control embryos, with *Wwc2* transcripts being over twice as abundant as those for *Kibra.* Moreover, that such expression was significantly and robustly reduced by the specific *Kibra/Wwc2* siRNA treatments (Figure 1B). Immuno-fluorescent (IF) confocal microscopy analysis of similarly treated/microinjected embryos did not reveal any ectopic CDX2 expression within the ICM of either *Kibra* or *Wwc2* KD embryos, that could be expected if activation of the Hippo-signaling pathway was blocked or impaired (Figure 1C); suggesting neither gene is required for activation of Hippo-signaling in inner cells to supress *Cdx2* expression. Indeed, CDX2 expression was observed in the outer cells of *Wwc2* KD embryos but was sometimes observed to be significantly less robust or absent when compared to the levels observed in either the control or *Kibra*-specific siRNA microinjected groups, imaged under the same conditions (Figure 1C – see blastomeres marked with asterisks, often also associated with presence of nuclear abnormalities such as micronuclei, denoted by magenta arrows – expanded below). Strikingly, an assay of the average total cell number of embryos in each microinjection group revealed *Wwc2* KD was associated with a profound and statistically significant reduction in total cell number versus the control embryo group. This was not observed after *Kibra* KD (Figure 1D). To probe this phenotype further we repeated both the control and *Wwc2*-specific 2-cell stage siRNA microinjections and assayed the average total cell number in embryos cultured to all other subsequent preimplantation cleavage stages (Figure 1E). We found that both control and *Wwc2* KD embryos developed in step to the 8-cell (E2.5) stage. However, whilst control embryos continued to transit through the 16-cell (E3.0) to late blastocyst (> 64-cell/E4.5) stages without delay, *Wwc2* KD embryos did not significantly increase in cell number again until after the 32-cell stage (at which time WWC2 protein KD was quantified to be 67% efficient – Figure 1E’). Even after this stagnant period of total cell number growth the observed rate of cell number increase, as observed in *Wwc2* KD embryos from the mid-blastocyst (E4.0) stage onwards, still lagged behind that observed in control siRNA microinjected embryos (Figure 1E). We next assayed if the observed *Wwc2* siRNA phenotypes were cell autonomous by creating RDB marked *Wwc2*-specific siRNA KD clones that comprised 50% of the embryo (via microinjecting one blastomere at the 2-cell stage) and assaying their contribution to total embryo cell number at all preimplantation cleavage stages. As shown in Supplementary Figure S2, at all post-8-cell stages we observed a statistically significant *Wwc2* siRNA mediated deficit in cell number that was only manifest within the progeny of the RDB marked microinjected clone (as compared to both the number of cells within the non-microinjected sister clones of the same embryos and those of the equivalent microinjected clones in control siRNA microinjected embryos – Supplementary Figure S2). We did not observe significant differences between control and *Wwc2* KD groups in the number of cells within the non-microinjected clone (with the exception of the 32-cell stage where there was an average of 2.3 fewer cells in the *Wwc2* KD group). Indeed, such cell autonomous cell number deficits were also independently observed in embryos with confirmed *Wwc2* KD mediated by microinjection of alternative long double stranded RNA (dsRNA) constructs but consistently absent after dsRNA mediated *Kibra* KD (Supplementary Figure S3; construct details given in Supplementary Figure S1). Collectively, these data support a novel and cell autonomous role for the Hippo-signaling related paralog *Wwc2* in ensuring appropriate cell number during preimplantation mouse embryo development.

### Reduced Embryo Cell Numbers Caused by *Wwc2* KD Are Associated With Defective Cell Division

**FIGURE 2:**
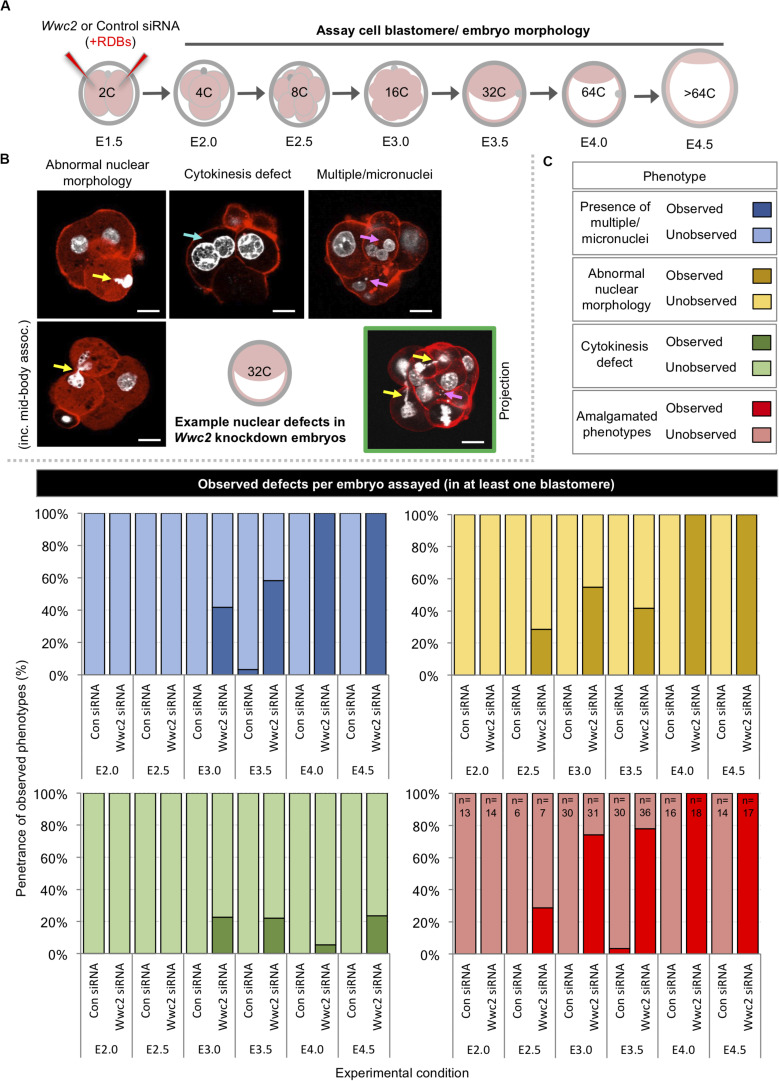
Global *Wwc2* knockdown mediated total embryo cell number deficits are associated with defective cell division morphologies. **(A)** Schematic of experimental design; 2-cell stage embryos microinjected with control or *Wwc2*-specific siRNA were cultured to indicated developmental stages, fixed and assayed for total cell number or the incidence of abnormal cell morphology indicative of defective cell division (determined by DAPI and rhodamine conjugated phalloidin staining – note, data derived from same experiments as those described in Figure 1). **(B)** Exemplar confocal micrograph z-sections of *Wwc2*-specific siRNA microinjected embryos at the 32-cell stage, illustrating three distinct phenotypic morphological defect categories observed; (i) abnormal nuclear morphology (including chromatin mid-body association, highlighted by yellow arrows – left), (ii) cytokinesis defects defined by two nuclei per cell (highlighted by blue arrow – center), (iii) presence of multiple nuclei and/or micronuclei per cell (> 3 highlighted by magenta arrows - right) and (iv) composite of categorized defects highlighted by color coded arrows in a single embryo confocal z-section projection (green boarder). DAPI (white) and cortical F-actin (red); scale bar = 20 μm. **(C)** Frequencies of each observed category of phenotypic/morphological defect (i.e., micronuclei; blue, abnormal nuclei; yellow and failed cytokinesis; green) or a combination of at least two or more (red) in control siRNA and *Wwc2*-specific siRNA microinjected embryos, at indicated developmental stages (numbers of embryos in each group highlighted, see amalgamated data – red); data demonstrate defect incidence per embryo (in at least one constituent blastomere). Supplementary Tables ST1–ST6 summarize statistical analysis and individual embryo data in each experimental group. Supplementary Figure S4 also details the same data represented as defect incidence per blastomere/cell assayed in all embryos of each group.

As stated above, during our analysis of *Wwc2* KD induced total cell number deficit phenotypes we observed morphological nuclear abnormalities, not evident in the control (or *Kibra* KD) groups. Illustrative examples from embryos assayed at the 32-cell equivalent stage are shown in Figure 2B (although embryos were assayed at all cleavage stages – Figure 2A). Such defects were categorized as i. ‘abnormal nuclear morphology’ (including that typical of ‘association with the mid-body’), ii. associated with ‘cytokinesis defects’ (typified by bi-nucleated cells) or iii. coincident with ‘multiple or micronuclei’. We calculated the frequencies by which each or a composite of such abnormalities were observed at each assayed cleavage stage, as defined per embryo (in at least one blastomere – Figure 2C) or per individual assayed cell (Supplementary Figure S4). Apart from a single incidence of an early blastocyst stage cell exhibiting a single micronucleus, no abnormalities were observed in any control siRNA microinjected embryo, at any developmental stage. Conversely, at the 8-cell stage abnormally shaped nuclei were present in over a quarter of *Wwc2* global KD embryos and in all assayed embryos by the equivalent late blastocyst stage. A similar trend assaying multiple/micronuclei was observed from the 16-cell stage. Although less prevalent, cytokinetic defects also affected nearly a quarter of *Wwc2* KD embryos at the late blastocyst stage. Indeed, all *Wwc2* global KD embryos exhibited one or more defect, in at least one cell, by the mid-blastocyst stage, with the collective defects (excluding cytokinesis/bi-nucleation) first arising in just over a quarter of 8-cell stage embryos (Figure 2C). An assay of the same phenotypes at the level of each individually assayed blastomere (Supplementary Figure S4) showed that just over a quarter of cells were affected by the late blastocyst stage, although as described above, such embryos comprised a much smaller average of overall cells versus control siRNA groups (i.e., 31.3 ± 3.0 against 87.0 ± 3.8 - Figure 1E). Thus, cleavage stage *Wwc2* KD is associated with cell autonomous division defects that contribute to embryos with progressively fewer constituent blastomeres from the 8-cell stage and invokes a role for *Wwc2* in regulating appropriate cell division in the early mouse embryo.

### *Wwc2* mRNA Depletion in Mouse (GV) Oocytes Impairs Meiotic Maturation

**FIGURE 3:**
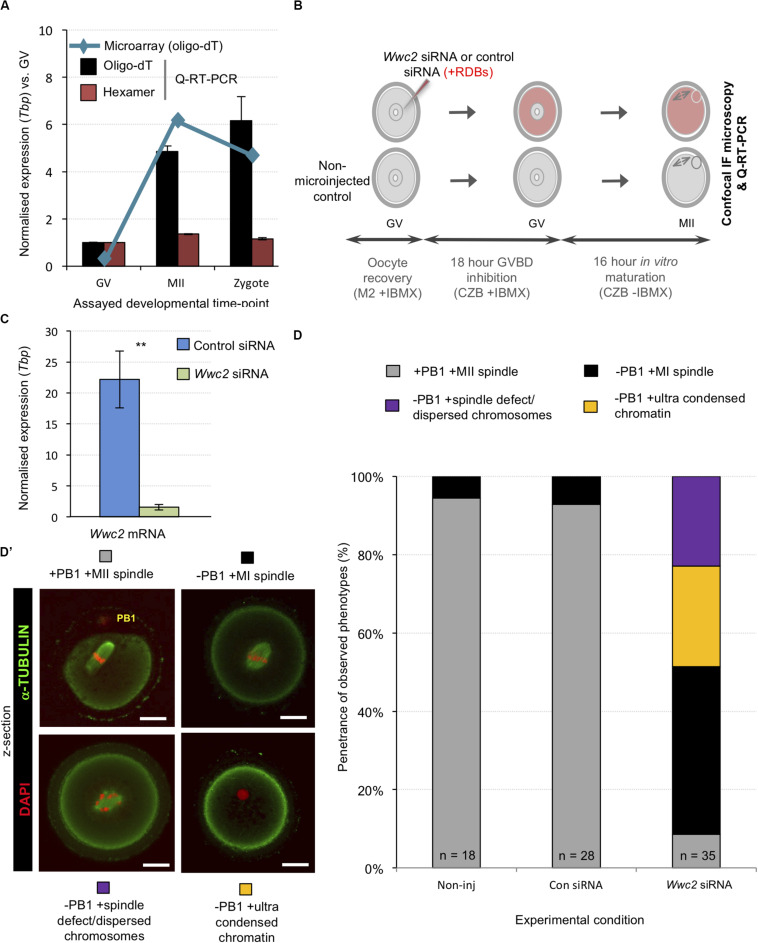
*Wwc2* mRNA is required for mouse oocyte meiotic maturation. **(A)** Published microarray (Wang et al., 2004, blue line) and generated Q-RT-PCR (bar charts) normalized *Wwc2* mRNA expression in germinal vesicle and metaphase II arrested oocytes (GV and MII) and fertilized zygotes (*Tbp* normalized expression, relative to GV stage). Note, microarray data derived from oligo-dT primed reverse transcription and Q-RT-PCR from both oligo-dT (black bars) and random hexamer priming (red bars); error bars represent s.e.m. and *n* = 3. **(B)** Experimental schema of *Wwc2* knockdown in GV oocytes by microinjected *Wwc2* siRNA (plus control siRNA and non-microinjected controls), 18 h incubation in IMBX containing media (preventing GVBD), 16 h *in vitro* oocyte maturation (IVM – minus IMBX) and confocal microscopy or Q-RT-PCR analysis; co-microinjected RDBs used as injection control marker. **(C)** Q-RT-PCR data of normalized (against *Tbp*) *Wwc2* mRNA levels in control and *Wwc2* siRNA microinjected GV oocytes after IVM to the MII equivalent stage (error bars represent s.e.m., *n* = 3 and **p < 0.005 in 2-tailed student *t*-test). **(D)** Charts detailing successful IVM frequencies of control non-microinjected (Non-inj), microinjected control siRNA (Con siRNA) and microinjected *Wwc2* siRNA GV oocytes, to the MII stage or indicated phenotypic stages. **(D’)** Examples of successfully matured MII oocytes (note, extruded first polar body/PB1) and the observed and quantified categorized phenotypes shown as confocal z-section micrographs (α-Tubulin in green and DAPI DNA pseudo-colored red; scale bar = 20 μm). Supplementary Figure S5 details the complete confocal *z*-series of the illustrative oocyte examples shown in panel **(D’)**. Supplementary Table ST14 summarize statistical analysis and individual oocyte data used to generate the figure.

We next addressed whether similar *Wwc2* KD in germinal vesicle (GV) oocytes could cause subsequent meiotic division defects, particularly as both embryo cleavage divisions and meiotic oocyte maturation occur in the absence of centrosomes in mice (Courtois et al., 2012; Coelho et al., 2013; Bennabi et al., 2016; Severson et al., 2016; Gruss, 2018; Mogessie et al., 2018; Namgoong and Kim, 2018). We first confirmed by Q-RT-PCR abundant *Wwc2* transcript expression in GV and MII oocytes (plus zygotes). Exploiting alternative, oligo-dT versus random hexamer, cDNA synthesis priming strategies we uncovered evidence of cytoplasmic *Wwc2* mRNA poly-adenylation, usually associated with increased translational efficiency of functionally important proteins during meiotic maturation (Salles et al., 1992) – Figure 3A. Consultation of our previously published assay of polysome associated transcripts during mid-meiotic maturation supported this interpretation, reporting ∼40% polysome association of detectable *Wwc2* transcripts (Koncicka et al., 2018; Masek et al., 2020). We then confirmed by Q-RT-PCR that our siRNA construct could elicit robust *Wwc2* transcript knockdown in microinjected GV oocytes blocked from re-entering meiosis I (using the cAMP/cGMP phosphodiesterase inhibitor, 3-isobutyl-1-methylxanthine/IMBX) but then permitted to *in vitro* mature (IVM – via IBMX removal) to the MII equivalent stage (Figures 3B,C). Similarly treated oocytes were then fixed and IF stained for α-Tubulin (plus DAPI DNA stain) and assayed by confocal microscopy for potential meiotic maturation/division phenotypes. In the two control conditions (non-microinjected or control siRNA microinjected oocytes) > 90% of GV oocytes matured to the MII arrested stage, typified by extruded polar bodies (PB1) and metaphase II arrested spindles. However, in the *Wwc2* KD group, this successful IVM rate was reduced to 8.6%. The remaining oocytes presented with various MI arrested phenotypes (all lacking PB1) categorized by, i. presence of MI metaphase spindles (-PB1 +MI spindle; 42.9%), ii. spindle-like structures with mis-aligned or dispersed chromosomes (-PB1 +spindle defect; 22.9%) or iii. non-nuclear membrane enveloped chromatin devoid of associated α-Tubulin (-PB1+ultra-condensed chromatin; 25.7%). Such phenotypes collectively indicate profoundly defective oocyte meiotic maturation and impaired homologous chromosome segregation caused by *Wwc2* KD (Figures 3D,D’ and Supplementary Figure S5). Although assayed oocytes were subject to IVM for 18 h, we wanted to exclude the possibility the results were indicative of delayed rather than blocked meiotic progression. Consistently, experimental repetition using an extended IVM period of 24 h did not reveal any increased frequency of MII arrested oocyte generation. Indeed, *Wwc2* KD oocytes either remained blocked in MI or exhibited increased incidence of dispersed chromosomes and spindle defects, potentially connected to attempted chromosome segregation without attendant cytokinesis (Supplementary Figure S6).

**FIGURE 4:**
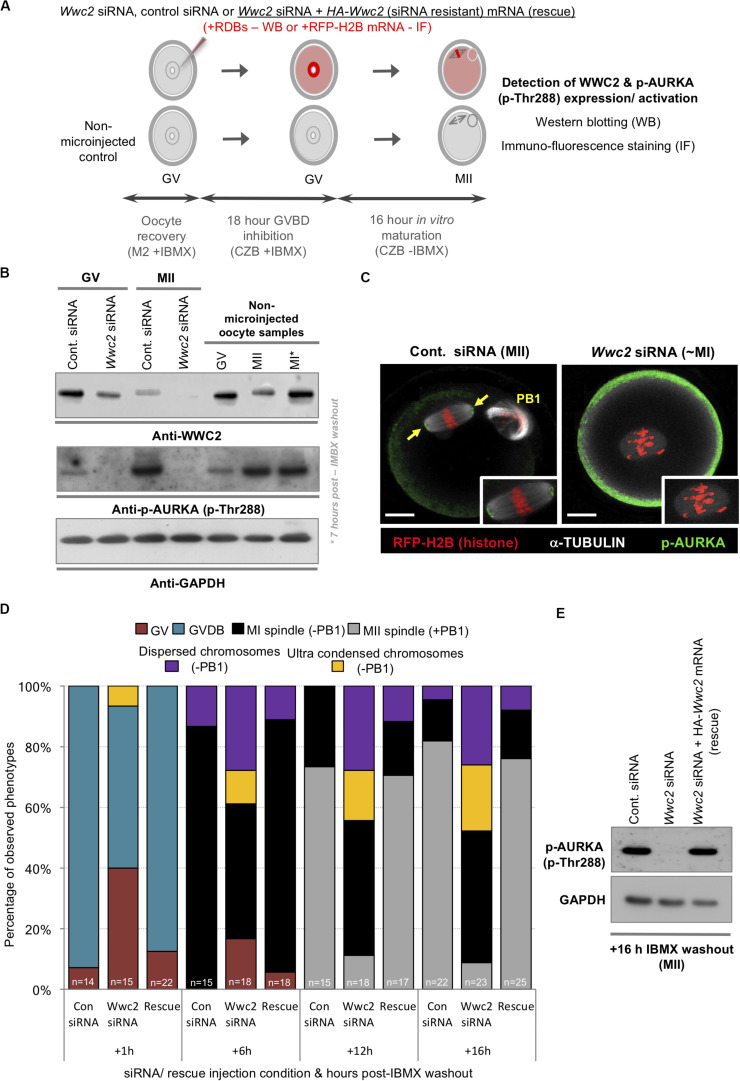
*Wwc2* knockdown induced oocyte IVM phenotypes are associated with failed Aurora-A (AURKA) phosphorylation/activation (rescuable by co-microinjection of siRNA resistant *HA-Wwc2* mRNA). **(A)** Experimental schema of GV oocyte microinjection conditions; i.e., *Wwc2* siRNA or control siRNA alone or *Wwc2* siRNA + *HA-Wwc2* (siRNA resistant) mRNA (i.e., ‘rescue’) were co-microinjected with RDBs (for western blot analysis) or RFP-H2B mRNA (fluorescent marker in IF staining). Microinjected (plus non-microinjected control) oocytes incubated in IMBX containing media (18 h – to prevent GVBD), subject to IVM (max. 16 h – media minus IMBX) and processed for western blotting and IF at designated oocyte maturation time-points (assaying WWC2 and phospho-Aurora/p-AURKA levels) or assayed for *Wwc2* KD induced developmental progression phenotypes. **(B)** Western blots of WWC2 and activated p-AURKA protein levels (plus GAPDH housekeeping control), at indicated stages of oocyte maturation (note, GV; +0 h relative to IMBX washout, metaphase of meiosis I/MI; +7 h and MII; +16 h), in either control or *Wwc2* siRNA microinjected, or non-microinjected conditions. **(C)** Exemplar single confocal z-section IF micrographs of control (left) or *Wwc2* siRNA (right) IVM cultured microinjected oocytes at the MII equivalent stage (16 h post-IBMX) stained for p-AURKA (green) and α-TUBULIN (white) and labeled with RFP-H2B chromatin reporter (red); insets show zoomed region of meiotic spindle. Note, *Wwc2* siRNA microinjection group example image is over-saturated compared to control (to illustrate lack of spindle associated pAURKA – cortical signal is auto-fluorescence); PB1 (control siRNA) denotes extruded first polar body and the scale bars = 20 μm. **(D)** Charts detailing IVM rates of control siRNA, *Wwc2* siRNA and *Wwc2* siRNA + *HA-Wwc2* mRNA (rescue) microinjected oocytes at staged time-points post IBMX removal; as described by percentage of oocytes at any of stated IVM stages or categorized developmental phenotypes (typically associated with *Wwc2* KD – see Figure 3); number of oocytes in each experimental condition at each assayed IVM stage is indicated. **(E)** Western blot of activated p-AURKA levels (plus GAPDH housekeeping control) at MII equivalent stage (16 h post-IVM), in control siRNA, *Wwc2* siRNA or *Wwc2* siRNA + *HA-Wwc2* mRNA microinjected (rescue) oocytes. Supplementary Tables ST16–ST19 summarize statistical analysis and individual oocyte data used to generate the figure.

Despite consistent *Wwc2*-specific RNAi cell division phenotypes uncovered with distinct siRNA/dsRNA constructs in preimplantation mouse embryos and oocytes (plus optimized reagent design to avoid potential off-target effects or cross reactivity with paralogous *Kibra* mRNA – Supplementary Figures S1, S3) we sought further verification of the identified *Wwc2* specific oocyte role. Accordingly, we generated a recombinant and N-terminally HA-epitope tagged *Wwc2* mRNA construct (*HA-Wwc2*), specifically mutated within the siRNA complementary sequence (yet preserving, via redundancy in the genetic code, the sequence of amino acid codons; Supplementary Figure S8) that could remain available for translation in *Wwc2* siRNA microinjected oocytes. Repeating the described IVM experiments (but now assaying at multiple intervening time-points), we found that co-microinjection of *HA-Wwc2* mRNA and *Wwc2* siRNA caused a robust rescue of the maturation phenotypes previously observed by *Wwc2* siRNA microinjection alone (i.e., MII arrested oocytes +PB1 at 16 h post-IBMX; control siRNA – 81.8%, *Wwc2* siRNA - 8.7% & *HA-Wwc2* mRNA plus *Wwc2* siRNA – 76.0%). Moreover, the progression of the observed rescue through recognizable oocyte maturation stages was in-step with that of control siRNA microinjected oocytes (as measured at 1, 6, 12 and 16 h post-IBMX removal; Figure 4D). We also found that *HA-Wwc2* mRNA co-microinjection was associated with reappearance of detectable p-AURKA protein expression (Figure 4E) and sub-cellular localisation with forming or matured MI/MII stage meiotic spindles (Supplementary Figure S9). Collectively, these data confirm a novel *Wwc2* role in regulating appropriate chromosome segregation and cell division during mouse oocyte maturation (associated with activation of the key spindle-associated regulatory kinase AURKA); displaying functional conservation with that observed in similarly acentrosomal mitotic cleavage divisions of preimplantation stage embryo blastomeres.

In summary, these data confirm *Wwc2* KD clone specific and autonomous reductions in overall embryo cell number that more robustly affect formation of ICM versus TE. Moreover, ICM lineage contribution of *Wwc2* KD clones is also biased against the pluripotent EPI and favors PrE differentiation, potentially via a mechanism involving selective apoptosis of specified EPI. Thus, uncovering a blastocyst cell fate role of *Wwc2* that is additional to that related to cell division.

### *Wwc2* KD Oocyte Phenotypes Are Associated With Failed Aurora Kinase-A (AURKA) Activation

Phosphorylation dependent activation of Aurora kinase-A (p-AURKA: p-Thr288) is required to initiate acentrosomal and MTOC mediated spindle assembly in mouse oocytes and *Aurka* KD phenotypes closely resemble the *Wwc2* KD defects we describe (Bury et al., 2017). We hypothesized the observed *Wwc2* specific meiotic maturation phenotypes may be associated with impaired p-AURKA activation. Western blot analyses of staged maturing oocytes (experimental schema – Figure 4A) confirmed characteristically low p-AURKA levels in GV oocytes, that markedly increased in the presence of the MI spindle and were maintained when the MII spindle was formed (Figure 4B; Saskova et al., 2008; Nguyen and Schindler, 2017); a trend replicated in control siRNA microinjected GV (prior to IBMX washout) and resulting MII oocytes (the anti-p-AURKA antibody specificity was independently confirmed; Supplementary Figure S7). However, p-AURKA was not detectable at either the equivalent GV or MII stages in *Wwc2* siRNA microinjected oocytes and correlated with reduced and near undetectable WWC2 protein expression, respectively. Interestingly, at both the MI or MII equivalent stages, at which WWC2 protein could be detected in control conditions, the mobility of the specific band was retarded compared to that present at the GV stage indicative of potential post-translational modification (Figure 4B). Additionally, p-AURKA immuno-reactivity (assayed by confocal microscopy based IF) that would ordinarily localize to meiotic spindle poles, was also lacking in *Wwc2* KD oocytes that presented with spindle-like structures. This was in stark contrast to that observed on MII arrested spindles of control oocytes (Figure 4C – note the p-AURKA channel of the example *Wwc2* KD oocyte is over-exposed to illustrate the lack of spindle associated immuno-reactivity but artificially accentuates non-specific cortical auto-fluorescence also seen in control oocytes). These results confirm failed p-AURKA activation during oocyte maturation, under conditions of experimentally induced *Wwc2* KD, that is associated with profoundly defective meiotic cell division.

### *Wwc2* Knockdown Affects Blastocyst Cell Fate

**FIGURE 5:**
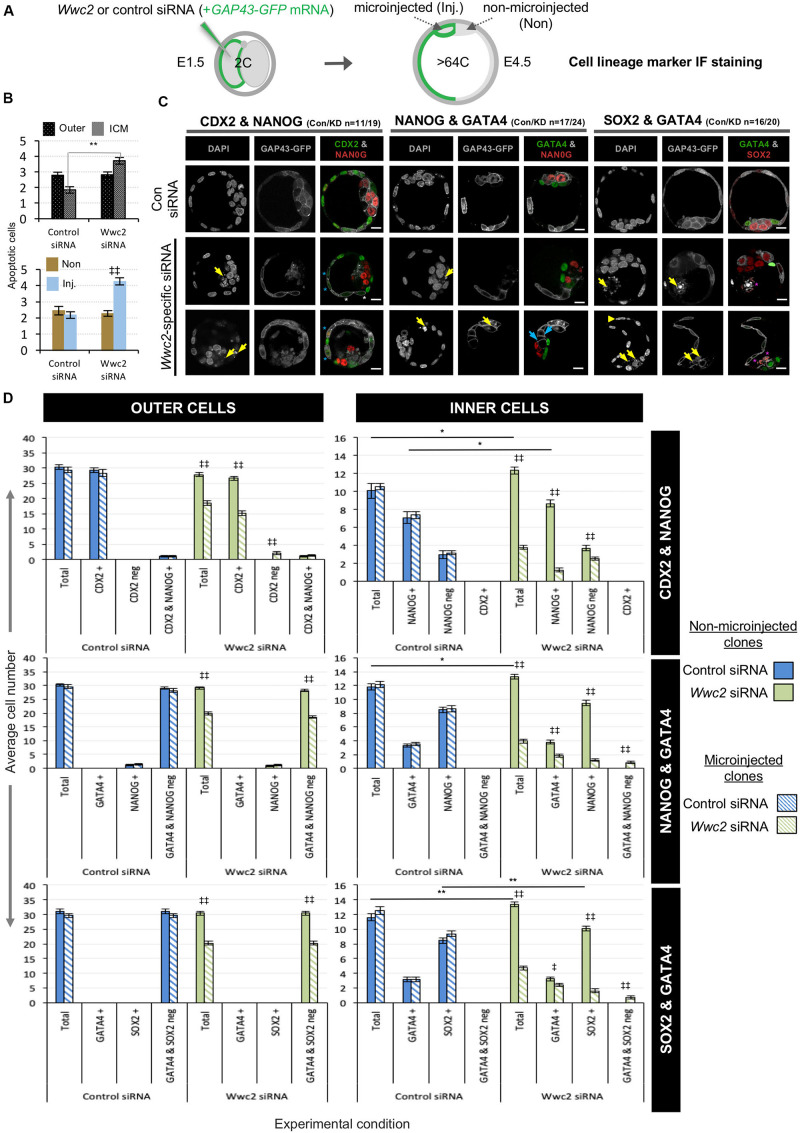
Blastocyst cell lineage formation after clonal *Wwc2* gene knockdown. **(A)** Experimental strategy for *Wwc2* expression knockdown in marked clones (representing 50% of the embryo) by co-microinjection (in one blastomere of 2-cell stage embryos) of *Wwc2* siRNA and GAP43-GFP mRNA (stable plasma membrane fluorescent injection clone marker/lineage marker – microinjected/Inj.) and an IF based assay of clonal contribution (Inj. versus non-microinjected/Non) to blastocyst protein lineage marker expressing cells (TE; CDX2, PrE; GATA4, EPI; NANOG or SOX2; assayed in combination – see below) in outer and inner-cell embryo populations, at the late blastocyst/E4.5 stage (versus control siRNA microinjections). **(B)** Average number of apoptotic cells in control and *Wwc2* siRNA microinjected embryo groups (averaged across all IF regimes – see below) in outer and inner cell populations and clones (Inj. versus Non); errors represent s.e.m. and 2-tailed student *t*-test derived statistical significance between control and *Wwc2* KD groups (asterisks) or clones (Inj. versus Non) within a group (double crosses) highlighted with statistical confidence intervals of *p* < 0.05 and *p* < 0.005 (denoted by one or two significance markers, respectively). **(C)** Exemplar confocal z-section micrographs of IF stained blastocysts (individual greyscale channels plus merged images, in which assayed combinations of cell lineage markers are respectively colored green and red; CDX2 & NANOG, GATA4 & NANOG and GATA4 and SOX2) from control and *Wwc2* siRNA microinjected groups. Within the *Wwc2* siRNA microinjected clone, yellow arrows denote apoptotic cell remnants, blue arrows interphase ICM cells that neither express GATA4 or NANOG, the yellow arrow head a bi-nucleated cell whereas blue or white asterisks highlight outer-cells with basal or undetectable CDX2 expression, respectively and magenta asterisks denote apoptotic ICM cells that IF stain for SOX2. The number of assayed embryos in each control (Con) and *Wwc2* siRNA (KD) microinjection group is provided; scale bar = 20μm. **(D)** The average number of cells contributing to populations expressing, co-expressing or not expressing the stated combinations of late blastocyst lineage marker proteins, their designated clonal origin (microinjected versus non-microinjected) and relative distribution between outer and ICM compartments in both control and *Wwc2* siRNA microinjected embryos; errors represent s.e.m. and 2-tailed student *t*-test derived statistical significance between the control and *Wwc2* knockdown groups (asterisks – note, for simplicity only potential regulative differences between the non-microinjected clones are shown; see Supplementary Tables for full statistical summary) or clones (microinjected versus non-microinjected) within a group (double crosses) are highlighted with statistical confidence intervals of *p* < 0.05 and *p* < 0.005 (denoted by one or two significance markers, respectively). Supplementary Tables ST20–ST22 summarize the statistical analysis and individual embryo data in each experimental group. Note a similar analysis of CDX2 expression at the early-blastocyst/E3.5 stage is provided in Supplementary Figure S10.

Preimplantation mouse embryo cell number deficits and defective division phenotypes were first evident after *Wwc2* KD around the 8- to 16-cell stage (Figures 1, 2 and Supplementary Figure S2). The transition from the 8- to 16-cell stage represents the first developmental point that blastomeres are overtly distinct in terms of their intra-cellular apical-basolateral polarity and relative spatial positioning, with consequences for ultimate blastocyst cell fate [i.e., there are polarized outer-cells that can give rise to both TE and further inner-cells, plus apolar inner ICM progenitors (Johnson and Ziomek, 1981)]. We therefore decided to assay the contribution of stably marked *Wwc2* KD clones to each of the three late blastocyst (E4.5) stage lineages, to ascertain if loss of *Wwc2* function would influence ultimate cell fate. Accordingly, control or *Wwc2*-specific siRNAs were co-microinjected with recombinant GAP43-GFP encoding mRNA (a fluorescent injection and plasma membrane marker) into one blastomere at the 2-cell stage (creating the stably marked clones) and the embryos permitted to develop to the late blastocyst stage before being fixed and IF stained for specific blastocyst cell lineage marker protein expression (Figure 5A). The incidence of apoptosis (Figure 5B) and contribution of the marked control or *Wwc2* KD clones and non-microinjected clones to each blastocyst lineage was then calculated and compared (Figures 5C,D); pairwise IF staining schemes assaying TE and EPI (CDX2 & NANOG) or PrE and EPI markers (GATA4 & NANOG or GATA4 & SOX2) were employed (Figure 5C). As in our previous experiments (Figures 1, 2 and Supplementary Figure S2) *Wwc2* KD clone specific cell number (plus total blastocyst cell number) deficits, nuclear morphology and cell division phenotypes were again observed. Notably, the incidence of fragmented nuclei, consistent with apoptosis (assayed across all the IF staining schemes) was significantly higher within the *Wwc2* siRNA microinjected clones of the ICM versus equivalent control siRNA clones (Figure 5B). Consistently, the overall and significantly reduced contributions of *Wwc2* KD cell clones were also more pronounced for ICM versus outer-cell populations (Figure 5D). Collectively, these data indicate enhanced apoptotic elimination of *Wwc2* KD cells from the ICM, compared to outer-cells. Focussing on the CDX2 and NANOG stained embryo groups, we did not observe any ectopic CDX2 expression within *Wwc2* KD cells resident in the ICM. However, we did again note generally reduced CDX2 protein expression and an unusual and small sub-population of CDX2 negative outer-cells within the *Wwc2* KD clone, when compared to non-microinjected clones from the same embryo or either clone of control siRNA microinjected groups (Figures 5C,D). The significance of such reduced/absent outer-cell expression is unclear but it was also, together with a lack of ectopic ICM CDX2 expression, specifically observed in *Wwc2* KD clones at the 32-cell (E3.5) stage (Supplementary Figure S10). These data suggest that outer-cell Hippo-pathway suppression (specifying TE) and inner-cell activation (preventing TE differentiation and needed to promote pluripotency) are predominately intact within *Wwc2* KD clones, although outer-cell maintenance of TE differentiation is not optimal (and inner cell viability/contribution is severely impaired). Indeed, a lack of overt outer-cell apical polarity defects (assaying PARD6B in IF, at the 16-cell stage) or ectopic nuclear exclusion of YAP (a correlate of activated Hippo-signaling (Nishioka et al., 2009), assayed at the 32-cell stage) within *Wwc2* KD outer blastomeres support this conclusion (Supplementary Figure S11). Although interestingly, YAP nuclear exclusion in inner- *Wwc2* KD cells at the 32-cell stage was consistently less robust than in control siRNA microinjected inner-cells; indicating potential impairment of inner-cell Hippo-signaling activation (Supplementary Figure S11 – although the observed nuclear YAP was not equivalent to outer residing blastomeres). Interestingly, the significantly reduced contribution of *Wwc2* KD cell clones to the ICM did not segregate, as per marked control siRNA clones, between NANOG positive (indicative of EPI) and NANOG negative (potentially PrE) populations. Rather, the contribution was biased towards NANOG negative cells, representative of potential PrE (albeit the overall ICM allocation was severely reduced compared to both non-microinjected sister clones or either clone in control siRNA embryos). Indeed, the overall reduction in this potential PrE population (i.e., NANOG negative ICM cells) that was associated with clonal *Wwc2* KD was substantially smaller than that observed for the corresponding NANOG positive EPI (Figure 5D); further suggesting inner *Wwc2* KD clones are impaired in their sustained contribution to the EPI but are more able to differentiate into PrE. Data from directly assaying the two ICM lineages together (i.e., GATA4 & NANOG or SOX2; Figures 5C,D) also demonstrated characteristically low numbers of inner *Wwc2* KD clones, similarly segregated in favor of the PrE over EPI (although compensatory increases in EPI contribution from non-microinjected clones were noted). Numerous examples of SOX2 positive fragmented inner-cell nuclei within the *Wwc2* KD clone were also noted (Figure 5C); indicating the observed increased incidence of apoptosis (Figure 5B) was centered on specified EPI (although the reliable quantification of SOX2 positive apoptotic nuclei was not possible). Interestingly, we noted a small population of marked ICM *Wwc2* KD clones expressing neither PrE or EPI markers (4.6% of all ICM cells and 18.7% of the clone - not present in controls), potentially indicative of further ICM cell fate defects.

**FIGURE 6:**
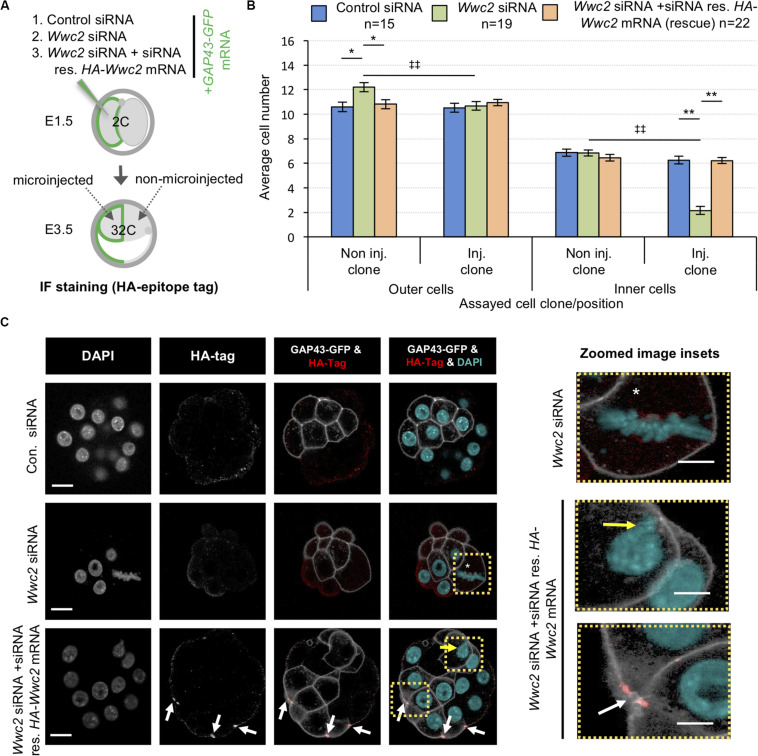
Expression of siRNA resistant *HA-Wwc2* mRNA rescues *Wwc2* siRNA induced embryo cell number/division phenotypes. **(A)** Experimental strategy for potential phenotypic rescue of clonal *Wwc2* KD by comparing co-microinjection (in one blastomere of 2-cell stage embryos) of GAP43-GFP mRNA (stable plasma membrane fluorescent microinjection marker to distinguish from the non-microinjected clone) with control siRNA, *Wwc2* siRNA or *Wwc2* siRNA + recombinant *HA-Wwc2* mRNA (containing N-terminal HA-epitope tag and point mutants conferring siRNA resistance – see Supplementary Figure S8) and assaying total, outer and inner-cell number at the 32-cell blastocyst stage. **(B)** Average clonal contribution (microinjected/Inj. versus non-microinjected/Non inj.) of outer and ICM/inner cells in control siRNA (blue bars), *Wwc2* siRNA (green bars) and *Wwc2* siRNA + *HA-Wwc2* mRNA (rescue – orange bars) microinjected embryos; errors represent s.e.m. and 2-tailed student *t*-test derived statistical significance between the experimental groups (asterisks), or clones within a group (double crosses) highlighted with statistical confidence intervals of *p* < 0.05 and *p* < 0.005 (denoted by one or two significance markers, respectively). The number of embryos in each experimental group is provided. **(C)** Exemplar confocal z-section micrographs of 32-cell blastocyst stage embryos, derived from the indicated microinjection groups, IF stained to detect HA-epitope tag expression (individual greyscale channels, plus merged images were the anti-HA channel is red and the DAPI cyan). Within the microinjected clone of the *Wwc2* siRNA treated embryo, the white asterisk denotes characteristic abnormal mitosis, whilst in the microinjected clone of the *Wwc2* siRNA + *HA-Wwc2* rescue embryo example the white arrows indicate anti-HA epitope tag IF signal associated with mid-bodies and the yellow arrow the single incidence of any persisting nuclear abnormality (see also indicated zoomed images - right); scale bar = 20μm. Supplementary Table ST24 summarise statistical analysis and individual embryo data used to generate the figure.

Incorporation of a HA-epitope tag within the confirmed *HA-Wwc2* phenotypic rescue mRNA enabled IF mediated verification of its translation and provided information on protein sub-cellular localisation within the microinjected clone. We consistently detected specific anti-HA immuno-reactivity (not observed in either control or *Wwc2* siRNA microinjected embryos) at structures resembling mitotic mid-bodies, particularly evident within microinjected clones at the 32-cell stage (Figure 6C; and in microinjected embryos at the 16-cell stage –Supplementary Figure S13a). A recent study identified an important role for uncharacteristically persistent interphase mid-bodies (referred to by the authors as ‘interphase microtubule bridges’) as important MTOCs within cleavage stage blastomeres (Zenker et al., 2017). Therefore, we assayed their number by IF staining for α-Tubulin and activated phospho-Aurora-B (pAURKB) as recognized midbody markers (Terada et al., 1998), in control and *Wwc2* KD embryos at the same 32-cell stage, but could not detect any statistically significant variation in their incidence (when corrected for reduced cell number caused by *Wwc2* KD – Supplementary Figures S13b–e). Hence, whilst *HA-Wwc2* derived recombinant protein associates with cell division mid-bodies (Figure 6C and Supplementary Figure S13a), the removal of endogenous *Wwc2* mRNA does not appear to impair mid-body formation or persistence following compromised cell divisions.

### Embryo *Wwc2* siRNA Knockdown Phenotypes Are Reversible by Expression of Recombinant *HA-Wwc2* mRNA

To confirm the uncovered embryo related roles of *Wwc2*, we attempted phenotypic rescue experiments using the same recombinant siRNA-resistant *HA-Wwc2* mRNA construct described above (i.e., oocyte IVM – Figure 4). We therefore repeated the marked clonal microinjection experiments but included a third condition in which *Wwc2* siRNA was co-microinjected with *HA-Wwc2* mRNA. Total and clonal cell contributions to inner and outer-cell populations, plus the incidence of abnormal nuclear morphology, were calculated in early and late blastocysts (Figure 6 and Supplementary Figure S12, respectively – Figure 6A and Supplementary Figure S12a detail the experimental strategies). Embryos microinjected with *Wwc2* siRNA alone exhibited the typical clone autonomous phenotypes. However, under *Wwc2* siRNA conditions supplemented with co-microinjected *HA-Wwc2* mRNA (expression of which was confirmed, see below and Figure 6C) the average number, clonal and spatial composition of constituent cells in early and late blastocysts was statistically indistinguishable from control siRNA microinjection groups (except for a small reduction in ICM contribution of the non-microinjected clone at the late blastocyst stage – from 9.5 ± 0.4 to 8.2 ± 0.3, Supplementary Figure S12). Additionally, a near complete rescue of abnormal morphologies was observed (with only one exception across all blastomeres assayed – shown in Figure 6C). These data demonstrate the specificity of the employed siRNA and confirm *Wwc2* as a novel regulatory gene of embryo cell division and subsequent cell fate.

## Discussion

We have identified *Wwc2*, the sole murine WWC-domain containing paralog of the recognized Hippo-pathway activator *Kibra* (Baumgartner et al., 2010; Genevet et al., 2010; Yu et al., 2010; Xiao et al., 2011a; Wennmann et al., 2014), as a novel regulator of preimplantation mouse embryo mitotic cell cleavages (Figures 1, 2 and Supplementary Figure S2) and oocyte meiotic maturation (Figure 3); the latter predicated on appropriate activation of AURKA (Figure 4 and Supplementary Figure S9). Additionally, in the embryo we show *Wwc2* KD cell clones are compromised in EPI lineage contribution and are prone to apoptosis or disproportionate PrE contribution (Figures 5, 6 and Supplementary Figures S10, S12). These data implicate at least two functional roles for *Wwc2* during early mouse development/reproduction; i. spatial and temporal regulation of germane meiotic and mitotic cell divisions [paradigms of typical and atypical acentrosomal cell division, relative to other mammalian species, respectively (Courtois et al., 2012; Bennabi et al., 2016; Severson et al., 2016; Gruss, 2018; Mogessie et al., 2018; Namgoong and Kim, 2018)], and ii. influencing blastomere pluripotency and differentiation, particularly relating to EPI and PrE formation (Figures 5, 6 and Supplementary Figures S10, S12). However, the full extent of the cell fate related role is difficult to experimentally dissect, given the described cell division defects, evident as early as the 8-cell stage.

Regarding cell fate regulation, active Hippo-signaling is known to support initial pluripotency and resist TE differentiation in ICM founders, by blocking YAP nuclear localisation (Sasaki, 2017). However, recently it has been shown that nuclear YAP translocation is later required for blastocyst EPI progenitors to compete with each other to achieve optimal naïve pluripotency (Hashimoto and Sasaki, 2019). Hence, depending on developmental context, active Hippo-signaling appears to both initially promote and latterly suppress relative pluripotency status. Our data, although compounded by the additional cell division phenotypes, suggests *Wwc2* may contribute to such Hippo-signaling mediated regulation of pluripotency. For example, we find ICM-residing late blastocyst *Wwc2* KD clones, although fewer than their non-microinjected clone counterparts, statistically favor contribution to PrE over EPI (unlike in control groups); indicative of impaired pluripotent potential (Figure 5). Whether this is caused by compromised Hippo-signaling activation directly after initial ICM founder internalization or is related to an inability to successfully compete for naïve pluripotency in the EPI, is not clear. However, increased apoptosis within ICM residing *Wwc2* KD clones, often marked by detectable SOX2 protein expression (but never GATA4 - Figures 5B,C), is consistent with the competitive elimination of initially pluripotent EPI progenitors unable to achieve/compete for naïve pluripotency. Indeed, analysis of early blastocyst stage *Wwc2* KD embryos/clones, suggests that the establishment of required differential Hippo-signaling between TE (i.e., suppression) and ICM (i.e., activation) was largely intact (Supplementary Figures S10, S11; see CDX2, PARD6B, CDH1 and YAP IF staining). Although, comparatively reduced (or sometimes absent) outer-cell CDX2 expression, plus less efficient inner-cell YAP protein nuclear exclusion, in ICM residing *Wwc2* KD clones (Figures 1, 5 and Supplementary Figures S10, S11) implies a degree of early onset mild Hippo-signaling dysregulation; potentially becoming more manifest during blastocyst maturation. EPI specific exclusion of *Wwc2* KD clones may also be related to increased aneuploidy caused by defective cell divisions, as derived experimental clonal aneuploidy in mouse chimeras is known to be selectively eliminated from the EPI by apoptosis but tolerated within extraembryonic blastocyst tissues (Bolton et al., 2016). The extent of the early cell fate effects may also reflect functional redundancy between WWC2 and KIBRA. Indeed, robust *Wwc2* KD does not affect *Kibra* mRNA levels (Supplementary Figure S3) and functional redundancy between WWC-domain proteins paralogs has been demonstrated in human cells [relating to KIBRA, WWC2 and WWC3 (Wennmann et al., 2014)]. Furthermore, the genetic ablation of the mouse *Kibra* gene is developmentally viable [adult mice only exhibit minor learning and memory defects (Makuch et al., 2011)]. However, if KIBRA and WWC2 proteins share functional redundancy during mouse preimplantation development, it is probably only in relation to potential Hippo-signaling regulation of cell fate and not applicable to the described cell division functions of *Wwc2* (as sole *Kibra* KD did not affect cell number – Figure 1 and Supplementary Figure S3).

Given that *Wwc2* specific siRNA embryo microinjections were performed at the 2-cell stage, it is unclear why cell autonomous division phenotypes are only manifest by the 16-cell stage (Figure 1 and Supplementary Figure S2). One possibility is that all post-microinjection cell-cycles are elongated in a manner only revealed at the whole embryo level by the 16-cell stage, implicating *Wwc2* as a regulator of the atypically long cell-cycles of preimplantation mammalian embryo blastomeres (reviewed O’farrell et al., 2004). The emergence of phenotypes at the 16-cell stage also coincides with the first spatial segregation of blastomeres (outer- and inner- positions), polarity/position dependant differential Hippo-signaling establishment and TE versus ICM cell fate initiation (Sasaki, 2017). It is tempting to speculate such developmental timing is particularly relevant in this context. However, the onset of individual nuclear morphological phenotypes, indicative of defective cell division/cytokinesis (mid-body association, bi-/multi-/micro-nucleation - Figure 2), were already evident by the 8-cell stage. Additionally, although often weak, outer-cell restricted CDX2 protein expression confirmed intact differential Hippo-signaling (Figures 1, 5 and Supplementary Figures S10, S11). Interestingly, whilst average cell number was impaired by *Wwc2* KD after the 8-cell stage, we noted it did not significantly increase again until the mid-blastocyst stage (remaining around 8–10 cells – Figure 1). Significantly, the mid-blastocyst stage marks the developmental point that atypical (compared to most other mammalian species) mouse embryo acentrosomal cell divisions revert back to centrosomal control, after *de novo* centrosome synthesis (Courtois et al., 2012). We propose our data invoke a role for WWC2 in regulating mouse preimplantation stage acentrosomal blastomere cleavage, supported by the uncovered nuclear morphology phenotypes associated with *Wwc2* KD (Figure 2) and readily detectable recombinant HA-WWC2 protein in mitotic mid-bodies up until the early blastocyst stage (but not easily detectable in late blastocysts - Figure 6 and Supplementary Figures S12, S13). We suspect HA-WWC2 association with mid-bodies reflects a role for WWC2 during preceding cell divisions and that the localisation represents a remnant of this role rather than affecting the previously described MTOC function of such atypically persistent mouse blastomere mid-bodies (Zenker et al., 2017); indeed, when corrected for lower overall cell numbers, the total number of detectable mid-bodies is not significantly different in *Wwc2* KD embryos from controls (Supplementary Figure S13). It is also possible the stagnant growth in embryo cell number between the 8-cell and mid-blastocyst stages simply reflects the life span of the microinjected *Wwc2*-specific siRNA. Consistently, our western blot analysis of WWC2 protein expression at the 32-cell stage revealed some WWC2 expression equivalent to 33% of control embryo expression (Figure 1E’). However, a similar expression level of WWC2 protein was also observed at the preceding 16-cell developmental time point (equal to 30% of control embryo levels –Supplementary Figure S14) suggesting such expression is most probably siRNA intransigent WWC2 protein (reflecting maternally inherited protein – MII oocytes express abundant WWC2 protein, see Figure 4B) rather than newly synthesized protein arising from the waning effect of the microinjected siRNA. Indeed, the impaired oocyte maturation data, by which GV oocytes [again in the absence of centrosomes (Bennabi et al., 2016; Severson et al., 2016; Gruss, 2018; Mogessie et al., 2018; Namgoong and Kim, 2018)] depleted of *Wwc2* derived transcripts and protein (initially incompletely) present with multiple spindle defects associated with failed AURKA activation (Figures 3, 4 and Supplementary Figures S5, S6, S9), adds extra support to our hypothesis. Interestingly, clonal *Aurkb* and *Aurkc* downregulation in preimplantation mouse embryos, respectively increases or decreases the rate of mitotic cell division (with corresponding over-expression exhibiting the opposing effects). Indeed, the reported cell number deficits associated with *Aurkc* downregulation, strongly resemble those associated with *Wwc2* KD (Li et al., 2017), suggesting a potential link between WWC2 and AURKB/AURKC function in the early embryo.

Regulation of mammalian Hippo-signaling by WWC-domain proteins was first demonstrated in human HEK293T cells, whereby direct binding of KIBRA was shown to activate LATS1/2, causing YAP phosphorylation (Xiao et al., 2011a). A subsequent report detailed AURKA/B directed and mitosis specific KIBRA phosphorylation, centered on a conserved serine residue (Ser539), and showed the KIBRA mutant (S539A) promotes precocious M-phase exit in models of spindle assembly check-point (SAC) mediated arrest (Xiao et al., 2011b). The same group later demonstrated full activation/phosphorylation of AURKA (hence, LATS2 activation and appropriate centrosomal localisation) requires KIBRA and that *KIBRA* KD causes spindle defects, lagging chromosomes and micronuclei formation (reminiscent of our uncovered *Wwc2* KD phenotypes), plus centrosomal fragmentation in human cell line models (Zhang et al., 2012). Interestingly, we have identified conserved AURK consensus motifs in both murine KIBRA and WWC2 proteins; including those paralogous to Ser539 (Supplementary Figure S15). Moreover, our western blot analysis of WWC2 protein expression during oocyte maturation suggests band shifts compatible with potential M-phase specific phosphorylation of WWC2 (Figure 4B). We therefore hypothesize the *Wwc2* KD phenotypes we observe here are mechanistically related to those described for human KIBRA, with the caveat they function on the level of MTOC regulation due to the absence of centrosomes (Courtois et al., 2012). For example, genetic ablations of either murine *Lats1* and *Lats2* do not cause embryo cell number deficits typical of *Wwc2* KD, nor such centrosome directed mitotic phenotypes described above, rather they present as ectopic ICM cell nuclear YAP localisations at the early blastocyst stage (Nishioka et al., 2009). Therefore, not all insights will be directly transferable. Notwithstanding and despite the lack of early mouse embryo centrosomes, key centrosome regulators are expressed and reported to regulate embryo cleavage divisions. For example, genetic knockout of Polo-like kinase 1 (*Plk1*), known to cooperate with AURKA within centrosomes to mediated bi-polar spindle formation (Asteriti et al., 2015), causes arrest at the same 8-cell stage at which global *Wwc2* KD embryos first exhibit cell division defects (Figures 1, 2 and Supplementary Figure S2; Lu et al., 2008). Moreover, an important role in blastomere division for the related *Plk4* gene, itself recognized as a key regulator of centriole formation/duplication (Bettencourt-Dias et al., 2005; Habedanck et al., 2005), has been demonstrated. Accordingly, active PLK4 protein localizes to chromatin proximal and coalescing MTOCs to promote microtubule nucleation and bi-polar spindle formation (Coelho et al., 2013). It will be interesting to investigate the identified *Wwc2* KD phenotypes in the context of such pre-existing and characterized MTOC-related factors and to test their applicability within the conceptually similar paradigm of acentrosomal meiotic oocyte maturation. Indeed, upon resumption of meiosis, PLK4 has been described to cooperate with AURKA (within MTOCs) to initiate microtubule nucleation around condensing chromosomes. Functional inhibition of PLK4 did not however block spindle assembly but increased assembly time and the individual MTOC regulatory roles of PLK4 and AURKA were shown to be only partially overlapping (Bury et al., 2017) and distinct from the co-existing chromosome derived RAN-GTP gradient driven mechanisms [as reviewed (Bennabi et al., 2016; Severson et al., 2016; Gruss, 2018; Mogessie et al., 2018; Namgoong and Kim, 2018)]. Importantly, the described functional inhibition of PLK4 was associated with eventual polar body formation (Bury et al., 2017), unlike after *Wwc2* KD (even after an extended IVM incubation time –Supplementary Figure S6). This indicates that whilst meiotic spindle assembly is impaired to varying degrees by *Wwc2* KD (Figures 3D,D’, 4D plus Supplementary Figures S5, S6, S9), other impediments to successful meiotic maturation must exist (potentially relating, although not necessarily limited, to spindle migration, SAC, cytokinesis, etc.). The comparative distribution of observed meiotic maturation phenotypes caused by *Wwc2* KD (i.e., failed spindle formation, defective spindles or persistent/non-dividing MI spindles – Figures 3D,D’, 4D plus Supplementary Figures S5, S6, S9) suggests some functional redundancy in response to loss of WWC2. This may be potentially related to the compensatory abilities of the three expressed AURK paralogs (*Aurka*, *Aurkb* and *Aurkc*), as demonstrated in combinatorial genetic ablations in mouse oocytes (Nguyen et al., 2018). Alternatively, the spectrum of phenotypes may reflect variability in the extent of *Wwc2* KD between individual oocytes. Indeed, western blot analysis of WWC2 protein levels in pooled *Wwc2*-specific siRNA microinjected oocytes only revealed reduced WWC2 expression at the point of IBMX removal (i.e., the GV stage) that then became undetectable by the MII equivalent stage; although even such reduced WWC2 levels were still associated with failed/undetectable AURKA phosphorylation/activation (Figure 4B).

In summary, we have identified the WWC-domain containing gene *Wwc2*, as an important novel regulator of cell division in both preimplantation mouse embryo blastomeres and oocytes; each paradigms of acentrosomal cell division. We have also uncovered evidence for *Wwc2*, as a paralog of the described Hippo-signaling activator *Kibra*, in participating in blastocyst cell fate regulation and pluripotency. Despite the compounding nature of the two identified *Wwc2* KD phenotypes, it will be of great future interest to further our molecular understanding of these newly identified roles of the *Wwc2* gene during early mouse development.

## Data Availability Statement

All datasets presented in this study are included in the article/Supplementary Material.

## Ethics Statement

The animal study was reviewed and approved by Ethics committee of Biology Centre (in České Budějovice) of the Czech Academy of Sciences (in accordance with Act No. 246/1992 issued by the Ministry of Agriculture of the Czechia 25.09.2014 – No. CZ02389).

## Author Contributions

AWB and GV designed experiments, participated in data analysis (plus AŠ), and drafted the manuscript (plus AŠ). GV performed all experiments and prepared samples for western blotting (performed by AŠ). PB derived the recombinant DNA constructs and LG performed important ancillary experiments, ultimately not included in the final manuscript. AWB coordinated the study. All authors approved the final version of the manuscript for publication.

## Conflict of Interest

The authors declare that the research was conducted in the absence of any commercial or financial relationships that could be construed as a potential conflict of interest.

## Supplementary Material

The supplementary materials comprise three components: (a) Supplementary Figures S1–S15, plus legends, (b) Supplementary Tables SM1–SM6, and (c) Supplementary Tables ST1–ST26 (detailing all analyzed embryo/oocyte cell counts and statistics).
