# Supplementary material for: Wwc2 Is a Novel Cell Division Regulator During Preimplantation Mouse Embryo Lineage Formation and Oogenesis
Source: Front Cell Dev Biol. 2020 Sep 17;8:857. doi: 10.3389/fcell.2020.00857 (PMC7527741; doi:10.3389/fcell.2020.00857)
Supplement: Supplementary file 1 — The supplementary materials comprise three components: (a) Supplementary Figures S1–S15, plus legends, (b) Supplementary Tables SM1–SM6, and (c) Supplementary Tables ST1–ST26 (detailing all analyzed embryo/oocyte cell counts and statistics). [file Data_Sheet_1.pdf]

SUPPLEMENTARY MATERIAL

***Wwc2* is a novel cell division regulator during preimplantation mouse embryo lineage formation and oogenesis.**

Virnicchi *et al.,* 2020

INC.

A) SUPPLEMENTARY FIGURES & LEGENDS: **FIG. S1 – FIG. S15**

B) SUPPLEMENTARY METHODS TABLES: **SM1 – SM6**

C) SUPPLEMENTARY TABLES (individual embryo cell counts & oocyte phenotypes + statistics): **ST1 – ST26**

A) <u>SUPPLEMENTARY FIGURES & LEGENDS: FIG. S1 – FIG. S15</u>

***Wwc2* is a novel cell division regulator during preimplantation mouse embryo lineage formation and oogenesis.**

Virnicchi *et al.,* 2020

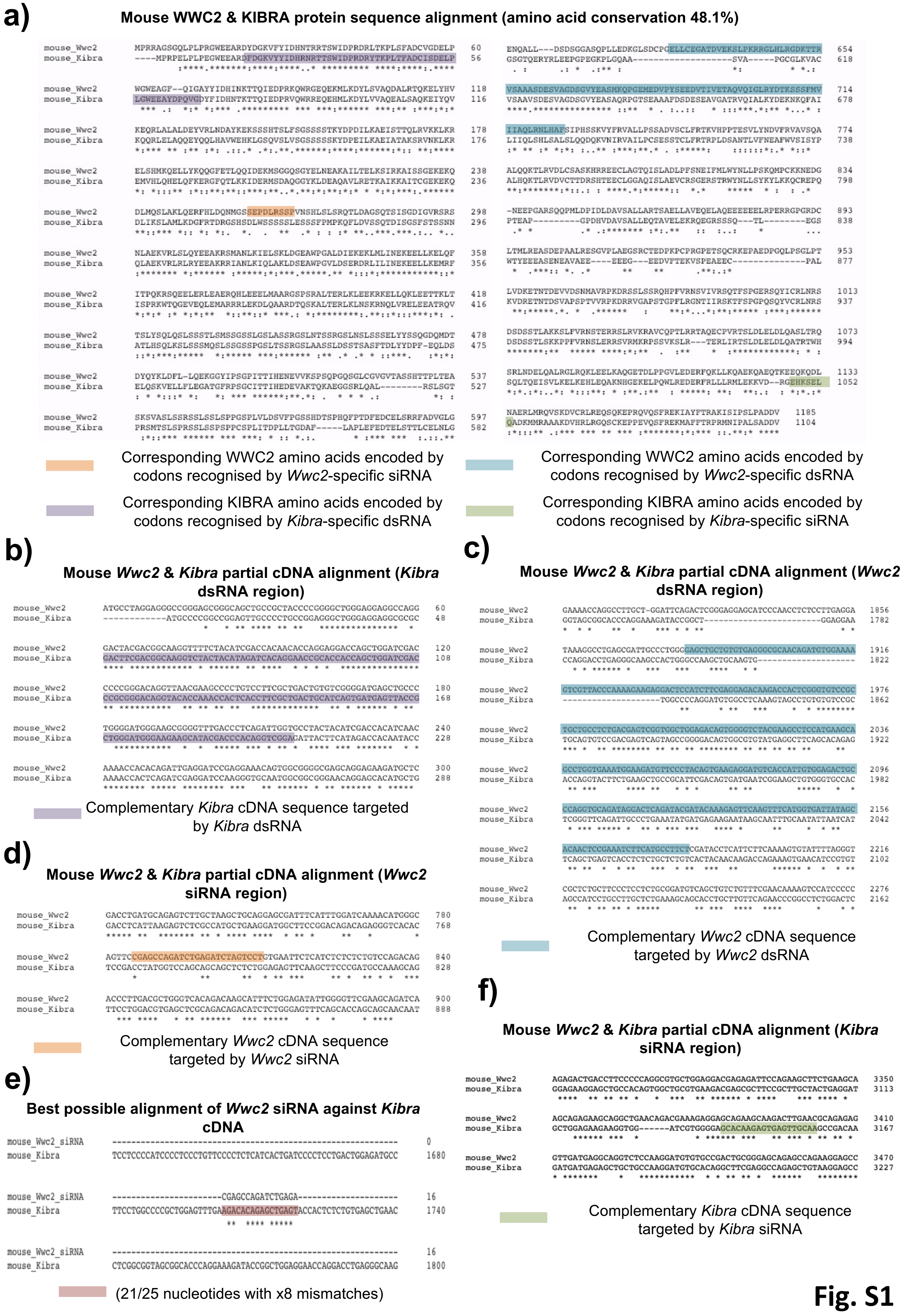
a)
Mouse WWC2 & KIBRA protein sequence alignment (amino acid conservation 48.1%)
Corresponding WWC2 amino acids encoded by codons recognised by Wwc2-specific siRNA
Corresponding KIBRA amino acids encoded by codons recognised by Kibra-specific dsRNA
Corresponding WWC2 amino acids encoded by codons recognised by Wwc2-specific dsRNA
Corresponding KIBRA amino acids encoded by codons recognised by Kibra-specific siRNA
b)
Mouse Wwc2 & Kibra partial cDNA alignment (Kibra dsRNA region)
Complementary Kibra cDNA sequence targeted by Kibra dsRNA
c)
Mouse Wwc2 & Kibra partial cDNA alignment (Wwc2 dsRNA region)
Complementary Wwc2 cDNA sequence targeted by Wwc2 dsRNA
d)
Mouse Wwc2 & Kibra partial cDNA alignment (Wwc2 siRNA region)
Complementary Wwc2 cDNA sequence targeted by Wwc2 siRNA
e)
Best possible alignment of Wwc2 siRNA against Kibra cDNA
(21/25 nucleotides with x8 mismatches)
f)
Mouse Wwc2 & Kibra partial cDNA alignment (Kibra siRNA region)
Complementary Kibra cDNA sequence targeted by Kibra siRNA

Fig. S1

Supplementary figure S1: Annotated amino acid and cDNA sequence alignments for mouse WWC2/*Wwc2* & KIBRA/*Kibra* detailing regions targeted by dsRNA and siRNA constructs. a) Full-length amino acid sequence of mouse WWC2 and KIBRA proteins; purple denotes the region encoded by codons targeted by the *Kibra*-specific dsRNA, orange that targeted by the *Wwc2*-specific siRNA, turquoise targeted by the *Wwc2*-specifc dsRNA and green targeted by *Kibra*-specific siRNA. **b)** Partial cDNA sequence alignment of the *Kibra* gene region coding sequence targeted by the *Kibra*-specific dsRNA (highlighted in purple), against the equivalent/aligned region in *Wwc2* cDNA. **c)** Partial cDNA sequence alignment of the region of the *Wwc2* gene coding sequence targeted by the *Wwc2*-specific dsRNA (highlighted in turquoise), against the equivalent/aligned region in *Kibra* cDNA. **d)** Partial cDNA sequence alignment of the *Wwc2* gene region sequence targeted by the *Wwc2*-specific siRNA (highlighted in orange), against the equivalent/aligned region in K*ibra* cDNA. **e)** The best result of aligning the used *Wwc2*-specific siRNA sequence against the mouse *Kibra* cDNA sequence (highlighted in red); note the best possible match of the *Wwc2-specific siRNA* sequence to the *Kibra* cDNA sequence is a 21 nucleotide stretch that contains eight evenly spread base-pair mis-matches, thus, minimising the chance of off-target recognition of *Kibra* transcripts by the *Wwc2*-designed siRNA. **f)** Partial cDNA sequence alignment of the *Kibra* gene region sequence targeted by the *Kibra*-specific siRNA (highlighted in green), against the equivalent/aligned region in K*ibra* cDNA. All alignments were conducted using default setting of the online Clustal-Ω multiple sequence alignment tool; asterisks (*) denote perfectly aligned and identical amino acids/DNA base-pairs, whereas colons (:) detail aligned conservative amino acid substitutions.

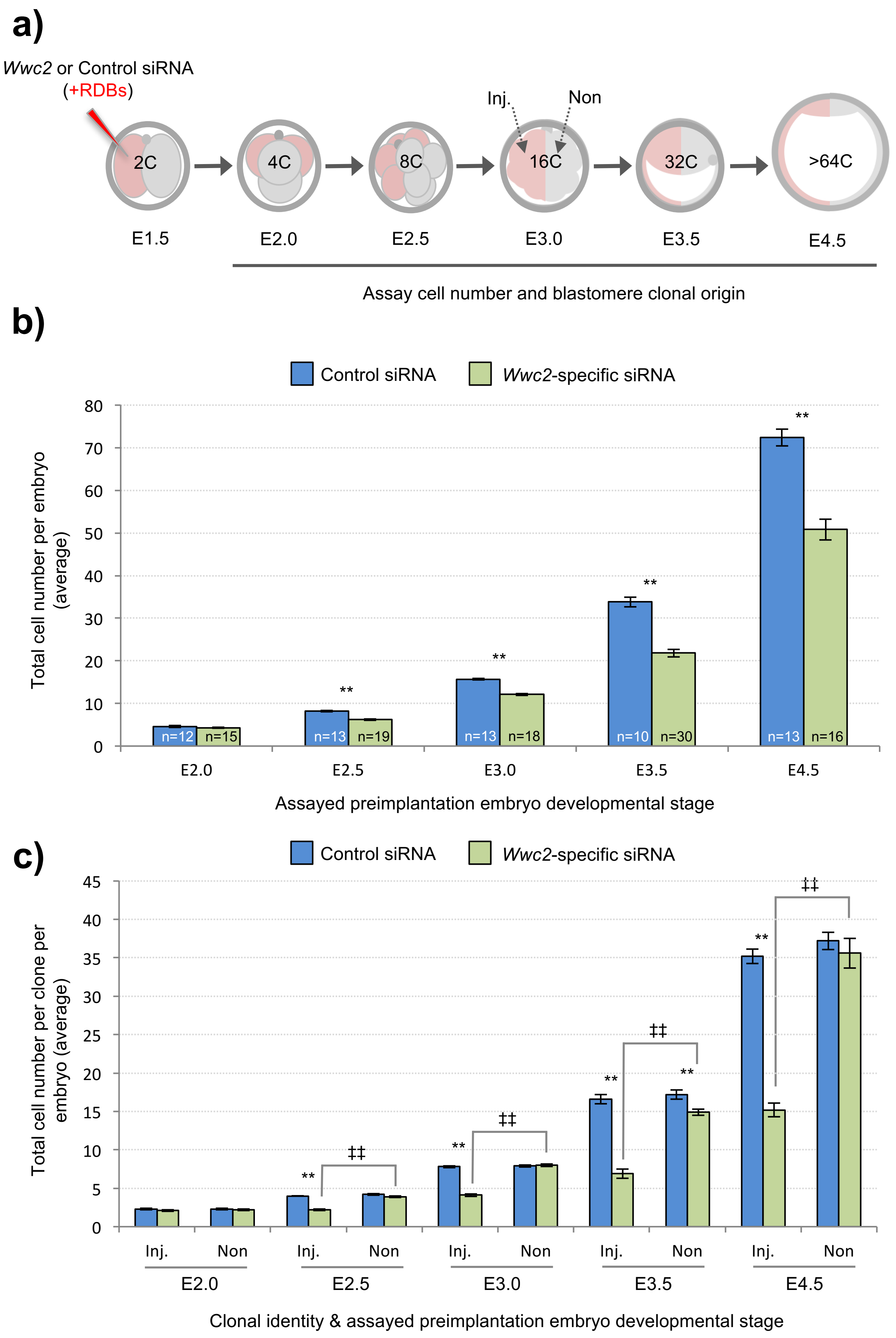
a)
Wwc2 or Control siRNA
(+RDBs)
2C
4C
8C
Inj.
Non
16C
32C
>64C
E1.5
E2.0
E2.5
E3.0
E3.5
E4.5
Assay cell number and blastomere clonal origin
b)
Control siRNA
Wwc2-specific siRNA
Total cell number per embryo (average)
80
70
60
50
40
30
20
10
0
**
n=12
n=15
n=13
n=19
n=13
n=18
n=10
n=30
n=13
n=16
E2.0
E2.5
E3.0
E3.5
E4.5
Assayed preimplantation embryo developmental stage
c)
Control siRNA
Wwc2-specific siRNA
Total cell number per clone per embryo (average)
45
40
35
30
25
20
15
10
5
0
‡‡
**
Inj.
Non
E2.0
E2.5
E3.0
E3.5
E4.5
Clonal identity & assayed preimplantation embryo developmental stage

Fig. S2

Supplementary figure S2: Preimplantation embryo *Wwc2* KD associated cell division phenotypes are cell autonomous. a) Experimental strategy for *Wwc2* KD in a marked clone (representing 50% of the embryo) by co-microinjection (in one blastomere of 2-cell stage embryos) of *Wwc2* siRNA and RDBs (fluorescent lineage marker – Inj.) and an assay of clonal contribution (Inj vs. Non) at stated developmental stages (compared to similar control siRNA microinjections). **b)** Average total number of cells per embryo (irrespective of clonal origin), at each developmental stage, from control siRNA (blue bars) and *Wwc2*-specifc siRNA microinjected (green bars) embryos. The number of embryos in each experimental group is shown. **c)** As in b) but detailing the clonal contribution of cells (Inj vs. Non) to total embryo cell number in control siRNA (blue bars) or *Wwc2* siRNA (green bars) microinjected embryos. In panels b) & c) chart error bars represent s.e.m. and statistically significant differences (2-tailed students t-test) between the experimental microinjection groups (asterisks) or clones within a group (double crosses) are highlighted with statistical confidence intervals of $p<0.05$ and $p<0.005$ (denoted by one or two significance markers, respectively). Supplementary tables ST7-ST11 summarise statistical analysis and individual embryo data.

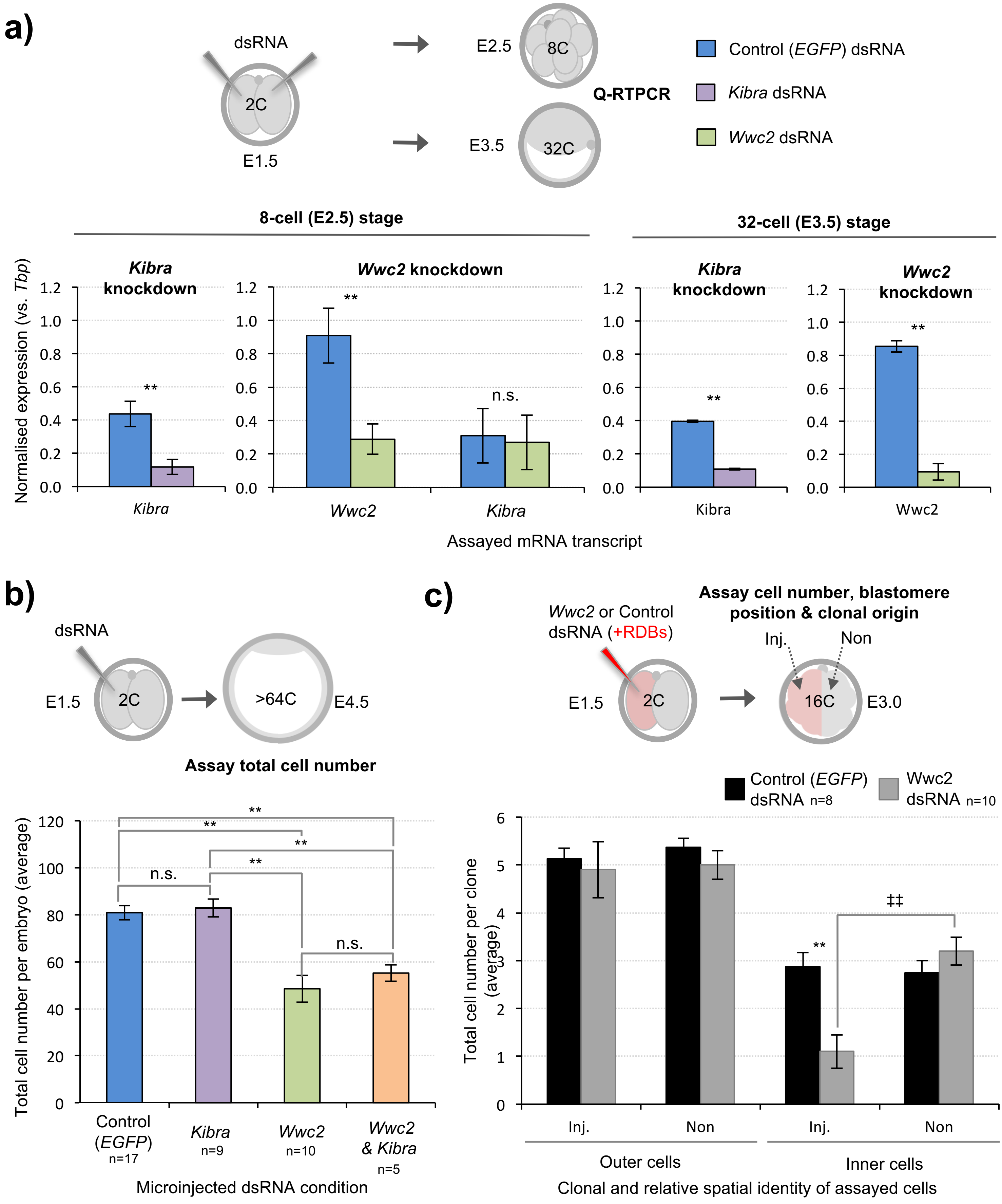
a)
dsRNA
2C
E1.5
E2.5
8C
E3.5
32C
Q-RTPCR
Control (EGFP) dsRNA
Kibra dsRNA
Wwc2 dsRNA
8-cell (E2.5) stage
32-cell (E3.5) stage
Normalised expression (vs. Tbp)
Kibra knockdown
Wwc2 knockdown
**
n.s.
Kibra
Wwc2
Assayed mRNA transcript
b)
dsRNA
E1.5
2C
>64C
E4.5
Assay total cell number
Total cell number per embryo (average)
n.s.
**
Control (EGFP) n=17
Kibra n=9
Wwc2 n=10
Wwc2 & Kibra n=5
Microinjected dsRNA condition
c)
Wwc2 or Control dsRNA (+RDBs)
Assay cell number, blastomere position & clonal origin
Inj.
Non
E1.5
2C
16C
E3.0
Control (EGFP) dsRNA n=8
Wwc2 dsRNA n=10
Total cell number per clone (average)
‡‡
Outer cells
Inner cells
Clonal and relative spatial identity of assayed cells

Fig. S3

Supplementary figure S3: dsRNA mediated WWC-domain containing gene (*Kibra* & *Wwc2*) KD in preimplantation mouse embryos is associated (*Wwc2*) reduced cell number. a) Experimental schema of *Kibra* and *Wwc2* specific dsRNA (plus *EGFP n*egative control) microinjection mediated global embryo KD, followed by culture to either 8-cell or 32-cell stages and Q-RT-PCR analysis (upper). Normalised expression of either *Kibra* or *Wwc2* derived mRNAs after stated dsRNA microinjection, at stated developmental stages (lower; error bars represent s.e.m., n=3 and ** denotes p<0.005 in 2-tailed student t-test, n.s. denotes lack of significance). **b)** Experimental schema describing clonal *Kibra*, *Wwc2* or *Kibra*+*Wwc2* KD (microinjection of one cell at the 2-cell stage) and total fixed embryo cell number assay at the late blastocyst (>64-cell) stage (upper). Average total cell number per embryo in each stated dsRNA microinjection group (lower chart). **c)** Experimental strategy to KD *Wwc2* expression (as in b) in marked clones, (*Wwc2* dsRNA and rhodamine conjugated dextran microbeads/RDBs co-microinjection) and assay clonal contribution at the 16-cell stage (upper). Average total cell number per clone (Inj. vs. Non) in *Wwc2* or control dsRNA microinjection groups, as determined by relative position of individual cells within the embryo (encapsulated inside or with a cell contactless domain on outside; lower chart). In panels b) & c) chart error bars represent s.e.m. with indicated 'n' numbers. Highlighted statistically significant differences (2-tailed students t-test) between experimental microinjection groups (asterisks), or clones (Inj. vs. Non) within a group (double crosses), with statistical confidence intervals of p<0.05 and p<0.005, denoted by one or two significance markers, respectively; in panel b) a lack of significant difference between compared groups is marked 'n.s.'. Supplementary tables ST12 & ST13 summarise statistical analysis and individual embryo data.

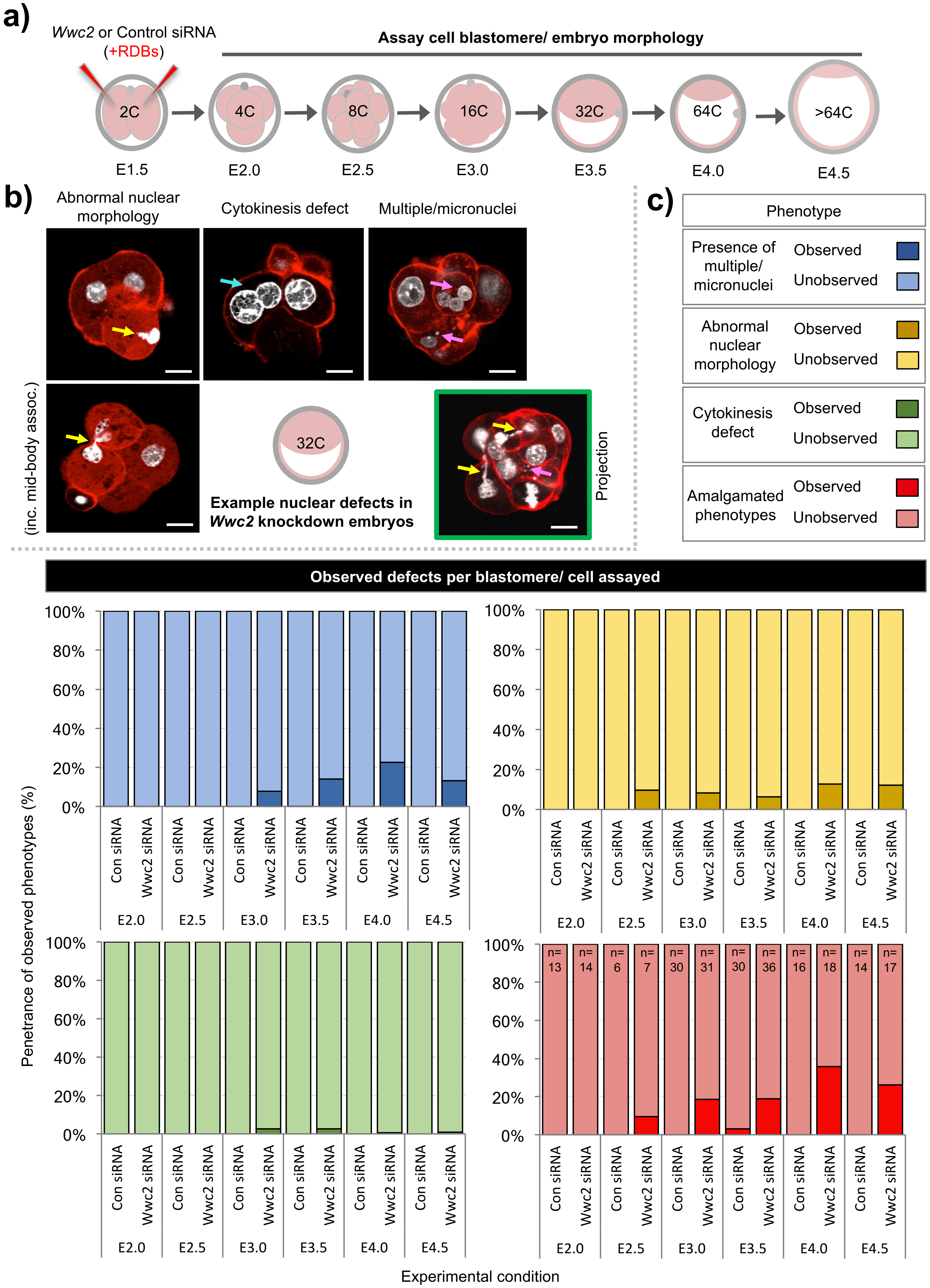
a)
Wwc2 or Control siRNA
(+RDBs)
Assay cell blastomere/ embryo morphology
2C
4C
8C
16C
32C
64C
>64C
E1.5
E2.0
E2.5
E3.0
E3.5
E4.0
E4.5
b)
Abnormal nuclear morphology
Cytokinesis defect
Multiple/micronuclei
(inc. mid-body assoc.)
32C
Example nuclear defects in Wwc2 knockdown embryos
Projection
c)
Phenotype
Presence of multiple/ micronuclei
Observed
Unobserved
Abnormal nuclear morphology
Observed
Unobserved
Cytokinesis defect
Observed
Unobserved
Amalgamated phenotypes
Observed
Unobserved
Observed defects per blastomere/ cell assayed
Penetrance of observed phenotypes (%)
100%
80%
60%
40%
20%
0%
Con siRNA
Wwc2 siRNA
E2.0
E2.5
E3.0
E3.5
E4.0
E4.5
n= 13
n= 14
n= 6
n= 7
n= 30
n= 31
n= 30
n= 36
n= 16
n= 18
n= 14
n= 17
Experimental condition

Fig. S4

Supplementary figure S4: Global *Wwc2* KD mediated total embryo cell number deficits are associated with defective cell division morphologies (according to incidence in each assayed blastomere/cell). a) Schematic of experimental design; 2-cell stage embryos microinjected with control or *Wwc2*-specific siRNA, cultured to indicated developmental stages, fixed and assayed for total cell number or the incidence of abnormal cell morphology indicative of defective cell division (determined by DAPI and rhodamine conjugated phalloidin staining - note, data derived from same experiments as those described in Figs. 1 & 2). **b)** Exemplar confocal micrograph z-sections of *Wwc2*-specific siRNA microinjected embryos at the 32-cell stage, illustrating three distinct phenotypic morphological defect categories observed; i. abnormal nuclear morphology (including chromatin mid-body association, highlighted by yellow arrows - left), ii. cytokinesis defects defined by two nuclei per cell (highlighted by blue arrow -centre), iii. presence of multiple (*i.e.* >3, highlighted by pink arrows) nuclei and/or micronuclei per cell (right) and iv. composite of categorised defects highlighted by colour coded arrows in a single embryo confocal z-section projection (green boarder). DAPI (white) and cortical F-actin (red); scale bar = 20 μm. **c)** Frequencies of each observed category of phenotypic/ morphological defect (*i.e.* micronuclei; blue, abnormal nuclei; yellow and failed cytokinesis; green) or a combination of at least two or more (red) in each assayed blastomere/cell of control siRNA and *Wwc2*-specific siRNA microinjected embryos, at indicated developmental stages (numbers of embryos in each group highlighted, see amalgamated data - red); data demonstrate defect incidence per assayed blastomere/cell (not in at least one blastomere/cell per embryo, as in Fig. 2). Supplementary tables ST1-ST6 summarise statistical analysis and individual embryo data in each experimental group.

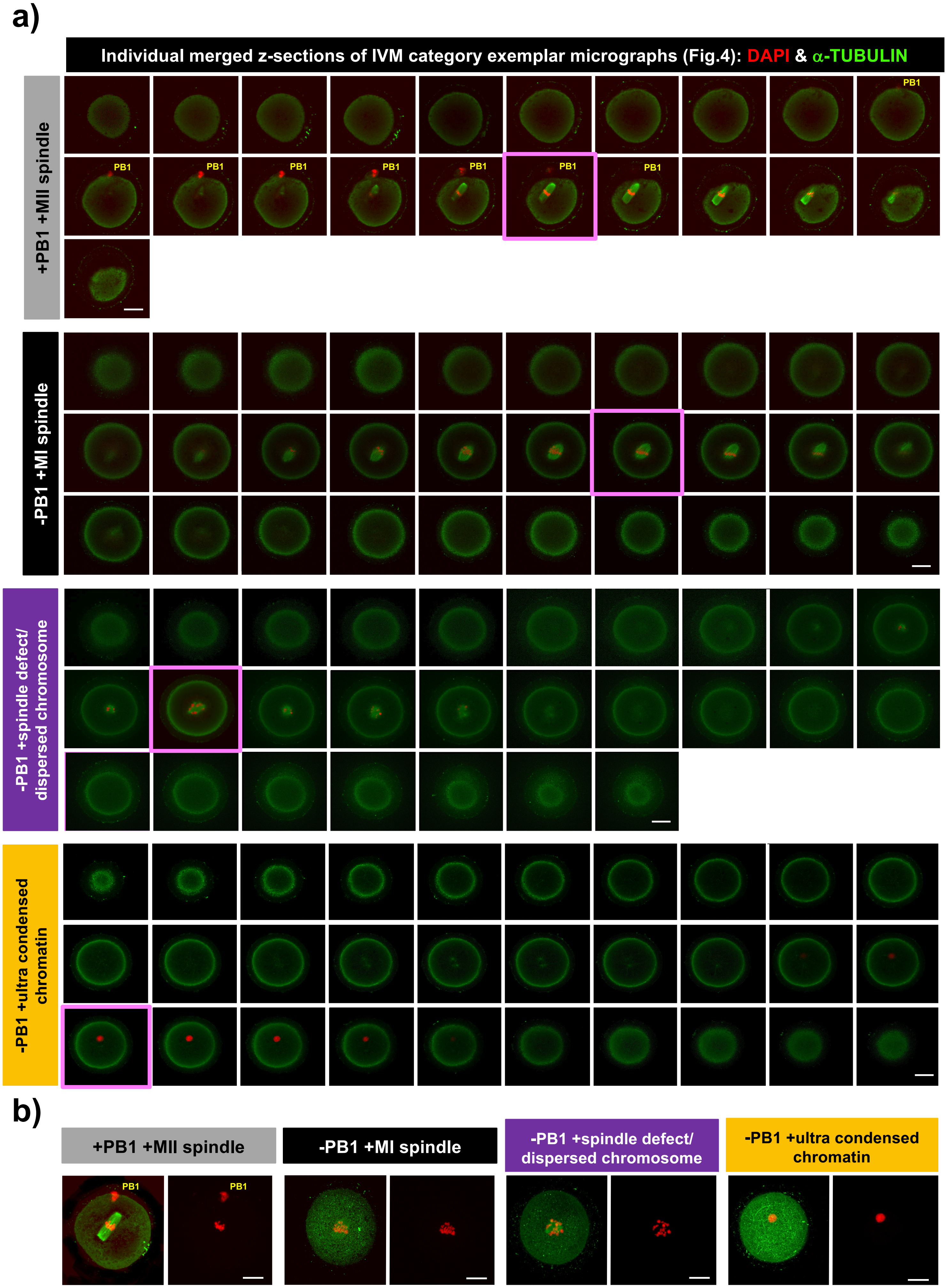
a)
Individual merged z-sections of IVM category exemplar micrographs (Fig.4): DAPI & α-TUBULIN
+PB1 +MII spindle
PB1
-PB1 +MI spindle
-PB1 +spindle defect/ dispersed chromosome
-PB1 +ultra condensed chromatin
b)
+PB1 +MII spindle
-PB1 +MI spindle
-PB1 +spindle defect/ dispersed chromosome
-PB1 +ultra condensed chromatin
PB1

Fig. S5

Supplementary figure S5: Exemplar confocal micrographs of *Wwc2*-specific siRNA induced mouse oocyte in vitro maturation phenotypes (complete z-series; related to Figure 3). a) Full merged channel (DAPI-stained DNA in pseudo-coloured red and immuno-stained α-tubulin in green) confocal micrograph z-series of individual oocytes illustrating the categories of the IVM phenotypes (see labels on right) elicited by *Wwc2* knockdown (or control siRNA microinjection) as described in Fig. 3 (*i.e.* these are the same oocytes as depicted in the single z-section micrographs of Fig. 3d'); top rows (grey label) indicating successful IVM to the MII arrested stage (+PB1 +MII spindle), second rows (black label) indicating formation of a meiotic spindle without polar body extrusion -PB1 +MI spindle), third rows (purple label) indicating meiotic spindle defects with non-aligned/dispersed chromosomes and absent polar body extrusion (-PB1 +spindle defect/ dispersed chromosomes) and the last rows (yellow label) indicating failed meiotic spindle formation and the presence of condensed chromatin in the absence of a polar body (-PB1 +ultra condensed chromatin). The individual z-stack micrographs that are depicted in Fig. 3d' are framed in magenta. PB1 denotes the first meiotic polar body and the scale bar = 20 μm. **b)** Confocal z-series projections of the oocyte individual z-series depicted in panel a), as DAPI + α-tubulin merges (left) and DAPI alone (right) channels. In all micrographs PB1 denotes the first meiotic polar body and the scale bar = 20 μm.

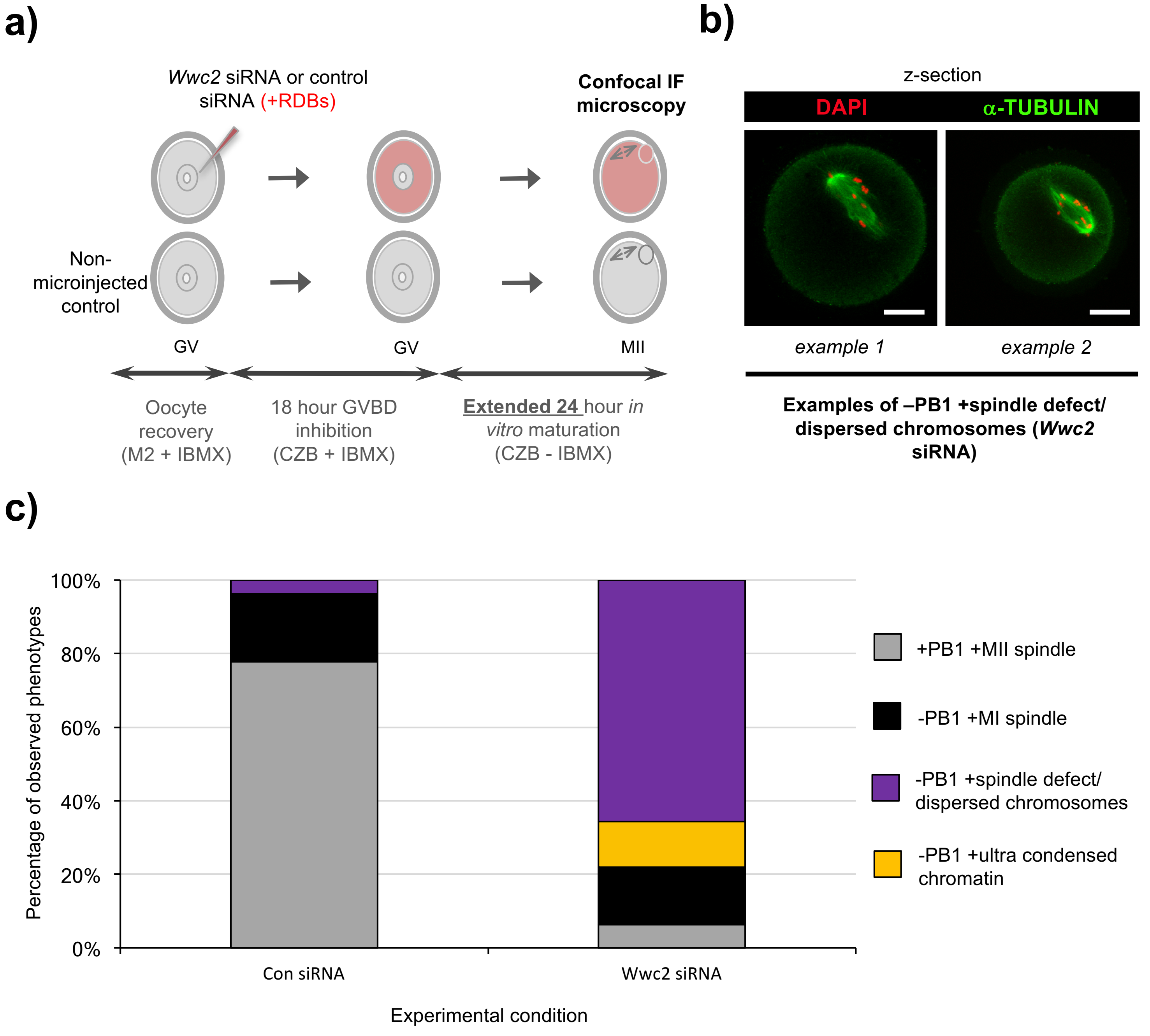
a)
Wwc2 siRNA or control siRNA (+RDBs)
Confocal IF microscopy
Non-microinjected control
GV
GV
MII
Oocyte recovery (M2 + IBMX)
18 hour GVBD inhibition (CZB + IBMX)
Extended 24 hour in vitro maturation (CZB - IBMX)
b)
z-section
DAPI
α-TUBULIN
example 1
example 2
Examples of –PB1 +spindle defect/ dispersed chromosomes (Wwc2 siRNA)
c)
100%
80%
60%
40%
20%
0%
Percentage of observed phenotypes
Con siRNA
Wwc2 siRNA
Experimental condition
+PB1 +MII spindle
-PB1 +MI spindle
-PB1 +spindle defect/ dispersed chromosomes
-PB1 +ultra condensed chromatin

Fig. S6

Supplementary figure S6: Meiotic *in vitro* maturation phenotypes of *Wwc2* KD GV oocytes are persistent (up to 24 hours post-IBMX) a) Experimental schema of *Wwc2* transcript KD in GV oocytes by microinjected *Wwc2* siRNA (plus control siRNA groups), 18 hour incubation in IMBX containing media (preventing GVBD) and extended 24 hour IVM (minus IMBX treatment), followed by IF and confocal microscopic analysis; co-microinjected RDBs were used as injection control marker. **b)** Exemplar single z-section confocal micrographs (two) of *Wwc2* siRNA induced and extended (24 hours) IVM phenotypes, classified as "-PB1 +spindle defect/dispersed chromosomes". DNA is counter-stained with DAPI (pseudo-coloured red) and microtubules IF stained with anti-α-Tubulin antibodies (green); scale bar = 20μm. **c)** Chart detailing successful IVM maturation frequencies of control siRNA (Con siRNA) and microinjected *Wwc2* siRNA oocytes, to the MII stage or preceding phenotypic stages, as defined/illustrated in Fig. 3d & d' (including the predominant -PB1 +spindle defect/dispersed chromosomes phenotype observed - panel b). Supplementary tables ST15 summarise statistical analysis and individual oocyte data in each experimental group.

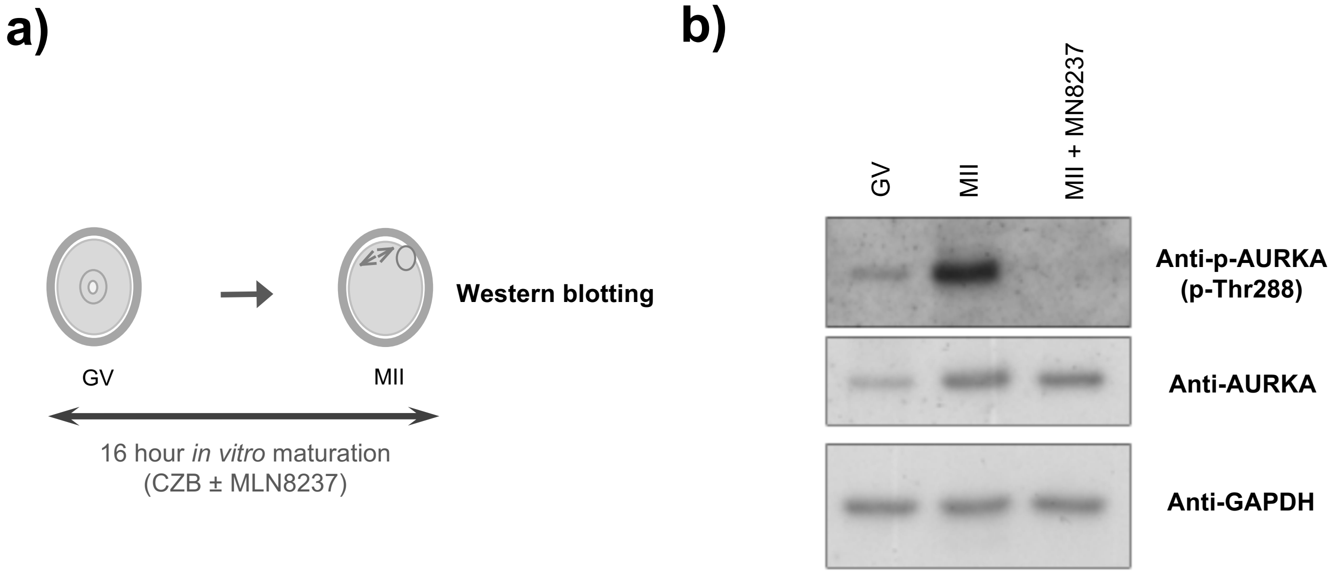
a)
Western blotting
GV
MII
16 hour *in vitro* maturation
(CZB ± MLN8237)
b)
GV
MII
MII + MN8237
Anti-p-AURKA
(p-Thr288)
Anti-AURKA
Anti-GAPDH

Fig. S7

Supplementary figure S7: anti-p-AURKA (p-Thr288) antibody specificity verification. a) Experimental strategy to verify specificity of the anti-p-AURKA (p-Thr288) used in western blotting experiments. Recovered GV stage primary oocytes were directly processed for western blotting (as described in the materials and methods) or immediately following *in vitro* maturation (IVM) to the MII arrested stage; in the presence or absence of the confirmed AURKA inhibitor, MLN8237 (1μM: Pollard and Mortimore, 52: 2629-2651, 2009) - MLN8237 blocks auto-phosphorylation/activation of AURKA (Katsha *et al.,* 8:1419-1428, 2014). **b)** Western blots showing activated/phosphorylated p-AURKA (upper panel; probed with anti-p-AURKA/ p-Thr288 primary antibody – Cell Signalling Technology, cs2914), total AURKA (middle panel – BD Biosciences, 610938) or house-keeping control GAPDH (lower panel – Merck, G9545) protein expression levels in freshly recovered GV stage primary oocytes (first lane), IVM MII-arrested secondary oocytes (middle lane) or oocytes subject to IVM in the presence of MLN8237.

Partial mouse *Wwc2* cDNA sequence (*Wwc2* siRNA target region)

```
GACCTGATGCAGAGTCTTGCTAAGCTGCAGGAGCGATTTCATTTGGATCAAAACATGGGC   780
AGTTCCGAGCCAGATCTGAGATCTAGTCCTGTGAATTCTCATCTCTCTCTGTCCAGACAG   840
```

Complementary *Wwc2* cDNA sequence targeted by *Wwc2* siRNA

```
AGT TCC GAG CCA GAT CTG AGA TCT AGT CCT GTG
 S   S   E   P   D   L   R   S   S   P   V
259                                     269
```

Amino acid codon information

```
AGT TCC GAG CCt GAT tta AGg TCT tcT CCT GTG
 S   S   E   P   D   L   R   S   S   P   V
259                                     269
```

Introduced silent mutations in siRNA resistant recombinant *HA-Wwc2* cDNA construct (lowercase)

Fig. S8

Supplementary figure S8: Information on site directed mutagenesis design to generate siRNA-resistant *HA-Wwc2* 'rescue' construct (microinjected as a mRNA). Partial cDNA sequence of mouse genome derived *Wwc2* transcript (upper panel), onto which the recognition sequence of the *Wwc2*-specific siRNA used in this study is highlighted (orange: *i.e.* positions 786-813 bp); a plasmid clone of which was generated during this study (*i.e.* pGEM-T-Easy:HA-Wwc2). Expanded representations of this region, detailing the corresponding amino acid codons (delineated by spaces) and the coded residues in the WWC2 protein (as specified using the single letter code), are provided below. The upper expanded depiction describes the endogenous wild-type *Wwc2* sequence and the lower representation details how this was altered by site directed mutagenesis to yield a mRNA construct that, when *in vitro* transcribed, would be insensitive to RNAi mediated degradation when employing our *Wwc2*-specific siRNA (as there would be insufficient base-pair complementarity). The mutated bases are denoted by lower-case font and occur within the would be recognised *Wwc2*-specific siRNA motif but due to the redundancies in the genetic code, do not alter any amino acid coding information. Hence the only distinguishing feature of protein derived from the siRNA resistant mutated recombinant construct/transcript, in relation to its endogenous counterpart, would be the incorporation of the N-terminal HA-epitope tag.

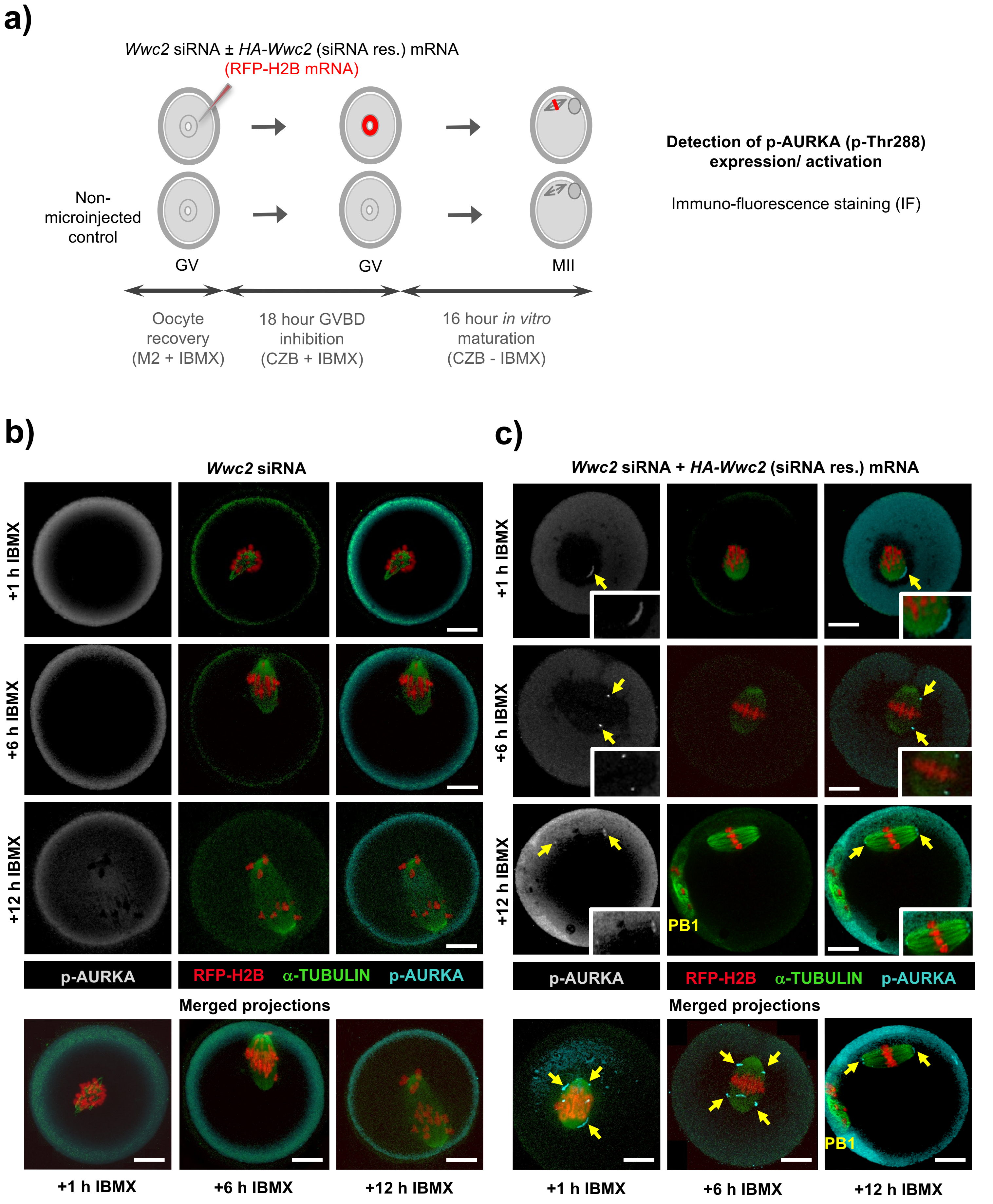
a)
Wwc2 siRNA ± HA-Wwc2 (siRNA res.) mRNA
(RFP-H2B mRNA)
Non-microinjected control
GV
GV
MII
Oocyte recovery (M2 + IBMX)
18 hour GVBD inhibition (CZB + IBMX)
16 hour in vitro maturation (CZB - IBMX)
Detection of p-AURKA (p-Thr288) expression/ activation
Immuno-fluorescence staining (IF)
b)
Wwc2 siRNA
+1 h IBMX
+6 h IBMX
+12 h IBMX
p-AURKA
RFP-H2B α-TUBULIN p-AURKA
Merged projections
+1 h IBMX
+6 h IBMX
+12 h IBMX
c)
Wwc2 siRNA + HA-Wwc2 (siRNA res.) mRNA
+1 h IBMX
+6 h IBMX
+12 h IBMX
PB1
p-AURKA
RFP-H2B α-TUBULIN p-AURKA
Merged projections
PB1
+1 h IBMX
+6 h IBMX
+12 h IBMX

Fig. S9

Supplementary figure S9: Phenotypic rescue of *Wwc2* KD induced GV oocyte IVM spindle defects by co-microinjection of siRNA resistant *HA-Wwc2* mRNA. a) Experimental schema of GV oocyte microinjection conditions; *i.e. Wwc2* siRNA alone or *Wwc2* siRNA + *HA-Wwc2* (siRNA resistant) mRNA, each co-microinjected with RFP-H2B mRNA (injection marker). Microinjected oocytes were incubated in IMBX containing media (18 hours - preventing GVBD), subject to IVM (max. 16 hours – media minus IMBX) and processed (at the indicated time-points) for IF assaying of phospho-Aurora (p-AURKA) and α-TUBULIN expression/localisation. **b)** Exemplar z-section confocal micrographs of *Wwc2* siRNA alone injected oocytes after IVM to indicated time-points (post IBMX removal). Detected p-AURKA (greyscale; cyan in merge) and α-TUBULIN (green) expression/localisation, plus the RFP-H2B microinjection reporter (red) are shown. Projected confocal micrographs of colour merged images are depicted at each time-point (lower panels). **c)** As in b) but pertaining to the *Wwc2* siRNA + *HA-Wwc2* (siRNA resistant) mRNA microinjection embryo group. Yellow arrows denote detectable and spindle proximal p-AURKA (p-Thr288) immuno-reactivity (enlarged in the greyscale and merged channel insets); PB1 denotes the extruded first meiotic polar body. In panels b) and c) the scale bar = 20 μm.

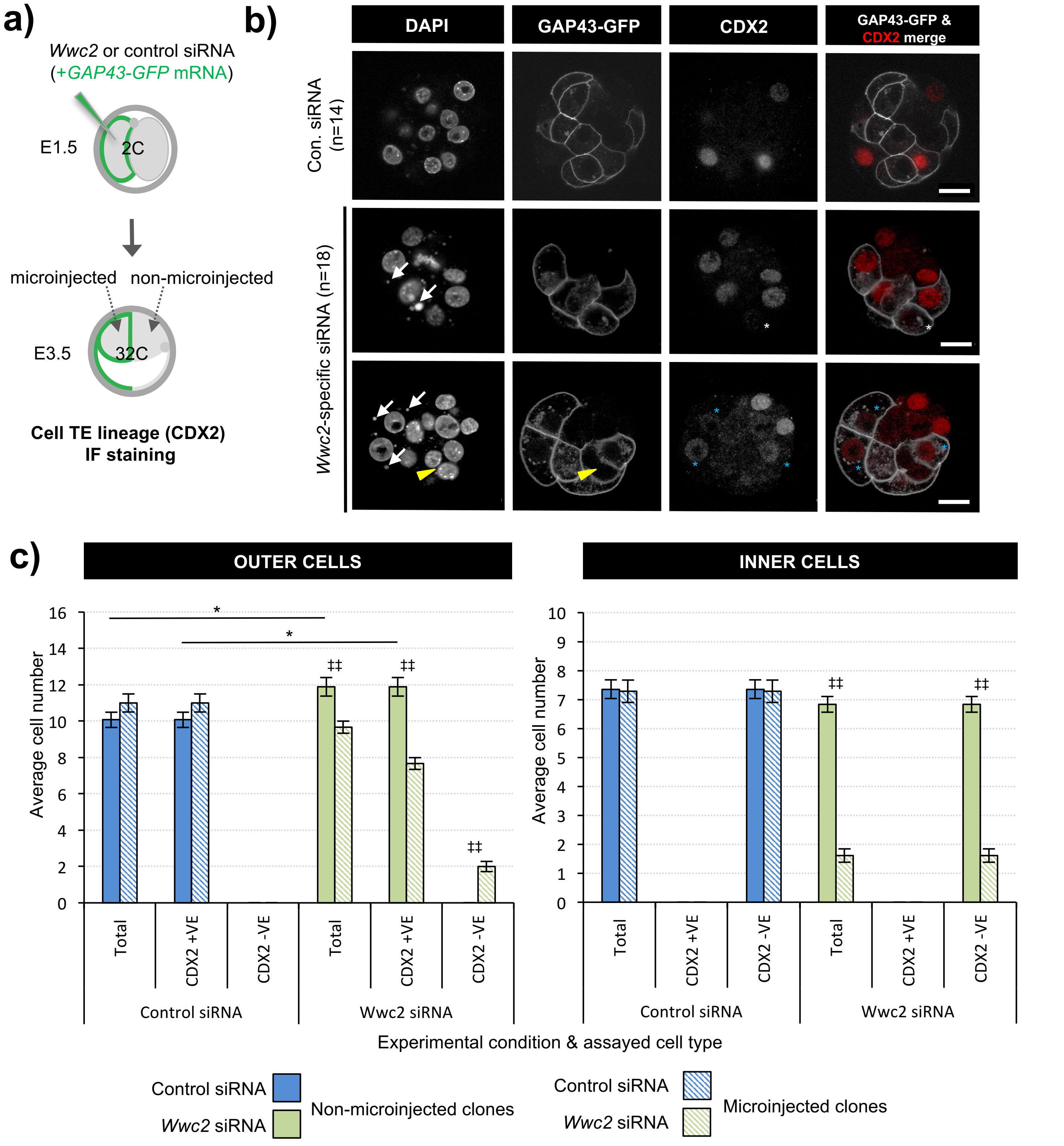
a)
Wwc2 or control siRNA
(+GAP43-GFP mRNA)
E1.5
2C
microinjected
non-microinjected
E3.5
32C
Cell TE lineage (CDX2)
IF staining
b)
DAPI
GAP43-GFP
CDX2
GAP43-GFP & CDX2 merge
Con. siRNA (n=14)
Wwc2-specific siRNA (n=18)
c)
OUTER CELLS
INNER CELLS
Average cell number
Total
CDX2 +VE
CDX2 -VE
Control siRNA
Wwc2 siRNA
Experimental condition & assayed cell type
Control siRNA
Wwc2 siRNA
Non-microinjected clones
Control siRNA
Wwc2 siRNA
Microinjected clones

Fig. S10

Supplementary figure S10: Outer trophectoderm (TE) versus ICM formation in clonal *Wwc2* gene KD at early blastocyst stage. a) Experimental strategy for *Wwc2* expression KD in marked clones (representing 50% of embryo) by co-microinjection (in one blastomere of 2-cell stage embryos) of *Wwc2* siRNA and GAP43-GFP mRNA (stable plasma membrane fluorescent injection clone marker – Inj.) and IF based assay of clonal contribution (microinjected. versus non-microinjected.) to CDX2 (TE marker) positive or negative cells, plus outer and inner-cell embryo populations at 32-cell stage (versus control siRNA microinjections). **b)** Exemplar confocal z-section micrographs of IF stained blastocysts (individual greyscale channels plus merged image; CDX2 is red) from control and *Wwc2* siRNA microinjected groups. Note, white arrows and yellow arrow head denote DAPI stained micronuclei and a bi-nucleated cell, respectively, uniquely found in *Wwc2* siRNA microinjected clones. Blue and white asterisks denote respective low level (compared to non-injected clone) or undetectable outer-cell CDX2 protein expression in *Wwc2* KD clones. The number of embryos in each group is provided; scale bar equals 20μm. **c)** Bar charts describing the average clonal (non-microinjected – solid bars, versus microinjected – hatched bars) contribution of cells from embryos treated/microinjected with control (blue bars) or *Wwc2* siRNA (green bars) to either outer cell or inner cell (ICM) cell populations and cells positive or negative for CDX2 protein expression (IF – as shown in panel b) – same n-numbers). Errors represent s.e.m. and 2-tailed student t-test derived statistical significance between control and *Wwc2* KD groups (asterisks), or clones (non-microinjected vs microinjected) within a group (double crosses) are highlighted with statistical confidence intervals of $p<0.05$ and $p<0.005$, as denoted by one or two significance markers, respectively. Supplementary tables ST23 summarise statistical analysis and individual embryo data.

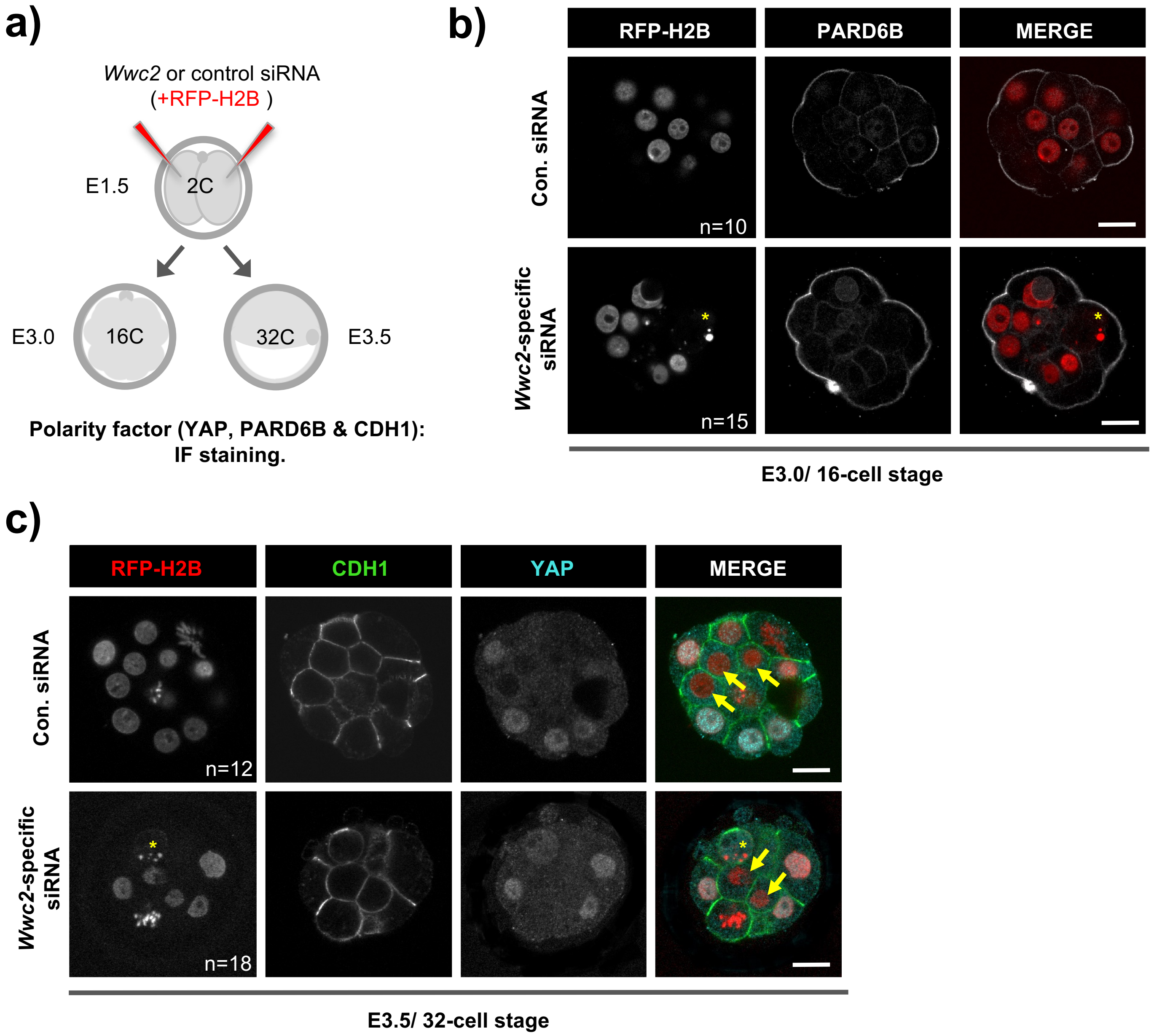
a)
Wwc2 or control siRNA
(+RFP-H2B)
E1.5
2C
E3.0
16C
32C
E3.5
Polarity factor (YAP, PARD6B & CDH1):
IF staining.
b)
RFP-H2B
PARD6B
MERGE
Con. siRNA
n=10
Wwc2-specific siRNA
n=15
E3.0/ 16-cell stage
c)
RFP-H2B
CDH1
YAP
MERGE
Con. siRNA
n=12
Wwc2-specific siRNA
n=18
E3.5/ 32-cell stage

Fig. S11

Supplementary figure S11: *Wwc2* KD does not affect outer-cell apical-basolateral polarisation nor differential inner versus outer-cell Hippo-signalling pathway activity. a) Experimental strategy to assay apical-basolateral polarity factor (PARD6B and CDH1) and Hippo-pathway effector (YAP) protein expression in 16- or 32-cell stage embryos derived from 2-cell stage embryos co-microinjected in both blastomeres with *Wwc2* (or control) siRNA and mRNA for fluorescent histone H2B fusion protein (RFP-H2B). **b)** Representative confocal z-section micrographs of control and *Wwc2* siRNA microinjected embryos IF stained at the 16-cell stage for PARD6B expression. Individual fluorescent channels are shown in greyscale and merged (in which the RFP-H2B signal is red). **c)** As in b) although embryos are assayed at the 32-cell stage and double-IF stained for CDH1 and YAP (respectively, green and cyan in merged images). Yellow arrows denote inner-cells (from each microinjection group) that do not exhibit nuclear accumulated YAP, as opposed to outer-cell populations. In panels b) and c) yellow asterisk denotes the existence of phenotypically characteristic micronuclei in the *Wwc2* siRNA microinjected embryo groups. Additionally, the number of assayed embryos per group is indicated and the scale bar equals 20μm.

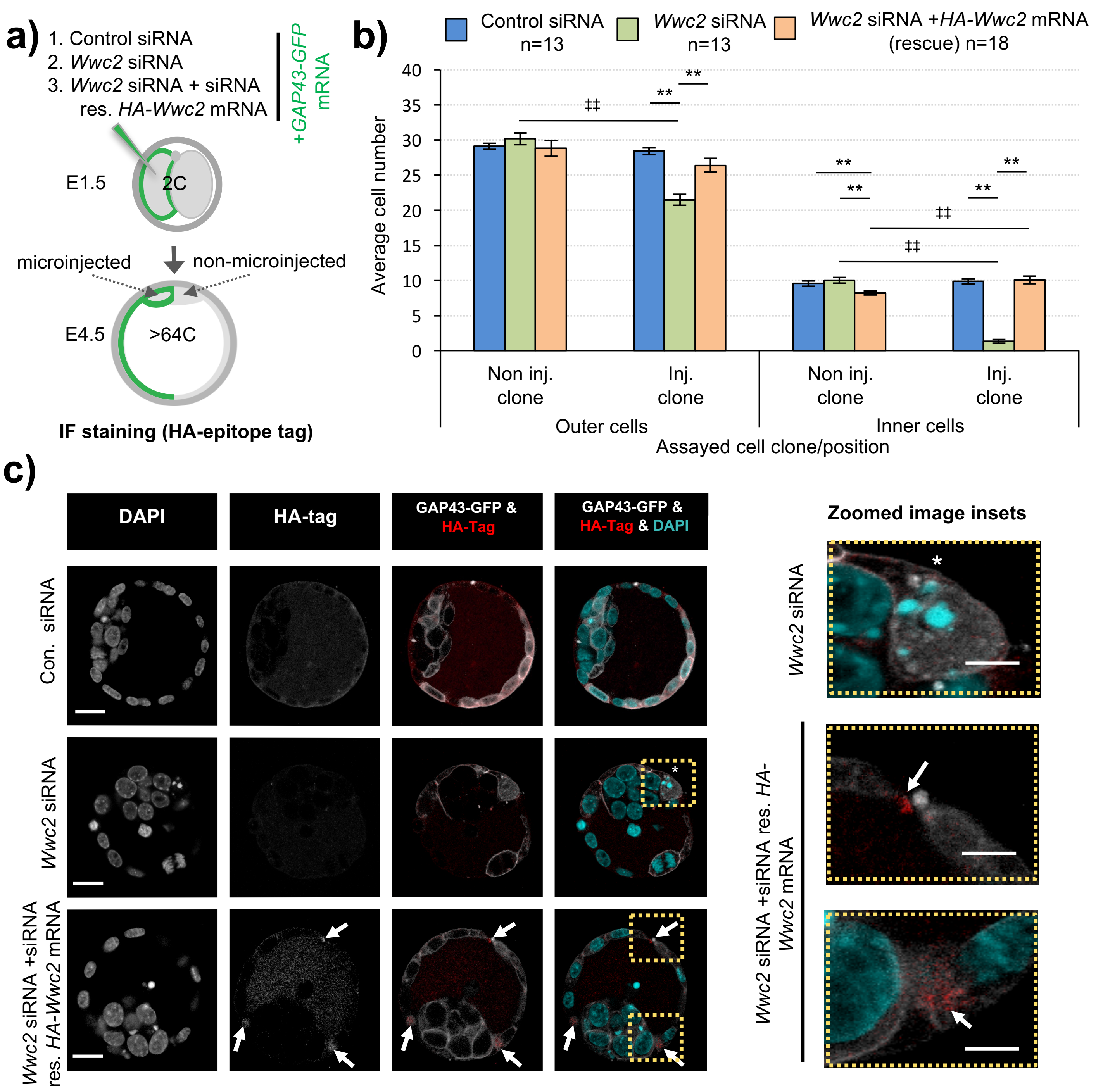
a)
1. Control siRNA
2. Wwc2 siRNA
3. Wwc2 siRNA + siRNA res. HA-Wwc2 mRNA
+GAP43-GFP mRNA
E1.5
2C
microinjected
non-microinjected
E4.5
>64C
IF staining (HA-epitope tag)
b)
Control siRNA n=13
Wwc2 siRNA n=13
Wwc2 siRNA +HA-Wwc2 mRNA (rescue) n=18
Average cell number
Non inj. clone
Inj. clone
Outer cells
Inner cells
Assayed cell clone/position
c)
DAPI
HA-tag
GAP43-GFP & HA-Tag
GAP43-GFP & HA-Tag & DAPI
Con. siRNA
Wwc2 siRNA
Wwc2 siRNA +siRNA res. HA-Wwc2 mRNA
Zoomed image insets
Wwc2 siRNA
Wwc2 siRNA +siRNA res. HA-Wwc2 mRNA

Fig. S12

Supplementary figure S12: Expression of siRNA resistant *HA-Wwc2* mRNA rescues *Wwc2* siRNA cell number/division phenotypes up to the late blastocyst/E4.5 (>64 cell) stage. a) Experimental strategy for potential phenotypic rescue of clonal *Wwc2* KD by comparing co-microinjection (in one blastomere of 2-cell stage embryos) of GAP43-GFP mRNA (stable plasma membrane fluorescent microinjection marker – Inj., to distinguish from the non-microinjected clone – Non inj.) with control siRNA, *Wwc2* siRNA or *Wwc2* siRNA + recombinant *HA-Wwc2* mRNA (containing N-terminal HA-epitope tag and point mutants conferring siRNA resistance – see Fig. S8) and assaying total, outer and inner-cell number at the >64-cell late blastocyst stage. **b)** Average clonal contribution (microinjected/Inj. versus non-microinjected/Non inj.) of outer and ICM/inner cells in control siRNA (blue bars), *Wwc2* siRNA (green bars) and *Wwc2* siRNA + *HA-Wwc2* mRNA (rescue – orange bars) microinjected embryos; errors represent s.e.m. and 2-tailed student t-test derived statistical significance between the experimental groups (asterisks), or clones (microinjected/Inj. versus non-microinjected/Non inj.) within a group (double crosses) highlighted with statistical confidence intervals of $p<0.05$ and $p<0.005$ (denoted by one or two significance markers, respectively). The number of embryos in each experimental group is provided. **c)** Exemplar confocal z-section micrographs of 32-cell blastocyst stage embryos, derived from the indicated microinjection groups, IF stained to detect HA-epitope tag expression (individual greyscale channels, plus merged images were the anti-HA channel is red and the DAPI cyan). Within the microinjected clone of the *Wwc2* siRNA treated embryo the white asterisk denotes characteristic apoptosis and fragmented nuclei, whilst in the microinjected clone of the *Wwc2* siRNA + *HA-Wwc2* rescue embryo example, the white arrows indicates anti-HA epitope tag IF signal associated with mid-bodies (see also indicated zoomed images - right) – note the association is not as pronounced as at the 32-cell blastocyst stage (Fig. 6); scale bar equals 20μm. Supplementary tables ST25 summarise statistical analysis and individual embryo data used to generate the figure.

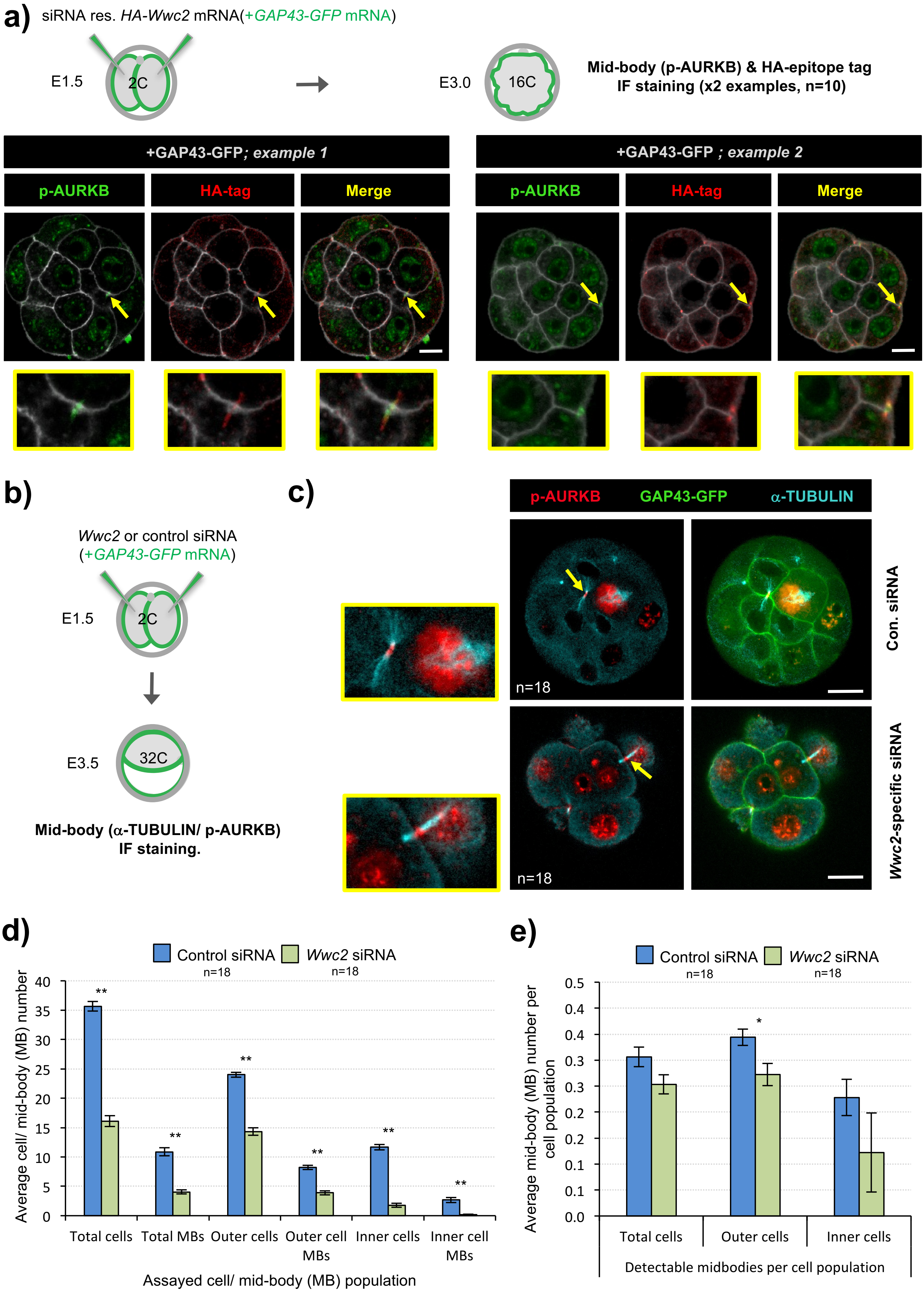
a)
siRNA res. HA-Wwc2 mRNA(+GAP43-GFP mRNA)
E1.5
2C
E3.0
16C
Mid-body (p-AURKB) & HA-epitope tag
IF staining (x2 examples, n=10)
+GAP43-GFP; example 1
+GAP43-GFP ; example 2
p-AURKB
HA-tag
Merge
b)
Wwc2 or control siRNA
(+GAP43-GFP mRNA)
E1.5
2C
E3.5
32C
Mid-body (α-TUBULIN/ p-AURKB)
IF staining.
c)
p-AURKB
GAP43-GFP
α-TUBULIN
n=18
Con. siRNA
Wwc2-specific siRNA
d)
Control siRNA
Wwc2 siRNA
n=18
Average cell/ mid-body (MB) number
Total cells
Total MBs
Outer cells
Outer cell MBs
Inner cells
Inner cell MBs
Assayed cell/ mid-body (MB) population
e)
Average mid-body (MB) number per cell population
Detectable midbodies per cell population

Fig. S13

Supplementary figure S13: Global *Wwc2* KD does not significantly alter the number of detectable mid-bodies by the 32-cell stage. a) Experimental approach to assay co-localisation of recombinant siRNA resistant HA-epitope tagged WWC2 protein with the mitotic mid-body marker phospho-Aurora kinase B (p-AURKB) at the 16-cell stage (upper). Briefly, recovered 2-cell stage embryos were co-microinjected in both blastomeres with recombinant mRNAs encoding siRNA resistant HA-WWC2 and GAP43-GFP (injection marker), cultured to the 16-cell stage and IF stained with antibodies against p-AURKB and HA-epitope tag. Exemplar micrographs (selected single z-sections) of two assayed embryos (lower panels) detail p-AURKB (green), HA-epitope (red) and merged channels; yellow arrows highlight mid-body structures, with magnified images of the regions of interest provided in the lower insets (note, localisation of HA-WWC2 on mid-body proximal microtubules that does not perfectly co-localise with the *de facto* mid-body marker, pAURKB). Scale bar equals 20 μm. **b)** Experimental strategy to assay the number of mid-bodies in *Wwc2* KD embryos by co-microinjecting *Wwc2* siRNA (or control siRNA) and GAP43-GFP mRNA (stable plasma membrane fluorescent injection marker) in both cells at the 2-cell stage, fixing after culture to the 32-cell stage and IF staining for the mid-body marker proteins α-TUBULIN and p-AURKB. **c)** Exemplar confocal z-section micrographs of composite IF stained 32-cell stage embryos; p-AURKB (red), α-TUBULIN (cyan) and GAP43-GFP (injection marker – green). Yellow arrows denote persistent mid-bodies characteristic of cleavage stage mouse embryos (zoomed images provided; yellow boxes – left). Number of embryos in each microinjection group provided; scale bars equal 20 μm). **d)** Average number of cells and detectable mid-bodies (in total or either outer or inner-cell populations; note, inner-cell populations defined by mid-body association with at least one inner-cell) per 32-cell stage embryo after microinjection of control (blue bars) or *Wwc2* (green bars) siRNA. **e)** The average number of mid-bodies observed in each stated microinjection group and cell type category, as normalised to the average cell number for that category (*i.e.* total, outer and inner-cells). In panels d) and e) chart error bars represent s.e.m. and 2-tailed student t-test derived statistical significance between the control and *Wwc2* knockdown groups indicated (** $p<0.005$). Supplementary tables new ST26 summarise statistical analysis and individual embryo data in each experimental group.

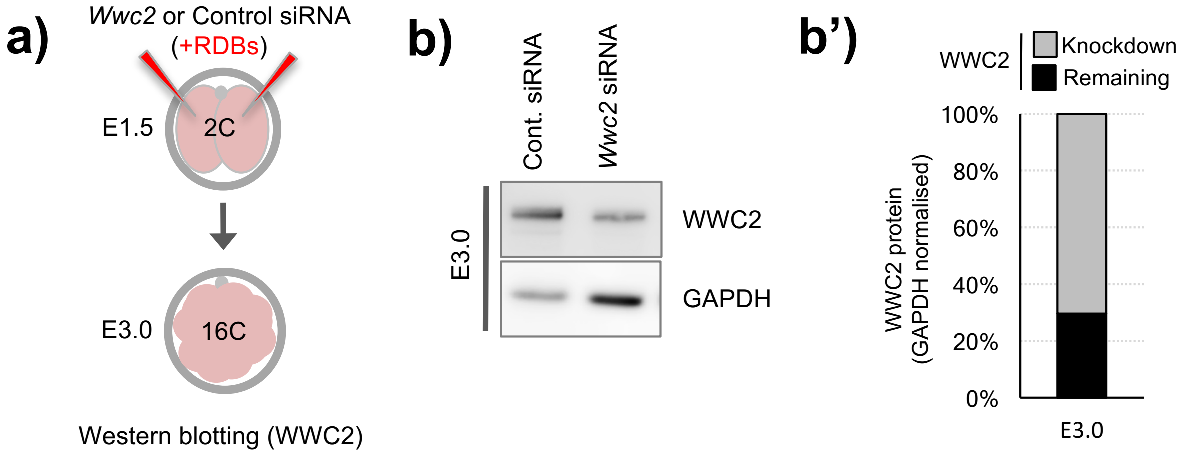
a)
Wwc2 or Control siRNA
(+RDBs)
E1.5
2C
E3.0
16C
Western blotting (WWC2)
b)
Cont. siRNA
Wwc2 siRNA
E3.0
WWC2
GAPDH
b')
WWC2
Knockdown
Remaining
100%
80%
60%
40%
20%
0%
WWC2 protein
(GAPDH normalised)
E3.0

Fig. S14

Supplementary figure S14: WWC2 protein expression in 16-cell mouse embryos after global siRNA mediated knockdown. a) Experimental scheme by which both blastomeres of 2-cell stage embryos were microinjected with either *Wwc2*-specific or control siRNA (plus RDBs as a microinjection marker) and *in vitro* cultured until the 16-cell stage before being processed for western blotting to assay normalised WWC2 protein expression. **b)** Western blots showing WWC2 expression (upper panel) and control GAPDH expression (lower panel) in control and *Wwc2*-specific siRNA microinjected embryos at the 16-cell stage. **b')** Quantification of the extent of *Wwc2*-specific siRNA induced WWC2 protein expression knockdown, from data show in panel b). The black bar denotes the amount of GAPDH normalised WWC2 protein expression remaining, versus the control condition and the grey bar the extent/efficiency of the knockdown.

Human & Mouse KIBRA & WWC2 protein sequence alignment; consensus AURK phosphorylation motifs

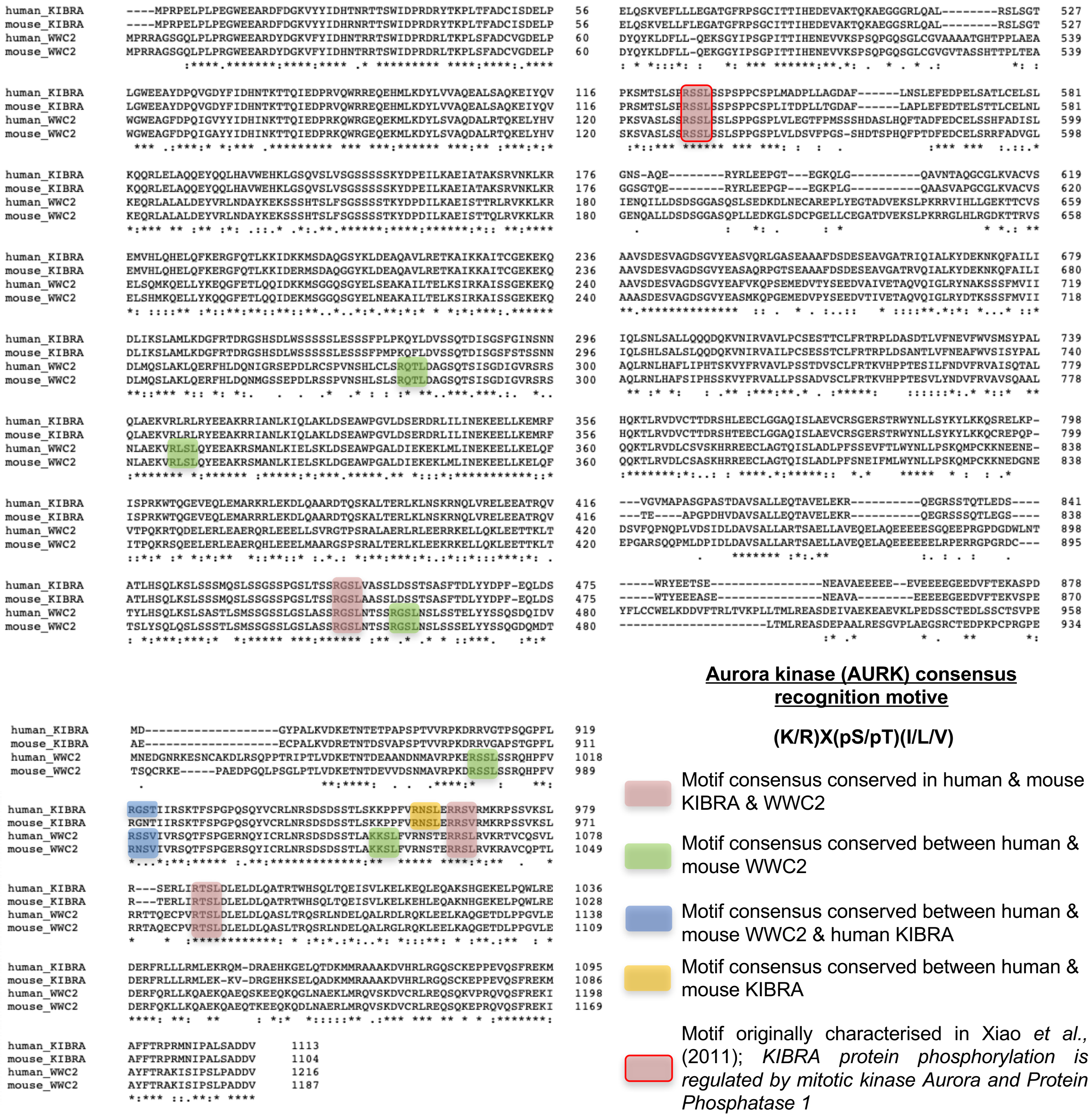

Fig. S15

Supplementary figure S15: Primary amino acid sequence alignment of human and mouse KIBRA and WWC2 proteins; with highlighted Aurora kinase consensus phosphorylation motifs. Amino acid sequence alignments are shown and sequences conforming to the Aurora kinase (AURK) consensus motif [(K/R)X(pS/pT)(I/L/V)] – where pS/pT denote the residue targeted for phosphorylation, highlighted according to the legend provided. All alignments were conducted using default setting of the online Clustal-Ω multiple sequence alignment tool; asterisks (*) denote perfectly aligned and identical amino acids, whereas colons (:) detail aligned conservative amino acid substitutions.

B) SUPPLEMENTARY METHODS TABLES: SM1 – SM6

***Wwc2* is a novel cell division regulator during preimplantation mouse embryo lineage formation and oogenesis.**

Virnicchi *et al.,* 2020

Supplementary Methods Tables SM1

**Microinjected RNAi constructs (Virnicchi *et al.,* 2020)**				
**Type**	**Gene Target**	**Souce (supplier)**	**Cat. No.**	**Microinjection conc.**
dsRNA	*Kibra*	in house synthesised	n/a	200 ng/μl
dsRNA	*Wwc2*	in house synthesised	n/a	200 ng/μl
dsRNA	*EGFP*	in house synthesised	n/a	200 ng/μl
siRNA	mouse genome neg. control	Qiagen (Murine Allstars neg. control siRNA)	SI03650318	10 μM
siRNA	*Kibra*	ThermoFisher Scientific (Silencer Select)	4390771: s102487	10 μM
siRNA	*Wwc2*	ThermoFisher Scientific (Silencer Select)	4390771: s78812	10 μM

**Microinjected recombinant & IVT derived mRNA constructs (Virnicchi *et al.,* 2020)**				
**Type**	**Gene Target**	**Souce (supplier)**	**Cat. No.**	**Microinjection conc.**
mRNA	*GAP43-GFP*	in house synthesised	n/a	60 ng/μl
mRNA	*H2B-RFP*	in house synthesised	n/a	40 ng/μl
mRNA	*Wwc2-HA (siRNA res.)*	in house synthesised	n/a	85 ng/μl

Supplementary Methods Table SM2

Oligonucleotide PCR primer sequence for dsRNA template generation (Virnicchi *et al.,* 2020)		
**dsRNA gene target**	**T7 RNA polymerase promoter sequence (lowever case) linked gene specific primer senquence**	
	**Sense (5'-3')**	**Anti-sense (5'-3')**
Kibra	taatacgactcactatagggGACTTCGACGGCAAGGTCTA	taatacgactcactatagggTCCGACCTGTGGGTCGTAT
Wwc2	taatacgactcactatagggGAGCTGCTGTGTGAGGGC	taatacgactcactatagggAGAAGGCATGAAGATTTCGG
Egfp	taatacgactcactatagggAGAGTACAAATTTTCTGTCAGTGGAGAGG	taatacgactcactatagggAGATGTATAGTTCATCCATGCCATGTGTA

Supplementary Methods Table SM3

Recombinant plasmid derivation information (Virnicchi *et al.,* 2020)					
**Plasmid name**	**Backbone vector**	**Insert cDNA(species derived from)**	**PCR oligonucleotide primer pair sequences (used for insert generation; *if applicable*) + introduced restriction enzyme recognition sites for sub cloning (lower case & underlined)**		**Notes**
			**Sense (5'-3') - introduced KOZAK & HA-epitope tag sequences highlighted**	**Anti-sense (5'-3')**	
pRN3-C-term-RFP-Hist1h2bb	pRN3-insert-RFP	Hist1h2bb, histonme H2B (mouse)	GACTATgctagcCCAGAGCCTTCTAAGTCTGCAC	GACTATgctagcCTTGGAGCTGGTGTACTTGGTGA	insert generating PCR oligos lack 'start' and 'stop' codons, as these are encoded in the vector (allowing N & C-terminal RFP fusions)
pGEM-T-Easy-N-term-HA-wildtype-Wwc2	pGEM®-T-Easy	Wwc2, wildtype (mouse)	GACTATactagtGCCACCATGggctacccatacgatgttcctgactatgctCCTAGGAGGGCCGGGAGC (*SpeI*)	GACTATgcggccgcTCACACGTCGTCAGCGGGC (*NotI*)	Insert generated by PCR and 'TA' cloned into pGEM®-T-Easy (and sequence verified)
pGEM-T-Easy-N-term-HA-siRNAres-Wwc2	pGEM®-T-Easy	Wwc2, siRNA resistant (mouse)	**n/a (**Wwc2 siRNA recognition sequence mutated *in situ* by SDM; commercial serive - EuorFins)		siRNA resistant mutant derived by commercial SDM (siRNA recognition motif mutated, codon info retained - see Fig. S8)

Supplementary Methods Table SM4

Q-RT-PCR Oligonucleotide primer sequences (Virnicchi *et al.,* 2020)		
**Target gene mRNA/ cDNA**	**Q-RT-PCR oligonucleotide primer sequences**	
	**Sense (5'-3')**	**Anti-sense (5'-3')**
Tbp (TATA-binding protein)	GAAGAACAATCCAGACTAGCAGCA	CCTTATAGGGAACTTCACATCACAG
Kibra	ATGATGAGAGCTGCTGCCAAGG	ATCCGAGGCCGGGTGAAAAATG
Wwc2	TGCTGGAGGACGAGAGATTC	GAGACCTGCCTCATCAACCT

Supplementary Methods Tables SM5

Primary antibody (immuno-fluorescence staining) information (Virnicchi *et al.,* 2020)						
**#**	**Antigen**	**cat. no.**	**Supplier**	**Species raised in & clonicity**	**Dilution used**	**Secondary antibody combination used (see below)**
1	CDH1	3195	Cell Signalling Technology	rabbit, polyclonal	1 in 500	E
2	CDX2	MU392A-UC	BioGenex	mouse, monoclonal	1 in 200	D
3	GATA4	sc-9053	Santa Cruz	rabbit, polyclonal	1 in 100	E
4	HA-TAG	ab9134	Abcam	goat, polyclonal	1 in 200	H
5	NANOG	14-5761	Affymetrix/eBioscience	rat, monoclonal	1 in 100	B
6	PARD6B	sc-67393	Santa Cruz	rabbit, polyclonal	1 in100	G
7	phospho-AURKA (Thr288)	NB100-2371	Novus Biologicals	rabbit, polyclonal	1 in 200	E
8	phospho-AURKB (Thr232)	600-401-677S	Rockland Antibodies	rabbit, polyclonal	1 in 200	F
9	SOX2	sc-365823	Santa Cruz	mouse, monoclonal	1 in 200	D
10	TUBULIN (alpha)	A11126	ThermoFisher Scientific	mouse, monoclonal	1 in 200	A/C
11	YAP1	sc-101199	Santa Cruz	mouse, monoclonal	1 in100	A

Secondary antibody (immuno-fluorescence staining) information (Virnicchi *et al.,* 2020)						
**#**	**Species of antibody targetted**	**cat. no.**	**Supplier**	**Species raised in & fluorophore**	**Dilution used**	**Primary antibody combination used (see above)**
A	mouse	715-605-150	Jackson Immuno Research Inc.	donkey, Alexa647	1 in 500	10/11
B	rat	715-096-150	Jackson Immuno Research Inc.	donkey, FITC	1 in 400	5
C	mouse	A-21202	ThermoFisher Scientific	donkey Alexa488	1 in 500	10
D	mouse	A-31570	ThermoFisher Scientific	donkey Alexa555	1 in 500	2/9
E	rabbit	A-21206	ThermoFisher Scientific	donkey, Alexa488	1 in 500	1/3/7
F	rabbit	A-31572	ThermoFisher Scientific	donkey Alexa555	1 in 500	8/9
G	rabbit	A-31573	ThermoFisher Scientific	donkey, Alexa647	1 in 500	6
H	goat	A-21432	ThermoFisher Scientific	donkey, Alexa555	1 in 500	4

Supplementary Methods Tables SM6

Primary antibody (western blot) information (Virnicchi *et al.,* 2020)						
**#**	**Antigen**	**cat. no.**	**Supplier**	**Species raised in & clonicity**	**Dilution used**	**Secondary antibody combination used (see below)**
1	phospho-AURKA (Thr288)	2914	Cell Signalling Technology	rabbit, monoclonal	1 in 2,000	A
2	WWC2	ab126356	Abcam	rabbit, polyclonal	1 in 500	A
3	GAPDH	G9545	Merck (Sigma-Aldrich)	rabbit, polyclonal	1 in 20,000	A
4	AURKA	4718	Cell Signalling Technology	rabbit, monoclonal	1 in 100	A

Secondary HRP-CONJUGATED antibody (western blot) information (Virnicchi *et al.,* 2020)						
**#**	**Species of antibody targetted**	**cat. no.**	**Supplier**	**Species raised in**	**Dilution used**	**Primary antibody combination used (see above)**
A	rabbit	711-035-152	Jackson Immuno Research Inc.	donkey, polyclonal	1 in 10,000	1/2/3/4

C) **SUPPLEMENTARY TABLES (individual embryo cell counts & oocyte phenotypes + statistics): ST1 – ST26**

***Wwc2* is a novel cell division regulator during preimplantation mouse embryo lineage formation and oogenesis.**

Virnicchi *et al.,* 2020

Supplementary Tables ST1

Control siRNA (10μM), 2in2 micro-injection, 4-cell (E2.0) stage					
#	Total cell number per embryo	Total number of dividing cells	Number of cells exhibiting an atypical/ unusual phenotype morphology		
			Abnormal nuclear morphology (e.g. non-spheroidal or mid-body associated)	Defective cytokinesis (i.e. >x2 equally sized nuclei per cell)	Presence of micronuclei
**1**	4	0	0	0	0
**2**	4	0	0	0	0
**3**	4	0	0	0	0
**4**	4	0	0	0	0
**5**	4	0	0	0	0
**6**	4	0	0	0	0
**7**	4	0	0	0	0
**8**	4	0	0	0	0
**9**	3	1	0	0	0
**10**	4	0	0	0	0
**11**	4	0	0	0	0
**12**	4	0	0	0	0
**13**	4	0	0	0	0
**TOTAL**	**51**	**1**	**0**	**0**	**0**
**AVERAGE**	**3.9**	**0.1**	**0.0**	**0.0**	**0.0**
SEM	**0.1**	**0.08**	**0.0**	**0.0**	**0.0**

Wwc2-specific siRNA (10μM), 2in2 micro-injection, 4-cell (E2.0) stage					
#	Total cell number per embryo	Total number of dividing cells	Number of cells exhibiting an atypical/ unusual phenotype morphology		
			Abnormal nuclear morphology (e.g. non-spheroidal or mid-body associated)	Defective cytokinesis (i.e. >x2 equally sized nuclei per cell)	Presence of micronuclei
**1**	4	0	0	0	0
**2**	4	0	0	0	0
**3**	4	0	0	0	0
**4**	4	0	0	0	0
**5**	4	0	0	0	0
**6**	3	1	0	0	0
**7**	4	0	0	0	0
**8**	4	0	0	0	0
**9**	4	0	0	0	0
**10**	4	0	0	0	0
**11**	4	0	0	0	0
**12**	4	0	0	0	0
**13**	4	0	0	0	0
**14**	4	0	0	0	0
**TOTAL**	**55**	**1**	**0**	**0**	**0**
**AVERAGE**	**3.9**	**0.1**	**0.0**	**0.0**	**0.0**
SEM	**0.1**	**0.1**	**0.0**	**0.0**	**0.0**
Stat. sig. (exp. vs. con embryo) *p<0.05, **p<0.005					
p-value (2-tailed students t-test)	9.59E-01	9.59E-01	n/a	n/a	n/a

Supplementary Tables ST2

Control siRNA (10μM), 2in2 micro-injection, 8-cell (E2.5) stage					
#	Total cell number per embryo	Total number of dividing cells	Number of cells exhibiting an atypical/ unusual phenotype morphology		
			Abnormal nuclear morphology (e.g. non-spheroidal or mid-body associated)	Defective cytokinesis (i.e. >x2 equally sized nuclei per cell)	Presence of micronuclei
**1**	6	0	0	0	0
**2**	8	0	0	0	0
**3**	8	0	0	0	0
**4**	8	0	0	0	0
**5**	8	0	0	0	0
**6**	8	0	0	0	0
**TOTAL**	**46**	**0**	**0**	**0**	**0**
**AVERAGE**	**7.7**	**0.0**	**0.0**	**0.0**	**0.0**
SEM	**0.3**	**0.0**	**0.0**	**0.0**	**0.0**

Wwc2-specific siRNA (10μM), 2in2 micro-injection, 8-cell (E2.5) stage					
#	Total cell number per embryo	Total number of dividing cells	Number of cells exhibiting an atypical/ unusual phenotype morphology		
			Abnormal nuclear morphology (e.g. non-spheroidal or mid-body associated)	Defective cytokinesis (i.e. >x2 equally sized nuclei per cell)	Presence of micronuclei
**1**	7	1	2	0	0
**2**	7	0	0	0	0
**3**	6	0	0	0	0
**4**	8	0	0	0	0
**5**	8	0	3	0	0
**6**	8	0	0	0	0
**7**	8	0	0	0	0
**TOTAL**	**52**	**1**	**5**	**0**	**0**
**AVERAGE**	**7.4**	**0.1**	**0.7**	**0.0**	**0.0**
SEM	**0.3**	**0.1**	**0.5**	**0.0**	**0.0**
Stat. sig. (exp. vs. con embryo) *p<0.05, **p<0.005					
p-value (2-tailed students t-test)	6.04E-01	3.77E-01	1.93E-01	n/a	n/a

Supplementary Tables ST3

Control siRNA (10μM), 2in2 micro-injection, 16-cell (E3.0) stage

#	Total cell number per embryo	Total number of dividing cells	Number of cells exhibiting an atypical/ unusual phenotype morphology		
			Abnormal nuclear morphology (e.g. non-spheroidal or mid-body associated)	Defective cytokinesis (i.e. >x2 equally sized nuclei per cell)	Presence of micronuclei
**1**	15		0	0	0
**2**	16		0	0	0
**3**	14		0	0	0
**4**	16		0	0	0
**5**	16		0	0	0
**6**	16		0	0	0
**7**	19		0	0	0
**8**	16		0	0	0
**9**	16		0	0	0
**10**	17		0	0	0
**11**	16		0	0	0
**12**	19		0	0	0
**13**	19		0	0	0
**14**	16		0	0	0
**15**	16		0	0	0
**16**	19		0	0	0
**17**	15		0	0	0
**18**	18		0	0	0
**19**	17		0	0	0
**20**	16		0	0	0
**21**	17		0	0	0
**22**	27		0	0	0
**23**	16		0	0	0
**24**	19		0	0	0
**25**	31		0	0	0
**26**	16		0	0	0
**27**	16		0	0	0
**28**	14		0	0	0
**29**	16		0	0	0
**30**	15		0	0	0
**TOTAL**	**519**	**0**	**0**	**0**	**0**
**AVERAGE**	**17.3**	**0.0**	**0.0**	**0.0**	**0.0**
SEM	**0.6**	**0.00**	**0.0**	**0.0**	**0.0**

Wwc2-specific siRNA (10μM), 2in2 micro-injection, 16-cell (E3.0) stage

#	Total cell number per embryo	Total number of dividing cells	Number of cells exhibiting an atypical/ unusual phenotype morphology		
			Abnormal nuclear morphology (e.g. non-spheroidal or mid-body associated)	Defective cytokinesis (i.e. >x2 equally sized nuclei per cell)	Presence of micronuclei
**1**	8		0	0	0
**2**	9		1	0	0
**3**	8		1	0	0
**4**	8		0	1	0
**5**	8		0	0	3
**6**	8		0	0	0
**7**	8		0	0	1
**8**	10		0	0	0
**9**	9		2	0	1
**10**	8		0	0	0
**11**	9		1	1	0
**12**	8		1	0	0
**13**	8		1	0	1
**14**	10		0	0	0
**15**	14		0	0	0
**16**	8		0	0	0
**17**	8		0	1	1
**18**	8		2	0	1
**19**	8		2	0	0
**20**	12		2	1	2
**21**	6		1	0	0
**22**	10		1	1	1
**23**	5		1	0	0
**24**	5		0	0	2
**25**	10		1	0	2
**26**	7		0	0	1
**27**	14		1	1	3
**28**	10		2	1	2
**29**	8		1	0	0
**30**	8		1	0	0
**31**	8		0	0	0
**TOTAL**	**268**	**0**	**22**	**7**	**21**
**AVERAGE**	**8.6**	**0.0**	**0.7**	**0.2**	**0.7**
SEM	**0.4**	**0.0**	**0.1**	**0.1**	**0.2**
Stat. sig. (exp. vs. con embryo) *p<0.05, **p<0.005	**		**	*	**
p-value (2-tailed students t-test)	3.00E-17	0.00E+00	2.11E-06	5.10E-03	2.28E-04

Supplementary Tables ST4

Control siRNA (10μM), 2in2 micro-injection, 32-cell (E3.5) stage

#	Total cell number per embryo	Total number of dividing cells	Number of cells exhibiting an atypical/ unusual phenotype morphology		
			Abnormal nuclear morphology (e.g. non-spheroidal or mid-body associated)	Defective cytokinesis (i.e. >x2 equally sized nuclei per cell)	Presence of micronuclei
**1**	33		0	0	0
**2**	24		0	0	0
**3**	28		0	0	0
**4**	31		0	0	0
**5**	31		0	0	0
**6**	38		0	0	0
**7**	32		0	0	0
**8**	31		0	0	0
**9**	35		0	0	0
**10**	31		0	0	0
**11**	31		0	0	0
**12**	34		0	0	0
**13**	32		0	0	0
**14**	22		0	0	0
**15**	24		0	0	0
**16**	35		0	0	0
**17**	32		0	0	0
**18**	38		0	0	0
**19**	36		0	0	0
**20**	37		0	0	0
**21**	32		0	0	0
**22**	42		0	0	0
**23**	32		0	0	0
**24**	29		0	0	0
**25**	32		0	0	0
**26**	32		0	0	0
**27**	32		0	0	0
**28**	31		0	0	1
**29**	26		0	0	0
**30**	28		0	0	0
**31**	37		0	0	0
**32**	36		0	0	0
**33**	37		0	0	0
**34**	35		0	0	0
**35**	33		0	0	0
**36**	37		0	0	0
**37**	42		0	0	0
**38**	40		0	0	0
**39**	36		0	0	0
**40**	37		0	0	0
**41**	33		0	0	0
**42**	30		0	0	0
**43**	33		0	0	0
**44**	37		0	0	0
**45**	38		0	0	0
**46**	30		0	0	0
**47**	34		0	0	0
**48**	38		0	0	0
**49**	37		0	0	0
**TOTAL**	**1631**	**0**	**0**	**0**	**1**
**AVERAGE**	**33.3**	**0.0**	**0.0**	**0.0**	**0.0**
SEM	**0.6**	**0.0**	**0.0**	**0.0**	**0.0**

Kibra siRNA (10μM), 2in2 micro-injection, 32-cell (E3.5) stage

#	Total cell number per embryo	Total number of dividing cells	Number of cells exhibiting an atypical/ unusual phenotype morphology		
			Abnormal nuclear morphology (e.g. non-spheroidal or mid-body	Defective cytokinesis (i.e. >x2 equally sized nuclei per cell)	Presence of micronuclei
**1**	35		0	0	0
**2**	32		0	0	0
**3**	32		0	0	0
**4**	31		0	0	0
**5**	36		0	0	0
**6**	35		0	0	0
**7**	35		0	0	0
**8**	33		0	0	0
**9**	39		0	0	0
**10**	35		0	0	0
**11**	36		0	0	0
**12**	34		0	0	0
**13**	37		0	0	0
**14**	40		0	0	0
**15**	37		0	0	0
**16**	33		0	0	0
**17**	34		0	0	0
**18**	37		0	0	0
**19**	38		0	0	0
**20**	38		0	0	0
**21**	33		0	0	0
**22**	34		0	0	0
**23**	36		0	0	0
**TOTAL**	**810**	**0**	**0**	**0**	**0**
**AVERAGE**	**35.2**	**0.0**	**0.0**	**0.0**	**0.0**
SEM	**0.5**	**0.0**	**0.0**	**0.0**	**0.0**
Stat. sig. (exp. vs. con embryo) *p<0.05, **p<0.005					
p-value (2-tailed students t-test)	5.02E-02	0.00E+00	0.00E+00	0.00E+00	4.97E-01

Wwc2-specific siRNA (10μM), 2in2 micro-injection, 32-cell (E3.5) stage

#	Total cell number per embryo	Total number of dividing cells	Number of cells exhibiting an atypical/ unusual phenotype morphology		
			Abnormal nuclear morphology (e.g. non-spheroidal or mid-body associated)	Defective cytokinesis (i.e. >x2 equally sized nuclei per cell)	Presence of micronuclei
**1**	7		0	0	3
**2**	15		0	0	0
**3**	7		0	0	3
**4**	14		0	0	0
**5**	6		1	0	1
**6**	7		1	0	1
**7**	8		1	1	0
**8**	7		0	0	0
**9**	7		0	0	1
**10**	8		0	0	1
**11**	8		2	0	3
**12**	12		0	0	0
**13**	12		0	0	1
**14**	7		1	0	0
**15**	9		0	0	4
**16**	10		1	0	2
**17**	7		0	0	2
**18**	7		0	0	0
**19**	16		0	0	0
**20**	9		0	1	2
**21**	12		2	1	3
**22**	16		1	2	6
**23**	13		4	1	4
**24**	6		2	1	0
**25**	17		1	0	0
**26**	9		1	0	2
**27**	8		0	0	0
**28**	8		0	1	4
**29**	8		2	0	0
**30**	8		1	0	0
**31**	11		0	1	0
**32**	6		0	0	2
**33**	12		1	0	1
**34**	7		0	0	1
**35**	15		0	0	2
**36**	8		0	0	0
**TOTAL**	**347**	**0**	**22**	**9**	**49**
**AVERAGE**	**9.6**	**0.0**	**0.6**	**0.3**	**1.4**
SEM	**0.5**	**0.0**	**0.2**	**0.1**	**0.3**
Stat. sig. (exp. vs. con embryo) *p<0.05, **p<0.005	**		**	**	**
p-value (2-tailed students t-test)	2.23E-43	0.00E+00	8.54E-06	7.33E-04	4.46E-08
Stat. sig. (exp. vs. exp Kibra KD embryo) §p<0.05, §§p<0.005	§§	§§	§§	§	§§
p-value (2-tailed students t-test)	1.65E-38	0.00E+00	2.03E-03	2.02E-02	9.72E-05

Supplementary Tables ST5

Control siRNA (10μM), 2in2 micro-injection, ~64-cell (E4.0) stage					
#	Total cell number per embryo	Total number of dividing cells	Number of cells exhibiting an atypical/ unusual phenotype morphology		
			Abnormal nuclear morphology (e.g. non-spheroidal or mid-body associated)	Defective cytokinesis (i.e. >x2 equally sized nuclei per cell)	Presence of micronuclei
**1**	60	3	0	0	0
**2**	68	4	0	0	0
**3**	68	8	0	0	0
**4**	72	3	0	0	0
**5**	64	1	0	0	0
**6**	70	0	0	0	0
**7**	62	5	0	0	0
**8**	68	4	0	0	0
**9**	60	3	0	0	0
**10**	66	5	0	0	0
**11**	72	2	0	0	0
**12**	64	1	0	0	0
**13**	68	3	0	0	0
**14**	60	2	0	0	0
**15**	70	1	0	0	0
**16**	66	3	0	0	0
**TOTAL**	**1058**	**48**	**0**	**0**	**0**
**AVERAGE**	**66.1**	**3.0**	**0.0**	**0.0**	**0.0**
SEM	**1.0**	**0.5**	**0.0**	**0.0**	**0.0**

Wwc2-specific siRNA (10μM), 2in2 micro-injection, ~64-cell (E4.0) stage					
#	Total cell number per embryo	Total number of dividing cells	Number of cells exhibiting an atypical/ unusual phenotype morphology		
			Abnormal nuclear morphology (e.g. non-spheroidal or mid-body associated)	Defective cytokinesis (i.e. >x2 equally sized nuclei per cell)	Presence of micronuclei
**1**	34	0	2	0	7
**2**	24	1	3	0	9
**3**	22	2	1	0	4
**4**	24	2	2	0	3
**5**	18	0	3	0	2
**6**	21	0	4	0	3
**7**	20	0	3	0	3
**8**	29	1	2	0	8
**9**	16	0	4	0	4
**10**	18	0	2	0	4
**11**	23	0	1	0	5
**12**	16	0	4	0	8
**13**	18	3	2	0	4
**14**	22	2	3	0	6
**15**	16	0	4	2	3
**16**	23	1	2	0	3
**17**	20	0	4	0	7
**18**	24	0	3	0	5
**TOTAL**	**388**	**12**	**49**	**2**	**88**
**AVERAGE**	**21.6**	**0.7**	**2.7**	**0.1**	**4.9**
SEM	**1.1**	**0.2**	**0.2**	**0.1**	**0.5**
Stat. sig. (exp. vs. con embryo) *p<0.05, **p<0.005	**	**	**		**
p-value (2-tailed students t-test)	9.26E-25	9.32E-05	4.43E-12	3.54E-01	1.49E-10

Supplementary Tables ST6

Control siRNA (10µM), 2in2 micro-injection, >64-cell (E4.5) stage

#	Total cell number per embryo	Total number of dividing cells	Number of cells exhibiting an atypical/ unusual phenotype morphology		
			Abnormal nuclear morphology (e.g. non-spheroidal or mid-body associated)	Defective cytokinesis (i.e. >x2 equally sized nuclei per cell)	Presence of micronuclei
**1**	81	4	0	0	0
**2**	76	2	0	0	0
**3**	67	8	0	0	0
**4**	77	4	0	0	0
**5**	110	5	0	0	0
**6**	107	6	0	0	0
**7**	76	5	0	0	0
**8**	94	4	0	0	0
**9**	102	1	0	0	0
**10**	108	7	0	0	0
**11**	85	5	0	0	0
**12**	73	4	0	0	0
**13**	82	7	0	0	0
**14**	80	5	0	0	0
**TOTAL**	**1218**	**67**	**0**	**0**	**0**
**AVERAGE**	**87.0**	**4.8**	**0.0**	**0.0**	**0.0**
SEM	**3.8**	**0.5**	**0.0**	**0.0**	**0.0**

Wwc2-specific siRNA (10µM), 2in2 micro-injection, >64-cell (E4.5) stage

#	Total cell number per embryo	Total number of dividing cells	Number of cells exhibiting an atypical/ unusual phenotype morphology		
			Abnormal nuclear morphology (e.g. non-spheroidal or mid-body associated)	Defective cytokinesis (i.e. >x2 equally sized nuclei per cell)	Presence of micronuclei
**1**	13	0	6	1	6
**2**	57	7	7	1	5
**3**	31	3	5	0	7
**4**	13	1	2	0	2
**5**	21	6	4	0	5
**6**	28	2	4	0	4
**7**	30	3	5	0	3
**8**	28	2	4	0	3
**9**	36	1	3	1	6
**10**	26	5	2	0	2
**11**	51	5	4	0	4
**12**	35	3	2	0	7
**13**	45	3	5	0	3
**14**	32	3	3	0	4
**15**	44	4	4	0	4
**16**	19	3	3	0	2
**17**	23	5	2	1	3
**TOTAL**	**532**	**56**	**65**	**4**	**70**
**AVERAGE**	**31.3**	**3.3**	**3.8**	**0.2**	**4.1**
SEM	**3.0**	**0.5**	**0.4**	**0.1**	**0.4**
Stat. sig. (exp. vs. con embryo) *p<0.05, **p<0.005	**	*	**		**
p-value (2-tailed students t-test)	2.27E-12	3.55E-02	1.26E-10	5.41E-02	3.44E-10

Supplementary Tables ST7

Control siRNA (10µM), 1in2 micro-injection, 4-cell (E2.0) stage						
#	Total cell number per embryo	Cells in injected clone	Cells in non-injected clone	Dividing cells in injected clone	Dividing cells in non-injected clone	Total number of dividing cells
**1**	4	2	2	0	0	0
**2**	4	2	2	0	0	0
**3**	6	3	3	0	1	1
**4**	5	3	2	0	1	1
**5**	4	2	2	0	0	0
**6**	4	2	2	1	1	2
**7**	5	2	3	1	0	1
**8**	4	2	2	1	1	2
**9**	6	3	3	1	1	2
**10**	4	2	2	0	0	0
**11**	5	2	3	1	0	1
**12**	4	2	2	0	0	0
**TOTAL**	**55**	**27**	**28**	**5**	**5**	**10**
**AVERAGE**	**4.6**	**2.3**	**2.3**	**0.4**	**0.4**	**0.8**
SEM	**0.2**	**0.1**	**0.1**	**0.1**	**0.1**	**0.2**
Stat. sig. (inter-clone) ‡p<0.05, ‡‡p<0.005						
p-value (2-tailed students t-test)		6.70E-01		1.00E+00		

Wwc2-specific siRNA (10µM), 1in2 micro-injection, 4-cell (E2.0) stage						
#	Total cell number per embryo	Cells in injected clone	Cells in non-injected clone	Dividing cells in injected clone	Dividing cells in non-injected clone	Total number of dividing cells
**1**	4	2	2	0	1	1
**2**	4	2	2	0	0	0
**3**	5	3	2	0	1	1
**4**	4	2	2	0	0	0
**5**	4	2	2	0	1	1
**6**	4	2	2	0	0	0
**7**	5	2	3	0	1	1
**8**	4	2	2	0	1	1
**9**	4	2	2	0	0	0
**10**	4	2	2	0	0	0
**11**	5	2	3	0	0	0
**12**	4	2	2	0	0	0
**13**	4	2	2	0	1	1
**14**	5	2	3	0	0	0
**15**	4	2	2	0	1	1
**TOTAL**	**64**	**31**	**33**	**0**	**7**	**7**
**AVERAGE**	**4.3**	**2.1**	**2.2**	**0.0**	**0.5**	**0.5**
SEM	**0.1**	**0.1**	**0.1**	**0.0**	**0.1**	**0.1**
Stat. sig. (inter-clone) ‡p<0.05, ‡‡p<0.005				**‡‡**		
p-value (2-tailed students t-test)		2.99E-01		1.58E-03		
Stat. sig. (exp. vs. con embryo) *p<0.05, **p<0.005				**		
p-value (2-tailed students t-test)	2.05E-01	1.97E-01	4.52E-01	4.20E-03	8.04E-01	1.73E-01

Supplementary Tables ST8

Control siRNA (10μM), 1in2 micro-injection, 8-cell (E2.5) stage						
#	Total cell number per embryo	Cells in injected clone	Cells in non-injected clone	Dividing cells in injected clone	Dividing cells in non-injected clone	Total number of dividing cells
**1**	8	4	4	0	0	0
**2**	8	4	4	0	1	1
**3**	8	4	4	0	1	1
**4**	9	4	5	0	0	0
**5**	8	4	4	0	0	0
**6**	8	4	4	1	0	1
**7**	8	4	4	0	0	0
**8**	7	4	4	0	0	0
**9**	8	4	4	0	1	1
**10**	9	4	5	1	0	1
**11**	8	4	4	0	1	1
**12**	9	4	5	0	0	0
**13**	8	4	4	1	0	1
**TOTAL**	**106**	**52**	**55**	**3**	**4**	**7**
**AVERAGE**	**8.2**	**4.0**	**4.2**	**0.2**	**0.3**	**0.5**
SEM	**0.2**	**0.0**	**0.1**	**0.1**	**0.1**	**0.1**
Stat. sig. (inter-clone) ‡p<0.05, ‡‡p<0.005						
p-value (2-tailed students t-test)		6.99E-02		6.74E-01		

Wwc2-specific siRNA (10μM), 1in2 micro-injection, 8-cell (E2.5) stage						
#	Total cell number per embryo	Cells in injected clone	Cells in non-injected clone	Dividing cells in injected clone	Dividing cells in non-injected clone	Total number of dividing cells
**1**	6	2	4	0	2	2
**2**	5	2	3	0	1	1
**3**	6	2	4	0	0	0
**4**	6	2	4	0	0	0
**5**	7	3	4	0	0	0
**6**	7	3	4	0	1	1
**7**	6	2	4	1	1	2
**8**	6	2	4	0	1	1
**9**	6	2	4	0	0	0
**10**	8	4	4	0	1	1
**11**	6	2	4	0	0	0
**12**	7	2	5	0	0	0
**13**	5	2	3	0	1	1
**14**	6	2	4	0	0	0
**15**	6	2	4	0	0	0
**16**	6	2	4	1	2	3
**17**	6	2	4	0	0	0
**18**	6	2	4	0	0	0
**19**	6	2	4	0	1	1
**TOTAL**	**117**	**42**	**75**	**2**	**11**	**13**
**AVERAGE**	**6.2**	**2.2**	**3.9**	**0.1**	**0.6**	**0.7**
SEM	**0.2**	**0.1**	**0.1**	**0.1**	**0.2**	**0.6**
Stat. sig. (inter-clone) ‡p<0.05, ‡‡p<0.005		‡‡		‡		
p-value (2-tailed students t-test)		2.25E-13		1.01E-02		
Stat. sig. (exp. vs. con embryo) *p<0.05, **p<0.005	**	**				
p-value (2-tailed students t-test)	1.08E-09	5.70E-13	6.97E-02	3.53E-01	2.31E-01	5.98E-01

Supplementary Tables ST9

Control siRNA (10μM), 1in2 micro-injection, 16-cell (E3.0) stage						
#	Total cell number per embryo	Cells in injected clone	Cells in non-injected clone	Dividing cells in injected clone	Dividing cells in non-injected clone	Total number of dividing cells
**1**	16	8	8	0	0	0
**2**	16	8	8	0	0	0
**3**	16	8	8	0	0	0
**4**	15	7	8	0	2	2
**5**	17	8	9	1	0	1
**6**	16	8	8	0	3	3
**7**	16	8	8	0	0	0
**8**	15	8	7	0	0	0
**9**	15	7	8	0	0	0
**10**	16	8	8	0	0	0
**11**	15	7	8	0	0	0
**12**	16	8	8	1	0	1
**13**	15	8	7	1	1	2
**TOTAL**	**204**	**101**	**103**	**3**	**6**	**9**
**AVERAGE**	**15.7**	**7.8**	**7.9**	**0.2**	**0.5**	**0.7**
SEM	**0.2**	**0.1**	**0.1**	**0.088**	**0.300**	**0.3**
Stat. sig. (inter-clone) ‡p<0.05, ‡‡p<0.005						
p-value (2-tailed students t-test)		4.09E-01		4.41E-01		

Wwc2-specific siRNA (10μM), 1in2 micro-injection, 16-cell (E3.0) stage						
#	Total cell number per embryo	Cells in injected clone	Cells in non-injected clone	Dividing cells in injected clone	Dividing cells in non-injected clone	Total number of dividing cells
**1**	11	4	7	0	1	1
**2**	13	5	8	1	0	1
**3**	12	4	8	0	0	0
**4**	12	4	8	0	0	0
**5**	13	5	8	0	1	1
**6**	12	3	9	0	1	1
**7**	12	4	8	0	0	0
**8**	12	4	8	0	0	0
**9**	12	4	8	1	0	1
**10**	11	4	7	0	1	1
**11**	12	4	8	0	1	1
**12**	12	4	8	0	0	0
**13**	11	4	7	0	0	0
**14**	14	6	8	1	0	1
**15**	12	4	8	0	0	0
**16**	14	4	10	0	1	1
**17**	11	3	8	0	0	0
**18**	12	4	8	1	1	2
**TOTAL**	**218**	**74**	**144**	**4**	**7**	**11**
**AVERAGE**	**12.1**	**4.1**	**8.0**	**0.2**	**0.4**	**0.6**
SEM	**0.2**	**0.2**	**0.2**	**0.101**	**0.118**	**0.1**
Stat. sig. (inter-clone) ‡p<0.05, ‡‡p<0.005		‡‡				
p-value (2-tailed students t-test)		2.73E-18		2.91E-01		
Stat. sig. (exp. vs. con embryo) *p<0.05, **p<0.005	**	**				
p-value (2-tailed students t-test)	4.93E-13	1.19E-16	7.33E-01	9.57E-01	7.87E-01	7.85E-01

Supplementary Tables ST10

Control siRNA (10μM), 1in2 micro-injection, 32-cell (E3.5) stage						
#	Total cell number per embryo	Cells in injected clone	Cells in non-injected clone	Dividing cells in injected clone	Dividing cells in non-injected clone	Total number of dividing cells
**1**	43	22	21	3	6	9
**2**	31	16	15	0	1	1
**3**	33	16	17	0	1	1
**4**	35	17	18	6	3	9
**5**	35	16	19	3	1	4
**6**	32	16	16	0	0	0
**7**	31	15	16	1	0	1
**8**	33	16	17	0	0	0
**9**	31	16	15	0	1	1
**10**	34	16	18	0	0	0
**TOTAL**	**338**	**166**	**172**	**13**	**13**	**26**
**AVERAGE**	**33.8**	**16.6**	**17.2**	**1.3**	**1.3**	**2.6**
SEM	**1.1**	**0.6**	**0.6**	**0.7**	**0.6**	**1.1**
Stat. sig. (inter-clone) ‡p<0.05, ‡‡p<0.005						
p-value (2-tailed students t-test)		4.92E-01		1.00E+00		

Wwc2-specific siRNA (10μM), 1in2 micro-injection, 32-cell (E3.5) stage						
#	Total cell number per embryo	Cells in injected clone	Cells in non-injected clone	Dividing cells in injected clone	Dividing cells in non-injected clone	Total number of dividing cells
**1**	12	3	9	0	1	1
**2**	25	10	15	0	1	1
**3**	18	3	15	0	0	0
**4**	21	6	15	0	2	2
**5**	20	4	16	1	0	1
**6**	21	5	16	1	0	1
**7**	21	7	14	1	0	1
**8**	14	4	10	0	2	2
**9**	23	8	15	0	0	0
**10**	26	12	14	1	0	1
**11**	37	16	21	0	5	5
**12**	20	6	14	2	1	3
**13**	32	16	16	0	0	0
**14**	19	5	14	0	0	0
**15**	23	8	15	0	0	0
**16**	24	8	16	0	0	0
**17**	19	4	15	0	0	0
**18**	20	4	16	0	0	0
**19**	26	10	16	0	0	0
**20**	17	4	13	0	0	0
**21**	20	4	16	0	0	0
**22**	17	4	13	0	2	2
**23**	20	4	16	0	0	0
**24**	24	8	16	0	0	0
**25**	25	9	16	0	0	0
**26**	24	8	16	0	0	0
**27**	18	6	12	0	2	2
**28**	22	6	16	2	0	2
**29**	22	6	16	1	0	1
**30**	23	8	15	0	0	0
**TOTAL**	**653**	**206**	**447**	**9**	**16**	**25**
**AVERAGE**	**21.8**	**6.9**	**14.9**	**0.3**	**0.5**	**0.8**
SEM	**0.9**	**0.6**	**0.4**	**0.1**	**0.2**	**0.2**
Stat. sig. (inter-clone) ‡p<0.05, ‡‡p<0.005		‡‡				
p-value (2-tailed students t-test)		7.46E-16		3.13E-01		
Stat. sig. (exp. vs. con embryo) *p<0.05, **p<0.005	**	**	**	*		*
p-value (2-tailed students t-test)	1.23E-08	1.95E-10	4.18E-03	2.01E-02	1.24E-01	2.15E-02

Supplementary Tables ST11

Control siRNA (10µM), 1in2 micro-injection, >64-cell (E4.5) stage						
#	Total cell number per embryo	Cells in injected clone	Cells in non-injected clone	Dividing cells in injected clone	Dividing cells in non-injected clone	Total number of dividing cells
**1**	75	36	39	1	1	2
**2**	69	33	36	2	1	3
**3**	62	29	33	1	1	2
**4**	68	35	33	0	1	1
**5**	65	32	33	1	0	1
**6**	86	42	44	2	0	2
**7**	67	33	34	2	0	2
**8**	76	37	39	1	0	1
**9**	68	33	35	1	1	2
**10**	77	38	39	3	0	3
**11**	80	36	44	1	0	1
**12**	68	34	34	2	0	2
**13**	80	39	41	0	0	0
**TOTAL**	**941**	**457**	**484**	**17**	**5**	**22**
**AVERAGE**	**72.4**	**35.2**	**37.2**	**1.3**	**0.4**	**1.7**
SEM	**2.0**	**0.9**	**1.1**	**0.2**	**0.1**	**0.2**
Stat. sig. (inter-clone) ‡p<0.05, ‡‡p<0.005				‡‡		
p-value (2-tailed students t-test)		1.69E-01		2.67E-03		

Wwc2-specific siRNA (10µM), 1in2 micro-injection, >64-cell (E4.5) stage						
#	Total cell number per embryo	Cells in injected clone	Cells in non-injected clone	Dividing cells in injected clone	Dividing cells in non-injected clone	Total number of dividing cells
**1**	45	15	30	0	5	5
**2**	33	9	24	0	3	3
**3**	44	14	30	0	1	1
**4**	37	8	29	0	2	2
**5**	62	15	47	2	0	2
**6**	45	14	31	0	3	3
**7**	44	18	26	0	1	1
**8**	61	19	42	0	3	3
**9**	69	19	50	1	4	5
**10**	53	11	42	1	2	3
**11**	59	21	38	1	2	3
**12**	52	16	36	0	1	1
**13**	43	16	27	1	5	6
**14**	57	17	40	1	1	2
**15**	54	14	40	0	2	2
**16**	54	17	37	0	4	4
**TOTAL**	**812**	**243**	**569**	**7**	**39**	**46**
**AVERAGE**	**50.8**	**15.2**	**35.6**	**0.4**	**2.4**	**2.9**
SEM	**2.4**	**0.9**	**1.9**	**0.2**	**0.4**	**0.4**
Stat. sig. (inter-clone) ‡p<0.05, ‡‡p<0.005		‡‡		‡‡		
p-value (2-tailed students t-test)		1.21E-10		3.03E-05		
Stat. sig. (exp. vs. con embryo) *p<0.05, **p<0.005	**	**		**	**	*
p-value (2-tailed students t-test)	3.49E-07	7.49E-15	4.88E-01	3.89E-03	6.90E-05	1.78E-02

Supplementary Tables ST12

Control (GFP) dsRNA (300ng/µl), 1in2 micro-injection, >64-cell (E4.5) stage

#	Total cell number per embryo	Cells in injected clone	Cells in non-injected clone
1	63	not assayed	not assayed
2	99	not assayed	not assayed
3	55	not assayed	not assayed
4	83	not assayed	not assayed
5	102	not assayed	not assayed
6	76	not assayed	not assayed
7	83	not assayed	not assayed
8	83	38	45
9	75	37	38
10	91	44	47
11	82	41	41
12	71	38	33
13	76	39	37
14	89	46	43
15	93	45	48
16	68	27	41
17	86	44	42
TOTAL	1375	399	415
AVERAGE	80.9	39.9	41.5
SEM	3.0	1.8	1.5
Stat. sig. (inter-clone) ‡p<0.05, ‡‡p<0.005			
p-value (2-tailed students t-test)		4.94E-01	

Wwc2-specific (CDS) dsRNA (300ng/µl), 1in2 micro-injection, >64-cell (E4.5) stage

#	Total cell number per embryo	Cells in injected clone	Cells in non-injected clone
1	77	not assayed	not assayed
2	80	not assayed	not assayed
3	55	not assayed	not assayed
4	41	not assayed	not assayed
5	33	not assayed	not assayed
6	44	not assayed	not assayed
7	43	not assayed	not assayed
8	23	not assayed	not assayed
9	42	not assayed	not assayed
10	45	not assayed	not assayed
TOTAL	483	n/a	n/a
AVERAGE	48.3	n/a	n/a
SEM	5.7	n/a	n/a
Stat. sig. (inter-clone) ‡p<0.05, ‡‡p<0.005			
p-value (2-tailed students t-test)		n/a	
Stat. sig. (exp. vs. con embryo) *p<0.05, **p<0.005	**		
p-value (2-tailed students t-test)	8.35E-06	n/a	n/a

Kibra-specific (CDS) dsRNA (300ng/µl), 1in2 micro-injection, >64-cell (E4.5) stage

#	Total cell number per embryo	Cells in injected clone	Cells in non-injected clone
1	91	45	46
2	89	45	44
3	80	48	32
4	83	35	48
5	96	57	39
6	66	19	47
7	63	42	21
8	88	46	42
9	90	30	60
TOTAL	746	367	379
AVERAGE	82.9	40.8	42.1
SEM	3.8	3.7	3.6
Stat. sig. (inter-clone) ‡p<0.05, ‡‡p<0.005			
p-value (2-tailed students t-test)		8.01E-01	
Stat. sig. (exp. vs. con embryo) *p<0.05, **p<0.005			
p-value (2-tailed students t-test)	6.91E-01	8.29E-01	8.73E-01
Stat. sig. (exp. vs. Wwc2 (CDS) dsRNA embryo) §p<0.05, §§p<0.005	§§		
p-value (2-tailed students t-test)	1.23E-04	n/a	n/a

Kibra & Wwc2-specific (CDS) dsRNA (300ng/µl), 1in2 micro-injection, >64-cell (E4.5) stage

#	Total cell number per embryo	Cells in injected clone	Cells in non-injected clone
1	55	17	38
2	42	14	28
3	59	15	44
4	58	15	43
5	62	21	41
TOTAL	276	82	194
AVERAGE	55.2	16.4	38.8
SEM	3.5	1.2	2.9
Stat. sig. (inter-clone) ‡p<0.05, ‡‡p<0.005		‡‡	
p-value (2-tailed students t-test)		1.00E-04	
Stat. sig. (exp. vs. con embryo) *p<0.05, **p<0.005	**	**	
p-value (2-tailed students t-test)	3.19E-04	8.20E-07	3.66E-01
Stat. sig. (exp. vs. Wwc2 (CDS) dsRNA embryo) §p<0.05, §§p<0.005			
p-value (2-tailed students t-test)	4.32E-01	n/a	n/a
Stat. sig. (exp. vs. Kibra (CDS) dsRNA embryo) +p<0.05, ++p<0.005	++	++	
p-value (2-tailed students t-test)	4.34E-04	5.09E-04	5.51E-01

Supplementary Tables ST13

Control (GFP) dsRNA (300ng/µl), 1in2 micro-injection, 16-cell (E3.0) stage									
#	Total cell number per embryo	Cells in injected clone	Cells in non-injected clone	Total OUTER cells per embryo	OUTER CELLS		Total INNER cells per embryo	INNER CELLS	
					Injected clone	Non-Injected clone		Injected clone	Non-Injected clone
**1**	17	8	9	9	4	5	8	4	4
**2**	16	8	8	10	5	5	6	3	3
**3**	16	8	8	12	6	6	4	2	2
**4**	17	9	8	10	5	5	7	4	3
**5**	16	8	8	11	5	6	5	3	2
**6**	16	8	8	11	6	5	5	2	3
**7**	15	7	8	10	5	5	5	2	3
**8**	16	8	8	11	5	6	5	3	2
**TOTAL**	**129**	**64**	**65**	**84**	**41**	**43**	**45**	**23**	**22**
**AVERAGE**	**16.1**	**8.0**	**8.1**	**10.5**	**5.1**	**5.4**	**5.6**	**2.9**	**2.8**
SEM	**0.2**	**0.2**	**0.1**	**0.3**	**0.2**	**0.2**	**0.5**	**0.3**	**0.3**
Stat. sig. (inter-clone) ‡p<0.05, ‡‡p<0.005									
p-value (2-tailed students t-test)		5.90E-01			4.05E-01			7.51E-01	

Wwc2-specific (CDS) dsRNA (300ng/µl), 1in2 micro-injection, 16-cell (E3.0) stage									
#	Total cell number per embryo	Cells in injected clone	Cells in non-injected clone	Total OUTER cells per embryo	OUTER CELLS		Total INNER cells per embryo	INNER CELLS	
					Injected clone	Non-Injected clone		Injected clone	Non-Injected clone
**1**	12	4	8	7	2	5	5	2	3
**2**	12	4	8	8	3	5	4	1	3
**3**	14	6	8	10	6	4	4	0	4
**4**	16	8	8	11	6	5	5	2	3
**5**	12	4	8	8	4	4	4	0	4
**6**	13	4	9	8	4	4	5	0	5
**7**	16	8	8	10	5	5	6	3	3
**8**	12	4	8	9	4	5	3	0	3
**9**	16	8	8	13	7	6	3	1	2
**10**	19	10	9	15	8	7	4	2	2
**TOTAL**	**142**	**60**	**82**	**99**	**49**	**50**	**43**	**11**	**32**
**AVERAGE**	**14.2**	**6.0**	**8.2**	**9.9**	**4.9**	**5.0**	**4.3**	**1.1**	**3.2**
SEM	**0.8**	**0.7**	**0.1**	**0.8**	**0.6**	**0.3**	**0.3**	**0.3**	**0.3**
Stat. sig. (inter-clone) ‡p<0.05, ‡‡p<0.005		‡						‡‡	
p-value (2-tailed students t-test)		8.32E-03			3.70E-01			2.07E-04	
Stat. sig. (exp. vs. con embryo) *p<0.05, **p<0.005	*	*					*	**	
p-value (2-tailed students t-test)	4.63E-02	2.98E-02	6.93E-01	5.33E-01	7.48E-01	3.29E-01	2.37E-02	1.68E-03	2.71E-01

Supplementary Tables ST14

IVM culturing control, non-micro-injected GV stage (+18h IBMX & 16h post IBMX wash-out)

#	Meiotic spindle status				1st meiotic polar body (PB1)	
	Normal MII arrested (+PB1)	Normal MI stage (no PB1)	Defective (e.g. dispersed chromosomes, no PB1)	Absent with ultra condensed chromatin	Present	Absent
**1**	0	1	0	0	0	1
**2**	1	0	0	0	1	0
**3**	1	0	0	0	1	0
**4**	1	0	0	0	1	0
**5**	1	0	0	0	1	0
**6**	1	0	0	0	1	0
**7**	1	0	0	0	1	0
**8**	1	0	0	0	1	0
**9**	1	0	0	0	1	0
**10**	1	0	0	0	1	0
**11**	1	0	0	0	1	0
**12**	1	0	0	0	1	0
**13**	1	0	0	0	1	0
**14**	1	0	0	0	1	0
**15**	1	0	0	0	1	0
**16**	1	0	0	0	1	0
**17**	1	0	0	0	1	0
**18**	1	0	0	0	1	0
**TOTAL**	**17**	**1**	**0**	**0**	**17**	**1**
**PERCENTAGE**	**94.4**	**5.6**	**0.0**	**0.0**	**94.4**	**5.6**

Control siRNA microinjected GV stage (+18h IBMX & 16h post IBMX wash-out)

#	Meiotic spindle status				1st meiotic polar body (PB1)	
	Normal MII arrested (+PB1)	Normal MI stage (no PB1)	Defective (e.g. dispersed chromosomes, no PB1)	Absent with ultra condensed chromatin	Present	Absent
**1**	1	0	0	0	1	0
**2**	1	0	0	0	1	0
**3**	1	0	0	0	1	0
**4**	1	0	0	0	1	0
**5**	0	1	0	0	0	1
**6**	1	0	0	0	1	0
**7**	1	0	0	0	1	0
**8**	1	0	0	0	1	0
**9**	1	0	0	0	1	0
**10**	1	0	0	0	1	0
**11**	1	0	0	0	1	0
**12**	1	0	0	0	1	0
**13**	1	0	0	0	1	0
**14**	1	0	0	0	1	0
**15**	1	0	0	0	1	0
**16**	1	0	0	0	1	0
**17**	1	0	0	0	1	0
**18**	1	0	0	0	1	0
**19**	1	0	0	0	1	0
**20**	1	0	0	0	1	0
**21**	1	0	0	0	1	0
**22**	1	0	0	0	1	0
**23**	0	1	0	0	0	1
**24**	1	0	0	0	1	0
**25**	1	0	0	0	1	0
**26**	1	0	0	0	1	0
**27**	1	0	0	0	1	0
**28**	1	0	0	0	1	0
**TOTAL**	**26**	**2**	**0**	**0**	**26**	**2**
**PERCENTAGE**	**92.9**	**7.1**	**0.0**	**0.0**	**92.9**	**7.1**
Stat. sig. con. (siRNA) vs. con. (IVM) embryo ‡p<0.05, ‡‡p<0.005						
p-value (2-tailed students t-test)	8.36E-01	8.36E-01	-	-	8.36E-01	8.36E-01

Control siRNA microinjected GV stage (+18h IBMX & 16h post IBMX wash-out)

#	Meiotic spindle status				1st meiotic polar body (PB1)	
	Normal MII arrested (+PB1)	Normal MI stage (no PB1)	Defective (e.g. dispersed chromosomes, no PB1)	Absent with ultra condensed chromatin	Present	Absent
**1**	0	0	1	0	0	1
**2**	0	0	1	0	0	1
**3**	0	0	1	0	0	1
**4**	1	0	0	0	1	0
**5**	0	0	1	0	0	1
**6**	0	1	0	0	0	1
**7**	0	1	0	0	0	1
**8**	0	0	0	1	0	1
**9**	0	1	0	0	0	1
**10**	0	0	0	1	0	1
**11**	0	0	0	1	0	1
**12**	0	0	0	1	0	1
**13**	0	1	0	0	0	1
**14**	0	1	0	0	0	1
**15**	0	1	0	0	0	1
**16**	1	0	0	0	1	0
**17**	0	0	0	1	0	1
**18**	0	0	1	0	0	1
**19**	0	0	0	1	0	1
**20**	0	0	0	1	0	1
**21**	0	1	0	0	0	1
**22**	0	0	1	0	0	1
**23**	0	1	0	0	0	1
**24**	0	0	1	0	0	1
**25**	0	1	0	0	0	1
**26**	0	1	0	0	0	1
**27**	0	1	0	0	0	1
**28**	0	1	0	0	0	1
**29**	0	0	1	0	0	1
**30**	0	1	0	0	0	1
**31**	0	1	0	0	0	1
**32**	1	0	0	0	1	0
**33**	0	0	0	1	0	1
**34**	0	0	0	1	0	1
**35**	0	1	0	0	0	1
**TOTAL**	**3**	**15**	**8**	**9**	**3**	**32**
**PERCENTAGE**	**8.6**	**42.9**	**22.9**	**25.7**	**8.6**	**91.4**
Stat. sig. exp. vs. con. (IVM) embryo ‡p<0.05, ‡‡p<0.005	‡‡	‡‡	‡	‡	‡‡	‡‡
p-value (2-tailed students t-test)	4.37E-15	4.44E-03	2.78E-02	1.78E-02	4.37E-15	4.37E-15
Stat. sig. exp. vs. con. (siRNA) embryo *p<0.05, **p<0.005	**	**	*	**	**	**
p-value (2-tailed students t-test)	7.26E-18	1.17E-03	6.22E-03	3.25E-03	7.26E-18	7.26E-18

Supplementary Tables ST15

Control siRNA microinjected GV stage (+18h IBMX & extended 24h post IBMX wash-out)

#	Meiotic spindle status				1st meiotic polar body (PB1)	
	Normal MII arrested (+PB1)	Normal MI stage (no PB1)	Defective (e.g. dispersed chromosomes, no PB1)	Absent with ultra condensed chromatin	Present	Absent
1	0	1	0	0	0	1
2	1	0	0	0	1	0
3	1	0	0	0	1	0
4	1	0	0	0	1	0
5	1	0	0	0	1	0
6	1	0	0	0	1	0
7	1	0	0	0	1	0
8	0	1	0	0	0	1
9	1	0	0	0	1	0
10	0	1	0	0	0	1
11	0	0	1	0	0	1
12	1	0	0	0	1	0
13	1	0	0	0	1	0
14	1	0	0	0	1	0
15	1	0	0	0	1	0
16	1	0	0	0	1	0
17	1	0	0	0	1	0
18	1	0	0	0	1	0
19	1	0	0	0	1	0
20	1	0	0	0	1	0
21	1	0	0	0	1	0
22	1	0	0	0	1	0
23	0	1	0	0	0	1
24	1	0	0	0	1	0
25	1	0	0	0	1	0
26	1	0	0	0	1	0
27	0	1	0	0	0	1
**TOTAL**	**21**	**5**	**1**	**0**	**21**	**6**
**PERCENTAGE**	**77.8**	**18.5**	**3.7**	**0.0**	**77.8**	**22.2**

Wwc2 siRNA microinjected GV stage (+18h IBMX & extended 24h post IBMX wash-out)

#	Meiotic spindle status				1st meiotic polar body (PB1)	
	Normal MII arrested (+PB1)	Normal MI stage (no PB1)	Defective (e.g. dispersed chromosomes, no PB1)	Absent with ultra condensed chromatin	Present	Absent
1	0	0	1	0	0	1
2	0	1	0	0	0	1
3	0	0	1	0	0	1
4	0	0	1	0	0	1
5	0	0	1	0	0	1
6	0	0	1	0	0	1
7	0	0	1	0	0	1
8	0	0	1	0	0	1
9	0	1	0	0	0	1
10	0	0	1	0	0	1
11	0	0	1	0	0	1
12	0	0	0	1	0	1
13	1	0	0	0	1	0
14	1	0	0	0	1	0
15	0	0	1	0	0	1
16	0	0	1	0	0	1
17	0	0	1	0	0	1
18	0	0	1	0	0	1
19	0	0	1	0	0	1
20	0	0	1	0	0	1
21	0	0	1	0	0	1
22	0	0	0	1	0	1
23	0	1	0	0	0	1
24	0	1	0	0	0	1
25	0	0	1	0	0	1
26	0	0	0	1	0	1
27	0	0	1	0	0	1
28	0	0	1	0	0	1
29	0	0	1	0	0	1
30	0	0	1	0	0	1
31	0	1	0	0	0	1
32	0	0	0	1	0	1
**TOTAL**	**2**	**5**	**21**	**4**	**2**	**30**
**PERCENTAGE**	**6.3**	**15.6**	**65.6**	**12.5**	**6.3**	**93.8**
Stat. sig. *Wwc2* KD vs. con. siRNA embryo ‡p<0.05, ‡‡p<0.005	‡‡		‡‡		‡‡	‡‡
p-value (2-tailed students t-test)	5.07E-11	7.73E-01	5.49E-08	5.85E-02	5.07E-11	5.07E-11

Supplementary Tables ST16

Control siRNA microinjected GV stage (+18h IBMX & 1h post IBMX wash-out)

#	Meiotic spindle status						1st meiotic polar body (PB1)	
	Normal MII arrested (+PB1)	Normal MI stage (no PB1)	Defective (e.g. dispersed chromosomes, no PB1)	Absent with ultra condensed chromatin	GV intact	GVBD (no spindle)	Present	Absent
**1**	0	0	0	0	1	0	0	1
**2**	0	0	0	0	0	1	0	1
**3**	0	0	0	0	0	1	0	1
**4**	0	0	0	0	0	1	0	1
**5**	0	0	0	0	0	1	0	1
**6**	0	0	0	0	0	1	0	1
**7**	0	0	0	0	0	1	0	1
**8**	0	0	0	0	0	1	0	1
**9**	0	0	0	0	0	1	0	1
**10**	0	0	0	0	0	1	0	1
**11**	0	0	0	0	0	1	0	1
**12**	0	0	0	0	0	1	0	1
**13**	0	0	0	0	0	1	0	1
**14**	0	0	0	0	0	1	0	1
**TOTAL**	**0**	**0**	**0**	**0**	**1**	**13**	**0**	**14**
**PERCENTAGE**	**0.0**	**0.0**	**0.0**	**0.0**	**7.1**	**92.9**	**0.0**	**100.0**

Wwc2 siRNA microinjected GV stage (+18h IBMX & 1h post IBMX wash-out)

#	Meiotic spindle status						1st meiotic polar body (PB1)	
	Normal MII arrested (+PB1)	Normal MI stage (no PB1)	Defective (e.g. dispersed chromosomes, no PB1)	Absent with ultra condensed chromatin	GV intact	GVBD (no spindle)	Present	Absent
**1**	0	0	0	0	1	0	0	1
**2**	0	0	0	0	0	1	0	1
**3**	0	0	0	0	1	0	0	1
**4**	0	0	0	0	1	0	0	1
**5**	0	0	0	0	1	0	0	1
**6**	0	0	0	0	0	1	0	1
**7**	0	0	0	0	0	1	0	1
**8**	0	0	0	0	0	1	0	1
**9**	0	0	0	1	0	0	0	1
**10**	0	0	0	0	1	0	0	1
**11**	0	0	0	0	0	1	0	1
**12**	0	0	0	0	1	0	0	1
**13**	0	0	0	0	0	1	0	1
**14**	0	0	0	0	0	1	0	1
**15**	0	0	0	0	0	1	0	1
**TOTAL**	**0**	**0**	**0**	**1**	**6**	**8**	**0**	**15**
**PERCENTAGE**	**0.0**	**0.0**	**0.0**	**6.7**	**40.0**	**53.3**	**0.0**	**100.0**
Stat. sig. *Wwc2* siRNA vs. con. siRNA embryo ‡p<0.05, ‡‡p<0.005					‡	‡		
p-value (2-tailed students t-test)	n/a	n/a	n/a	3.43E-01	3.99E-02	1.64E-02	n/a	n/a

Wwc2 siRNA + *Wwc2*-HA mRNA microinjected GV stage (+18h IBMX & 1h post IBMX wash-out)

#	Meiotic spindle status						1st meiotic polar body (PB1)	
	Normal MII arrested (+PB1)	Normal MI stage (no PB1)	Defective (e.g. dispersed chromosomes, no PB1)	Absent with ultra condensed chromatin	GV intact	GVBD (no spindle)	Present	Absent
**1**	0	0	0	0	1	0	0	1
**2**	0	0	0	0	0	1	0	1
**3**	0	0	0	0	0	1	0	1
**4**	0	0	0	0	0	1	0	1
**5**	0	0	0	0	0	1	0	1
**6**	0	0	0	0	0	1	0	1
**7**	0	0	0	0	0	1	0	1
**8**	0	0	0	0	0	1	0	1
**9**	0	0	0	0	0	1	0	1
**10**	0	0	0	0	0	1	0	1
**11**	0	0	0	0	0	1	0	1
**12**	0	0	0	0	0	1	0	1
**13**	0	0	0	0	0	1	0	1
**14**	0	0	0	0	0	1	0	1
**15**	0	0	0	0	0	1	0	1
**16**	0	0	0	0	1	0	0	1
**TOTAL**	**0**	**0**	**0**	**0**	**2**	**14**	**0**	**16**
**PERCENTAGE**	**0.0**	**0.0**	**0.0**	**0.0**	**12.5**	**87.5**	**0.0**	**100.0**
Stat. sig. *Wwc2* KD (rescue) vs. con. siRNA embryo ‡p<0.05, ‡‡p<0.005								
p-value (2-tailed students t-test)	n/a	n/a	n/a	n/a	6.40E-01	6.40E-01	n/a	n/a
Stat. sig. *Wwc2* KD (rescue) vs. *Wwc2* siRNA embryo *p<0.05, **p<0.005						*		
p-value (2-tailed students t-test)	n/a	n/a	n/a	3.10E-01	8.53E-02	3.70E-02	n/a	n/a

Supplementary Tables ST17

Control siRNA microinjected GV stage (+18h IBMX & 6h post IBMX wash-out)								
#	Meiotic spindle status						1st meiotic polar body (PB1)	
	Normal MII arrested (+PB1)	Normal MI stage (no PB1)	Defective (e.g. dispersed chromosomes, no PB1)	Absent with ultra condensed chromatin	GV intact	GVBD (no spindle)	Present	Absent
**1**	0	1	0	0	0	0	0	1
**2**	0	0	1	0	0	0	0	1
**3**	0	1	0	0	0	0	0	1
**4**	0	1	0	0	0	0	0	1
**5**	0	1	0	0	0	0	0	1
**6**	0	1	0	0	0	0	0	1
**7**	0	1	0	0	0	0	0	1
**8**	0	1	0	0	0	0	0	1
**9**	0	0	1	0	0	0	0	1
**10**	0	1	0	0	0	0	0	1
**11**	0	1	0	0	0	0	0	1
**12**	0	1	0	0	0	0	0	1
**13**	0	1	0	0	0	0	0	1
**14**	0	1	0	0	0	0	0	1
**15**	0	1	0	0	0	0	0	1
**TOTAL**	**0**	**13**	**2**	**0**	**0**	**0**	**0**	**15**
**PERCENTAGE**	**0.0**	**86.7**	**13.3**	**0.0**	**0.0**	**0.0**	**0.0**	**100.0**

Wwc2 siRNA microinjected GV stage (+18h IBMX & 6h post IBMX wash-out)								
#	Meiotic spindle status						1st meiotic polar body (PB1)	
	Normal MII arrested (+PB1)	Normal MI stage (no PB1)	Defective (e.g. dispersed chromosomes, no PB1)	Absent with ultra condensed chromatin	GV intact	GVBD (no spindle)	Present	Absent
**1**	0	0	1	0	0	0	0	1
**2**	0	1	0	0	0	0	0	1
**3**	0	0	1	0	0	0	0	1
**4**	0	0	1	0	0	0	0	1
**5**	0	1	0	0	0	0	0	1
**6**	0	0	1	0	0	0	0	1
**7**	0	0	1	0	0	0	0	1
**8**	0	0	0	1	0	0	0	1
**9**	0	1	0	0	0	0	0	1
**10**	0	0	0	1	0	0	0	1
**11**	0	1	0	0	0	0	0	1
**12**	0	1	0	0	0	0	0	1
**13**	0	0	0	0	1	0	0	1
**14**	0	1	0	0	0	0	0	1
**15**	0	0	0	0	1	0	0	1
**16**	0	0	0	0	1	0	0	1
**17**	0	1	0	0	0	0	0	1
**18**	0	1	0	0	0	0	0	1
**TOTAL**	**0**	**8**	**5**	**2**	**3**	**0**	**0**	**18**
**PERCENTAGE**	**0.0**	**44.4**	**27.8**	**11.1**	**16.7**	**0.0**	**0.0**	**100.0**
Stat. sig. *Wwc2* siRNA vs. con. siRNA embryo ‡p<0.05, ‡‡p<0.005		‡						
p-value (2-tailed students t-test)	n/a	1.10E-02	3.27E-01	1.94E-01	1.03E-01	n/a	n/a	n/a

Wwc2 siRNA + *Wwc2*-HA mRNA microinjected GV stage (+18h IBMX & 6h post IBMX wash-out)								
#	Meiotic spindle status						1st meiotic polar body (PB1)	
	Normal MII arrested (+PB1)	Normal MI stage (no PB1)	Defective (e.g. dispersed chromosomes, no PB1)	Absent with ultra condensed chromatin	GV intact	GVBD (no spindle)	Present	Absent
**1**	0	1	0	0	0	0	0	1
**2**	0	1	0	0	0	0	0	1
**3**	0	1	0	0	0	0	0	1
**4**	0	1	0	0	0	0	0	1
**5**	0	1	0	0	0	0	0	1
**6**	0	1	0	0	0	0	0	1
**7**	0	1	0	0	0	0	0	1
**8**	0	1	0	0	0	0	0	1
**9**	0	0	1	0	0	0	0	1
**10**	0	0	0	0	1	0	0	1
**11**	0	1	0	0	0	0	0	1
**12**	0	1	0	0	0	0	0	1
**13**	0	1	0	0	0	0	0	1
**14**	0	1	0	0	0	0	0	1
**15**	0	1	0	0	0	0	0	1
**16**	0	0	1	0	0	0	0	1
**17**	0	1	0	0	0	0	0	1
**18**	0	1	0	0	0	0	0	1
**TOTAL**	**0**	**15**	**2**	**0**	**1**	**0**	**0**	**18**
**PERCENTAGE**	**0.0**	**83.3**	**11.1**	**0.0**	**5.6**	**0.0**	**0.0**	**100.0**
Stat. sig. *Wwc2* KD (rescue) vs. con. siRNA embryo ‡p<0.05, ‡‡p<0.005		‡‡	‡‡		‡‡			
p-value (2-tailed students t-test)	n/a	7.98E-01	8.51E-01	n/a	3.70E-01	n/a	n/a	n/a
Stat. sig. *Wwc2* KD (rescue) vs. *Wwc2* siRNA embryo *p<0.05, **p<0.005		*						
p-value (2-tailed students t-test)	n/a	1.43E-02	2.18E-01	1.54E-01	3.02E-01	n/a	n/a	n/a

Supplementary Tables ST18

Control siRNA microinjected GV stage (+18h IBMX & 12h post IBMX wash-out)

#	Meiotic spindle status						1st meiotic polar body (PB1)	
	Normal MII arrested (+PB1)	Normal MI stage (no PB1)	Defective (e.g. dispersed chromosomes, no PB1)	Absent with ultra condensed chromatin	GV intact	GVBD (no spindle)	Present	Absent
**1**	1	0	0	0	0	0	1	0
**2**	1	0	0	0	0	0	1	0
**3**	0	1	0	0	0	0	0	1
**4**	0	1	0	0	0	0	0	1
**5**	1	0	0	0	0	0	1	0
**6**	1	0	0	0	0	0	1	0
**7**	1	0	0	0	0	0	1	0
**8**	1	0	0	0	0	0	1	0
**9**	1	0	0	0	0	0	1	0
**10**	1	0	0	0	0	0	1	0
**11**	1	0	0	0	0	0	1	0
**12**	0	1	0	0	0	0	0	1
**13**	1	0	0	0	0	0	1	0
**14**	1	0	0	0	0	0	1	0
**15**	0	1	0	0	0	0	0	1
**TOTAL**	**11**	**4**	**0**	**0**	**0**	**0**	**11**	**4**
**PERCENTAGE**	**73.3**	**26.7**	**0.0**	**0.0**	**0.0**	**0.0**	**73.3**	**26.7**

Wwc2 siRNA microinjected GV stage (+18h IBMX & 12h post IBMX wash-out)

#	Meiotic spindle status						1st meiotic polar body (PB1)	
	Normal MII arrested (+PB1)	Normal MI stage (no PB1)	Defective (e.g. dispersed chromosomes, no PB1)	Absent with ultra condensed chromatin	GV intact	GVBD (no spindle)	Present	Absent
**1**	0	0	0	1	0	0	0	1
**2**	1	0	0	0	0	0	1	0
**3**	0	0	1	0	0	0	0	1
**4**	0	0	1	0	0	0	0	1
**5**	0	1	0	0	0	0	0	1
**6**	0	0	1	0	0	0	0	1
**7**	0	1	0	0	0	0	0	1
**8**	0	1	0	0	0	0	0	1
**9**	0	0	1	0	0	0	0	1
**10**	0	1	0	0	0	0	0	1
**11**	0	0	0	1	0	0	0	1
**12**	1	0	0	0	0	0	1	0
**13**	0	1	0	0	0	0	0	1
**14**	0	1	0	0	0	0	0	1
**15**	0	1	0	0	0	0	0	1
**16**	0	1	0	0	0	0	0	1
**17**	0	0	1	0	0	0	0	1
**18**	0	0	0	1	0	0	0	1
**TOTAL**	**2**	**8**	**5**	**3**	**0**	**0**	**2**	**16**
**PERCENTAGE**	**11.1**	**44.4**	**27.8**	**16.7**	**0.0**	**0.0**	**11.1**	**88.9**
Stat. sig. *Wwc2* siRNA vs. con. siRNA embryo ‡p<0.05, ‡‡p<0.005	**‡‡**		**‡**				**‡‡**	**‡‡**
p-value (2-tailed students t-test)	7.43E-05	3.05E-01	2.66E-02	1.03E-01	n/a	n/a	7.43E-05	7.43E-05

Wwc2 siRNA + *Wwc2*-HA mRNA microinjected GV stage (+18h IBMX & 12h post IBMX wash-out)

#	Meiotic spindle status						1st meiotic polar body (PB1)	
	Normal MII arrested (+PB1)	Normal MI stage (no PB1)	Defective (e.g. dispersed chromosomes, no PB1)	Absent with ultra condensed chromatin	GV intact	GVBD (no spindle)	Present	Absent
**1**	0	1	0	0	0	0	0	1
**2**	1	0	0	0	0	0	1	0
**3**	0	1	0	0	0	0	0	1
**4**	1	0	0	0	0	0	1	0
**5**	1	0	0	0	0	0	1	0
**6**	0	1	0	0	0	0	0	1
**7**	1	0	0	0	0	0	1	0
**8**	1	0	0	0	0	0	1	0
**9**	1	0	0	0	0	0	1	0
**10**	1	0	0	0	0	0	1	0
**11**	1	0	0	0	0	0	1	0
**12**	1	0	0	0	0	0	1	0
**13**	1	0	0	0	0	0	1	0
**14**	1	0	0	0	0	0	1	0
**15**	0	0	1	0	0	0	0	1
**16**	0	0	1	0	0	0	0	1
**17**	1	0	0	0	0	0	1	0
**TOTAL**	**12**	**3**	**2**	**0**	**0**	**0**	**12**	**5**
**PERCENTAGE**	**70.6**	**17.6**	**11.8**	**0.0**	**0.0**	**0.0**	**70.6**	**29.4**
Stat. sig. *Wwc2* KD (rescue) vs. con. siRNA embryo ‡p<0.05, ‡‡p<0.005								
p-value (2-tailed students t-test)	8.69E-01	5.53E-01	1.81E-01	n/a	n/a	n/a	8.69E-01	8.69E-01
Stat. sig. *Wwc2* KD (rescue) vs. *Wwc2* siRNA embryo *p<0.05, **p<0.005	**						**	**
p-value (2-tailed students t-test)	1.11E-04	9.28E-02	2.49E-01	8.26E-02	n/a	n/a	1.11E-04	1.11E-04

Supplementary Tables ST19

Control siRNA microinjected GV stage (+18h IBMX & 16h post IBMX wash-out)								
#	Meiotic spindle status						1st meiotic polar body (PB1)	
	Normal MII arrested (+PB1)	Normal MI stage (no PB1)	Defective (e.g. dispersed chromosomes, no PB1)	Absent with ultra condensed chromatin	GV intact	GVBD (no spindle)	Present	Absent
**1**	0	1	0	0	0	0	0	1
**2**	1	0	0	0	0	0	1	0
**3**	1	0	0	0	0	0	1	0
**4**	1	0	0	0	0	0	1	0
**5**	1	0	0	0	0	0	1	0
**6**	1	0	0	0	0	0	1	0
**7**	1	0	0	0	0	0	1	0
**8**	1	0	0	0	0	0	1	0
**9**	1	0	0	0	0	0	1	0
**10**	1	0	0	0	0	0	1	0
**11**	1	0	0	0	0	0	1	0
**12**	1	0	0	0	0	0	1	0
**13**	1	0	0	0	0	0	1	0
**14**	0	1	0	0	0	0	0	1
**15**	0	1	0	0	0	0	0	1
**16**	1	0	0	0	0	0	1	0
**17**	0	0	1	0	0	0	0	1
**18**	1	0	0	0	0	0	1	0
**19**	1	0	0	0	0	0	1	0
**20**	1	0	0	0	0	0	1	0
**21**	1	0	0	0	0	0	1	0
**22**	1	0	0	0	0	0	1	0
**TOTAL**	**18**	**3**	**1**	**0**	**0**	**0**	**18**	**4**
**PERCENTAGE**	**81.8**	**13.6**	**4.5**	**0.0**	**0.0**	**0.0**	**81.8**	**18.2**

Wwc2 siRNA microinjected GV stage (+18h IBMX & 16h post IBMX wash-out)								
#	Meiotic spindle status						1st meiotic polar body (PB1)	
	Normal MII arrested (+PB1)	Normal MI stage (no PB1)	Defective (e.g. dispersed chromosomes, no PB1)	Absent with ultra condensed chromatin	GV intact	GVBD (no spindle)	Present	Absent
**1**	0	0	1	0	0	0	0	1
**2**	0	0	0	1	0	0	0	1
**3**	0	0	0	1	0	0	0	1
**4**	0	1	0	0	0	0	0	1
**5**	0	0	1	0	0	0	0	1
**6**	0	1	0	0	0	0	0	1
**7**	0	0	0	1	0	0	0	1
**8**	0	0	1	0	0	0	0	1
**9**	0	0	1	0	0	0	0	1
**10**	0	1	0	0	0	0	0	1
**11**	0	1	0	0	0	0	0	1
**12**	0	1	0	0	0	0	0	1
**13**	0	1	0	0	0	0	0	1
**14**	1	0	0	0	0	0	1	0
**15**	1	0	0	0	0	0	1	0
**16**	0	1	0	0	0	0	0	1
**17**	0	0	1	0	0	0	0	1
**18**	0	1	0	0	0	0	0	1
**19**	0	1	0	0	0	0	0	1
**20**	0	1	0	0	0	0	0	1
**21**	0	0	1	0	0	0	0	1
**22**	0	0	0	1	0	0	0	1
**23**	0	0	0	1	0	0	0	1
**TOTAL**	**2**	**10**	**6**	**5**	**0**	**0**	**2**	**21**
**PERCENTAGE**	**8.7**	**43.5**	**26.1**	**21.7**	**0.0**	**0.0**	**8.7**	**91.3**
Stat. sig. *Wwc2* siRNA vs. con. siRNA embryo ‡p<0.05, ‡‡p<0.005	‡‡	‡	‡	‡			‡‡	‡‡
p-value (2-tailed students t-test)	8.61E-09	2.73E-02	4.75E-02	2.00E-02	n/a	n/a	8.61E-09	8.61E-09

Wwc2 siRNA + *Wwc2*-HA mRNA microinjected GV stage (+18h IBMX & 16h post IBMX wash-out)								
#	Meiotic spindle status						1st meiotic polar body (PB1)	
	Normal MII arrested (+PB1)	Normal MI stage (no PB1)	Defective (e.g. dispersed chromosomes, no PB1)	Absent with ultra condensed chromatin	GV intact	GVBD (no spindle)	Present	Absent
**1**	0	0	1	0	0	0	0	1
**2**	1	0	0	0	0	0	1	0
**3**	0	1	0	0	0	0	0	1
**4**	0	0	1	0	0	0	0	1
**5**	1	0	0	0	0	0	1	0
**6**	1	0	0	0	0	0	1	0
**7**	1	0	0	0	0	0	1	0
**8**	0	1	0	0	0	0	0	1
**9**	1	0	0	0	0	0	1	0
**10**	1	0	0	0	0	0	1	0
**11**	0	1	0	0	0	0	0	1
**12**	1	0	0	0	0	0	1	0
**13**	1	0	0	0	0	0	1	0
**14**	1	0	0	0	0	0	1	0
**15**	1	0	0	0	0	0	1	0
**16**	0	1	0	0	0	0	0	1
**17**	1	0	0	0	0	0	1	0
**18**	1	0	0	0	0	0	1	0
**19**	1	0	0	0	0	0	1	0
**20**	1	0	0	0	0	0	1	0
**21**	1	0	0	0	0	0	1	0
**22**	1	0	0	0	0	0	1	0
**23**	1	0	0	0	0	0	1	0
**24**	1	0	0	0	0	0	1	0
**25**	1	0	0	0	0	0	1	0
**TOTAL**	**19**	**4**	**2**	**0**	**0**	**0**	**19**	**6**
**PERCENTAGE**	**76.0**	**16.0**	**8.0**	**0.0**	**0.0**	**0.0**	**76.0**	**24.0**
Stat. sig. *Wwc2* KD (rescue) vs. con. siRNA embryo ‡p<0.05, ‡‡p<0.005								
p-value (2-tailed students t-test)	6.36E-01	8.25E-01	6.38E-01	#DIV/0!	n/a	n/a	6.36E-01	6.36E-01
Stat. sig. *Wwc2* KD (rescue) vs. *Wwc2* siRNA embryo *p<0.05, **p<0.005	**	*		*			**	**
p-value (2-tailed students t-test)	1.21E-07	3.70E-02	9.68E-02	1.31E-02	n/a	n/a	1.21E-07	1.21E-07

Supplementary Tables ST20

Control siRNA (10μM), 1in2 micro-injection, >64-cell (E4.5) stage, anti-CDX2 & anti-NANOG

#	Total cell number per embryo	Cells in injected clone	Cells in non-injected clone	INJECTED CLONE													NON INJECTED CLONE												
				Nuclear abnormality	OUTER CELLS						INNER CELLS						Nuclear abnormality	OUTER CELLS						INNER CELLS					
						CDX2+VE	CDX2-VE	CDX2 & NANOG+VE	NANOG-VE	Apoptotic ♣		CDX2+VE	CDX2-VE	NANOG+VE	NANOG-VE	Apoptotic ♣			CDX2+VE	CDX2-VE	CDX2 & NANOG+VE	NANOG-VE	Apoptotic ♣		CDX2+VE	CDX2-VE	NANOG+VE	NANOG-VE	Apoptotic ♣
**1**	77	37	40	0	26	25	0	1	24	1	11	0	11	8	3	1	0	32	30	0	2	28	2	8	0	8	6	2	1
**2**	83	40	43	0	29	29	0	0	29	2	11	0	11	9	2	0	0	33	32	0	1	31	1	10	0	10	5	5	1
**3**	71	34	37	0	23	21	0	2	19	0	11	0	11	7	4	1	0	29	29	0	0	29	1	8	0	8	5	3	0
**4**	84	42	42	0	32	32	0	0	32	0	10	0	10	7	3	1	0	26	25	0	1	24	2	16	0	16	10	6	1
**5**	77	38	39	0	28	26	0	2	24	1	10	0	10	8	2	0	0	30	30	0	0	30	2	9	0	9	7	2	0
**6**	81	40	41	0	27	26	0	1	25	0	13	0	13	9	4	0	0	35	33	0	2	31	0	6	0	6	3	3	2
**7**	84	43	41	0	33	32	0	1	31	1	10	0	10	6	4	2	0	29	29	0	0	29	1	12	0	12	8	4	0
**8**	86	43	43	0	34	34	0	0	34	1	9	0	9	6	3	1	0	30	27	0	3	24	2	13	0	13	10	3	0
**9**	84	41	43	0	29	27	0	2	25	2	12	0	12	9	3	0	0	34	33	0	1	32	1	9	0	9	7	2	0
**10**	77	40	37	0	30	29	0	1	28	1	10	0	10	6	4	2	0	27	27	0	0	27	1	10	0	10	8	2	1
**11**	79	41	38	0	32	30	0	2	28	0	9	0	9	6	3	1	0	28	26	0	2	24	2	10	0	10	9	1	0
**TOTAL**	**883**	**439**	**444**	**0**	**323**	**311**	**0**	**12**	**299**	**9**	**116**	**0**	**116**	**81**	**35**	**9**	**0**	**333**	**321**	**0**	**12**	**309**	**15**	**111**	**0**	**111**	**78**	**33**	**6**
**AVERAGE**	**80.3**	**39.9**	**40.4**	**0.0**	**29.4**	**28.3**	**0.0**	**1.1**	**27.2**	**0.8**	**10.5**	**0.0**	**10.5**	**7.4**	**3.2**	**0.8**	**0.0**	**30.3**	**29.2**	**0.0**	**1.1**	**28.1**	**1.4**	**10.1**	**0.0**	**10.1**	**7.1**	**3.0**	**0.5**
SEM	**1.4**	**0.8**	**0.7**	**0.0**	**1.0**	**1.1**	**0.0**	**0.3**	**1.3**	**0.2**	**0.4**	**0.0**	**0.4**	**0.4**	**0.2**	**0.2**	**0.0**	**0.9**	**0.8**	**0.0**	**0.3**	**0.9**	**0.2**	**0.8**	**0.0**	**0.8**	**0.7**	**0.4**	**0.2**
Stat. sig. (inter-clone) ‡p<0.05, ‡‡p<0.005																													
p-value (2-tailed students t-test)		6.77E-01		1.00E+00	5.00E-01	5.24E-01	1.00E+00	1.00E+00	5.71E-01	8.81E-02	6.20E-01	1.00E+00	6.20E-01	7.27E-01	7.21E-01	3.85E-01													

Wwc2-specific siRNA (10μM), 1in2 micro-injection, >64-cell (E4.5) stage, anti-CDX2 & anti-NANOG

#	Total cell number per embryo	Cells in injected clone	Cells in non-injected clone	INJECTED CLONE													NON INJECTED CLONE												
				Nuclear abnormality	OUTER CELLS						INNER CELLS						Nuclear abnormality	OUTER CELLS						INNER CELLS					
						CDX2+VE	CDX2-VE	CDX2 & NANOG+VE	NANOG-VE	Apoptotic ♣		CDX2+VE	CDX2-VE	NANOG+VE	NANOG-VE	Apoptotic ♣			CDX2+VE	CDX2-VE	CDX2 & NANOG+VE	NANOG-VE	Apoptotic ♣		CDX2+VE	CDX2-VE	NANOG+VE	NANOG-VE	Apoptotic ♣
**1**	55	22	33	4	18	16	0	2	16	0	4	0	4	2	2	3	0	23	22	0	1	22	2	10	0	10	8	2	0
**2**	55	20	35	3	15	13	2	0	15	1	5	0	5	1	4	4	0	24	23	0	1	23	1	11	0	11	7	4	1
**3**	54	17	37	0	12	5	3	4	8	2	5	0	5	3	2	2	0	25	23	0	2	23	1	12	0	12	9	3	0
**4**	65	23	42	2	20	16	3	1	19	0	3	0	3	0	3	5	0	28	27	0	1	27	3	14	0	14	10	4	0
**5**	63	20	43	2	16	11	4	1	15	0	4	0	4	1	3	2	0	30	27	0	3	27	2	13	0	13	7	6	1
**6**	65	26	39	4	23	21	2	0	23	1	3	0	3	1	2	4	0	27	26	0	1	26	2	12	0	12	9	3	0
**7**	65	23	42	2	20	18	0	2	18	1	3	0	3	0	3	3	0	26	26	0	0	26	0	16	0	16	14	2	2
**8**	66	26	40	3	22	18	3	1	21	2	4	0	4	1	3	2	0	28	27	0	1	27	1	12	0	12	8	4	0
**9**	58	18	40	2	16	15	0	1	15	1	2	0	2	2	0	5	0	30	28	0	2	28	2	10	0	10	7	3	2
**10**	63	22	41	4	18	16	0	2	16	1	4	0	4	1	3	2	0	32	32	0	0	32	1	9	0	9	7	2	0
**11**	65	22	43	3	17	13	4	0	17	0	5	0	5	2	3	1	0	30	29	0	1	29	2	13	0	13	8	5	1
**12**	67	25	42	4	21	13	6	2	19	2	4	0	4	2	2	3	0	28	28	0	0	28	2	14	0	14	9	5	0
**13**	62	23	39	3	18	15	0	3	15	2	5	0	5	3	2	2	0	26	24	0	2	24	1	13	0	13	8	5	0
**14**	53	20	33	4	16	14	1	1	15	1	4	0	4	2	2	4	0	23	23	0	0	23	1	10	0	10	8	2	1
**15**	75	26	49	3	23	18	5	0	23	2	3	0	3	0	3	5	0	35	32	0	3	32	1	14	0	14	9	5	0
**16**	64	22	42	0	18	17	0	1	17	1	4	0	4	1	3	3	0	29	28	0	1	28	1	13	0	13	9	4	1
**17**	63	23	40	3	20	18	2	0	20	2	3	0	3	0	3	3	0	27	25	0	2	25	3	13	0	13	11	2	1
**18**	63	20	43	2	16	15	0	1	15	1	4	0	4	1	3	2	0	30	30	0	0	30	1	13	0	13	7	6	0
**19**	65	26	39	4	23	17	4	2	21	2	3	0	3	1	2	3	0	27	26	0	1	26	1	12	0	12	9	3	0
**TOTAL**	**1186**	**424**	**762**	**52**	**352**	**289**	**39**	**24**	**328**	**22**	**72**	**0**	**72**	**24**	**48**	**58**	**0**	**528**	**506**	**0**	**22**	**506**	**28**	**234**	**0**	**234**	**164**	**70**	**10**
**AVERAGE**	**62.4**	**22.3**	**40.1**	**2.7**	**18.5**	**15.2**	**2.1**	**1.3**	**17.3**	**1.2**	**3.8**	**0.0**	**3.8**	**1.3**	**2.5**	**3.1**	**0.0**	**27.8**	**26.6**	**0.0**	**1.2**	**26.6**	**1.5**	**12.3**	**0.0**	**12.3**	**8.6**	**3.7**	**0.5**
SEM	**1.2**	**0.6**	**0.9**	**0.3**	**0.7**	**0.8**	**0.4**	**0.3**	**0.8**	**0.2**	**0.2**	**0.0**	**0.2**	**0.2**	**0.2**	**0.3**	**0.0**	**0.7**	**0.7**	**0.0**	**0.2**	**0.7**	**0.2**	**0.4**	**0.0**	**0.4**	**0.4**	**0.3**	**0.2**
Stat. sig. (inter-clone) ‡p<0.05, ‡‡p<0.005		‡‡		‡‡	‡‡	‡‡	‡‡		‡‡		‡‡		‡‡	‡‡	‡‡	‡‡													
p-value (2-tailed students t-test)		1.82E-18		1.73E-11	3.66E-11	3.77E-13	5.52E-05	7.55E-01	1.32E-10	2.13E-01	1.61E-20	1.00E+00	1.61E-20	2.24E-18	3.47E-03	1.45E-09													
Stat. sig. (exp. vs. con embryo) *p<0.05, **p<0.005	**	**		**	**	**	**		**		**		**	**	*	**		*	*			*		*		*	*		
p-value (2-tailed students t-test)	4.80E-10	2.16E-16	8.40E-01	6.57E-08	7.58E-10	1.74E-10	1.78E-03	6.56E-01	2.11E-07	2.48E-01	7.62E-17	1.00E+00	7.62E-17	6.44E-15	4.16E-02	4.80E-06	1.00E+00	3.72E-02	2.56E-02	1.00E+00	8.60E-01	2.00E-01	6.97E-01	1.07E-02	1.00E+00	1.07E-02	4.14E-02	2.13E-01	9.42E-01

Supplementary Tables ST21

Control siRNA (10μM), 1in2 micro-injection, >64-cell (E4.5) stage, anti-GATA4 & anti-NANOG

#	Total cell number per embryo	Cells in injected clone	Cells in non-injected clone	INJECTED CLONE															NON INJECTED CLONE														
				Nuclear abnormality	OUTER CELLS							INNER CELLS							Nuclear abnormality	OUTER CELLS							INNER CELLS						
						GATA4+VE	GATA4-VE	NANOG+VE	NANOG-VE	GATA4 & NANOG -VE	Apoptotic ♣		GATA4+VE	GATA4-VE	NANOG+VE	NANOG-VE	GATA4 & NANOG -VE	Apoptotic ♣			GATA4+VE	GATA4-VE	NANOG+VE	NANOG-VE	GATA4 & NANOG -VE	Apoptotic ♣		GATA4+VE	GATA4-VE	NANOG+VE	NANOG-VE	GATA4 & NANOG -VE	Apoptotic ♣
**1**	80	38	42	0	26	0	26	2	24	24	1	12	3	9	9	3	0	0	0	32	0	32	1	31	31	2	10	2	8	8	2	0	1
**2**	88	45	43	0	34	0	34	1	33	33	3	11	4	7	7	4	0	2	0	29	0	29	3	26	26	2	14	5	9	9	5	0	2
**3**	84	41	43	0	28	0	28	0	28	28	2	13	6	7	7	6	0	1	0	30	0	30	0	30	30	2	13	3	10	10	3	0	1
**4**	91	46	45	0	36	0	36	2	34	34	2	10	4	6	6	4	0	1	0	29	0	29	1	28	28	2	16	5	11	11	5	0	3
**5**	87	43	44	0	29	0	29	1	28	28	1	14	3	11	11	3	0	1	0	33	0	33	2	31	31	2	11	5	6	6	5	0	2
**6**	80	38	42	0	27	0	27	2	25	25	2	11	3	8	8	3	0	1	0	32	0	32	1	31	31	3	10	4	6	6	4	0	2
**7**	84	43	41	0	33	0	33	0	33	33	2	10	4	6	6	4	0	2	0	28	0	28	0	28	28	1	13	3	10	10	3	0	0
**8**	81	41	40	0	26	0	26	2	24	24	1	15	3	12	12	3	0	0	0	32	0	32	3	29	29	2	8	2	6	6	2	0	1
**9**	83	43	40	0	30	0	30	2	28	28	3	13	4	9	9	4	0	1	0	29	0	29	1	28	28	1	11	3	8	8	3	0	1
**10**	83	42	41	0	31	0	31	0	31	31	1	11	4	7	7	4	0	0	0	27	0	27	0	27	27	2	14	3	11	11	3	0	2
**11**	84	40	44	0	30	0	30	3	27	27	2	10	2	8	8	2	0	1	0	31	0	31	2	29	29	1	13	3	10	10	3	0	2
**12**	78	38	40	0	27	0	27	3	24	24	3	11	2	9	9	2	0	1	0	29	0	29	0	29	29	1	11	4	7	7	4	0	1
**13**	83	41	42	0	28	0	28	1	27	27	1	13	5	8	8	5	0	1	0	32	0	32	1	31	31	2	10	2	8	8	2	0	0
**14**	90	46	44	0	30	0	30	2	28	28	0	16	4	12	12	4	0	1	0	32	0	32	0	32	32	3	12	4	8	8	4	0	1
**15**	78	39	39	0	27	0	27	2	25	25	2	12	3	9	9	3	0	2	0	28	0	28	1	27	27	1	11	2	9	9	2	0	1
**16**	85	43	42	0	29	0	29	1	28	28	1	14	3	11	11	3	0	0	0	31	0	31	2	29	29	2	11	3	8	8	3	0	0
**17**	86	43	43	0	32	0	32	0	32	32	2	11	3	8	8	3	0	1	0	30	0	30	2	28	28	1	13	4	9	9	4	0	0
**TOTAL**	**1425**	**710**	**715**	**0**	**503**	**0**	**503**	**24**	**479**	**479**	**29**	**207**	**60**	**147**	**147**	**60**	**0**	**16**	**0**	**514**	**0**	**514**	**20**	**494**	**494**	**30**	**201**	**57**	**144**	**144**	**57**	**0**	**20**
**AVERAGE**	**83.8**	**41.8**	**42.1**	**0.0**	**29.6**	**0.0**	**29.6**	**1.4**	**28.2**	**28.2**	**1.7**	**12.2**	**3.5**	**8.6**	**8.6**	**3.5**	**0.0**	**0.9**	**0.0**	**30.2**	**0.0**	**30.2**	**1.2**	**29.1**	**29.1**	**1.8**	**11.8**	**3.4**	**8.5**	**8.5**	**3.4**	**0.0**	**1.2**
SEM	**0.9**	**0.6**	**0.4**	**0.0**	**0.7**	**0.0**	**0.7**	**0.2**	**0.8**	**0.8**	**0.2**	**0.4**	**0.2**	**0.5**	**0.5**	**0.2**	**0.0**	**0.2**	**0.0**	**0.4**	**0.0**	**0.4**	**0.2**	**0.4**	**0.4**	**0.2**	**0.5**	**0.3**	**0.4**	**0.4**	**0.3**	**0.0**	**0.2**
Stat. sig. (inter-clone) ‡p<0.05, ‡‡p<0.005		**‡‡**																															
p-value (2-tailed students t-test)		7.00E-01		1.00E+00	4.36E-01	1.00E+00	4.36E-01	5.02E-01	3.40E-01	3.40E-01	8.23E-01	5.88E-01	6.22E-01	7.73E-01	7.73E-01	6.22E-01	1.00E+00	3.85E-01															

Wwc2-specific siRNA (10μM), 1in2 micro-injection, >64-cell (E4.5) stage, anti-GATA4 & anti-NANOG

#	Total cell number per embryo	Cells in injected clone	Cells in non-injected clone	INJECTED CLONE															NON INJECTED CLONE														
				Nuclear abnormality	OUTER CELLS							INNER CELLS							Nuclear abnormality	OUTER CELLS							INNER CELLS						
						GATA4+VE	GATA4-VE	NANOG+VE	NANOG-VE	GATA4 & NANOG -VE	Apoptotic ♣		GATA4+VE	GATA4-VE	NANOG+VE	NANOG-VE	GATA4 & NANOG -VE	Apoptotic ♣			GATA4+VE	GATA4-VE	NANOG+VE	NANOG-VE	GATA4 & NANOG -VE	Apoptotic ♣		GATA4+VE	GATA4-VE	NANOG+VE	NANOG-VE	GATA4 & NANOG -VE	Apoptotic ♣
**1**	74	26	48	2	20	0	20	3	17	17	1	6	2	4	2	4	2	3	0	32	0	32	1	31	31	1	16	7	9	9	7	0	0
**2**	63	22	41	1	17	0	17	0	17	17	2	5	1	4	2	3	2	4	0	29	0	29	0	29	29	1	12	4	8	8	4	0	1
**3**	68	21	47	3	17	0	17	1	16	16	2	4	1	3	2	2	1	3	0	34	0	34	2	32	32	2	13	2	11	11	2	0	1
**4**	61	21	40	2	19	0	19	0	19	19	1	2	2	0	0	2	0	2	0	25	0	25	0	25	25	2	15	3	12	12	3	0	1
**5**	58	20	38	1	16	0	16	0	16	16	0	4	2	2	0	4	2	4	0	29	0	29	1	28	28	1	9	2	7	7	2	0	1
**6**	63	22	41	2	20	0	20	0	20	20	2	2	0	2	1	1	1	3	0	30	0	30	2	28	28	1	11	5	6	6	5	0	2
**7**	61	22	39	1	18	0	18	0	18	18	0	4	1	3	1	3	2	2	0	26	0	26	0	26	26	1	13	4	9	9	4	0	1
**8**	77	28	49	0	26	0	26	1	25	25	1	2	0	2	0	2	2	4	0	33	0	33	1	32	32	2	16	5	11	11	5	0	1
**9**	69	28	41	2	22	0	22	2	20	20	2	6	3	3	2	4	1	3	0	28	0	28	2	26	26	1	13	4	9	9	4	0	1
**10**	69	25	44	3	19	0	19	3	16	16	1	6	3	3	2	4	1	2	0	31	0	31	1	30	30	1	13	3	10	10	3	0	0
**11**	68	23	45	0	18	0	18	2	16	16	2	5	2	3	2	3	1	1	0	29	0	29	2	27	27	2	16	5	11	11	5	0	3
**12**	65	23	42	2	20	0	20	1	19	19	0	3	3	0	0	3	0	5	0	28	0	28	1	27	27	3	14	4	10	10	4	0	0
**13**	65	22	43	2	16	0	16	0	16	16	0	6	3	3	2	4	1	2	0	30	0	30	0	30	30	2	13	6	7	7	6	0	1
**14**	67	28	39	4	23	0	23	2	21	21	1	5	2	3	2	3	1	3	0	27	0	27	1	26	26	2	12	3	9	9	3	0	0
**15**	65	23	42	2	20	0	20	1	19	19	1	3	2	1	0	3	1	4	0	26	0	26	0	26	26	0	16	2	14	14	2	0	2
**16**	66	26	40	3	22	0	22	1	21	21	2	4	3	1	1	3	0	2	0	28	0	28	1	27	27	1	12	4	8	8	4	0	0
**17**	73	27	46	3	23	0	23	2	21	21	2	4	2	2	2	2	0	5	0	32	0	32	2	30	30	1	14	5	9	9	5	0	0
**18**	63	21	42	1	18	0	18	1	17	17	1	3	1	2	1	2	1	3	0	29	0	29	0	29	29	1	13	4	9	9	4	0	1
**19**	64	24	40	1	20	0	20	2	18	18	2	4	2	2	2	2	0	4	0	27	0	27	1	26	26	3	13	2	11	11	2	0	1
**20**	62	19	43	3	18	0	18	2	16	16	1	1	0	1	1	0	0	2	0	30	0	30	0	30	30	1	13	6	7	7	6	0	0
**21**	67	28	39	2	23	0	23	2	21	21	2	5	4	1	1	4	0	3	0	27	0	27	2	25	25	1	12	3	9	9	3	0	0
**22**	68	27	41	1	22	0	22	0	22	22	2	5	3	2	1	4	1	4	0	29	0	29	0	29	29	1	12	4	8	8	4	0	1
**23**	71	24	47	3	20	0	20	1	19	19	2	4	2	2	2	2	0	2	0	34	0	34	1	33	33	2	13	2	11	11	2	0	2
**24**	60	20	40	2	18	0	18	2	16	16	1	2	1	1	0	2	1	3	0	25	0	25	0	25	25	2	15	3	12	12	3	0	1
**TOTAL**	**1587**	**570**	**1017**	**46**	**475**	**0**	**475**	**29**	**446**	**446**	**31**	**95**	**45**	**50**	**29**	**66**	**21**	**73**	**0**	**698**	**0**	**698**	**21**	**677**	**677**	**35**	**319**	**92**	**227**	**227**	**92**	**0**	**21**
**AVERAGE**	**66.1**	**23.8**	**42.4**	**1.9**	**19.8**	**0.0**	**19.8**	**1.2**	**18.6**	**18.6**	**1.3**	**4.0**	**1.9**	**2.1**	**1.2**	**2.8**	**0.9**	**3.0**	**0.0**	**29.1**	**0.0**	**29.1**	**0.9**	**28.2**	**28.2**	**1.5**	**13.3**	**3.8**	**9.5**	**9.5**	**3.8**	**0.0**	**0.9**
SEM	**0.9**	**0.6**	**0.6**	**0.2**	**0.5**	**0.0**	**0.5**	**0.2**	**0.5**	**0.5**	**0.2**	**0.3**	**0.2**	**0.2**	**0.2**	**0.2**	**0.2**	**0.2**	**0.0**	**0.5**	**0.0**	**0.5**	**0.2**	**0.5**	**0.5**	**0.1**	**0.4**	**0.3**	**0.4**	**0.4**	**0.3**	**0.0**	**0.2**
Stat. sig. (inter-clone) ‡p<0.05, ‡‡p<0.005		**‡‡**		**‡‡**	**‡‡**		**‡‡**		**‡‡**	**‡‡**		**‡‡**	**‡‡**	**‡‡**	**‡‡**	**‡‡**	**‡‡**	**‡‡**															
p-value (2-tailed students t-test)		1.50E-25		4.98E-12	1.77E-16	1.00E+00	1.77E-16	2.02E-01	6.29E-18	6.29E-18	4.37E-01	1.68E-24	2.09E-06	7.89E-21	8.97E-24	4.31E-03	6.06E-07	2.17E-10															
Stat. sig. (exp. vs. con embryo) *p<0.05, **p<0.005	**	**		**	**		**		**	**		**	**	**	**	*	**	**									*						
p-value (2-tailed students t-test)	9.70E-16	1.93E-22	7.08E-01	2.15E-09	3.49E-14	1.00E+00	3.49E-14	5.20E-01	4.06E-13	4.06E-13	1.07E-01	8.46E-19	1.34E-05	9.22E-17	1.12E-19	2.40E-02	2.00E-05	7.66E-09	1.00E+00	1.23E-01	1.00E+00	1.23E-01	2.93E-01	2.20E-01	2.20E-01	1.74E-01	1.51E-02	2.41E-01	9.11E-02	9.11E-02	2.41E-01	1.00E+00	2.61E-01

Supplementary Tables ST22

Control siRNA (10μM), 1in2 micro-injection, >64-cell (E4.5) stage, anti-GATA4 & anti-SOX2

#	Total cell number per embryo	Cells in injected clone	Cells in non-injected clone	INJECTED CLONE: Nuclear abnormality	INJECTED CLONE: OUTER CELLS							INJECTED CLONE: INNER CELLS							NON INJECTED CLONE: Nuclear abnormality	NON INJECTED CLONE: OUTER CELLS							NON INJECTED CLONE: INNER CELLS						
						GATA4+VE	GATA4-VE	SOX2+VE	SOX2-VE	GATA4 & SOX2 -VE	Apoptotic ♣		GATA4+VE	GATA4-VE	SOX2+VE	SOX2-VE	GATA4 & SOX2 -VE	Apoptotic ♣			GATA4+VE	GATA4-VE	SOX2+VE	SOX2-VE	GATA4 & SOX2 -VE	Apoptotic ♣		GATA4+VE	GATA4-VE	SOX2+VE	SOX2-VE	GATA4 & SOX2 -VE	Apoptotic ♣
**1**	84	40	44	0	26	0	26	0	26	26	1	14	3	11	11	3	0	0	0	32	0	32	0	32	32	2	12	4	8	8	4	0	1
**2**	80	40	40	0	30	0	30	0	30	30	2	10	2	8	8	2	0	0	0	28	0	28	0	28	28	1	12	2	10	10	2	0	1
**3**	79	39	40	0	29	0	29	0	29	29	1	10	1	9	9	1	0	1	0	27	0	27	0	27	27	0	13	4	9	9	4	0	2
**4**	89	44	45	0	31	0	31	0	31	31	2	13	3	10	10	3	0	2	0	35	0	35	0	35	35	2	10	2	8	8	2	0	1
**5**	80	41	39	0	32	0	32	0	32	32	1	9	1	8	8	1	0	1	0	27	0	27	0	27	27	1	12	3	9	9	3	0	3
**6**	82	42	40	0	26	0	26	0	26	26	3	16	4	12	12	4	0	0	0	31	0	31	0	31	31	2	9	4	5	5	4	0	1
**7**	85	42	43	0	28	0	28	0	28	28	2	14	2	12	12	2	0	1	0	30	0	30	0	30	30	1	13	5	8	8	5	0	1
**8**	83	43	40	0	30	0	30	0	30	30	1	13	4	9	9	4	0	2	0	29	0	29	0	29	29	0	11	3	8	8	3	0	0
**9**	84	41	43	0	30	0	30	0	30	30	1	11	4	7	7	4	0	0	0	28	0	28	0	28	28	3	15	3	12	12	3	0	2
**10**	82	41	41	0	30	0	30	0	30	30	2	11	3	8	8	3	0	2	0	30	0	30	0	30	30	1	11	3	8	8	3	0	0
**11**	81	40	41	0	27	0	27	0	27	27	2	13	4	9	9	4	0	1	0	32	0	32	0	32	32	2	9	2	7	7	2	0	1
**12**	83	41	42	0	28	0	28	0	28	28	0	13	5	8	8	5	0	1	0	32	0	32	0	32	32	1	10	2	8	8	2	0	0
**13**	92	46	46	0	30	0	30	0	30	30	1	16	4	12	12	4	0	0	0	32	0	32	0	32	32	3	14	4	10	10	4	0	3
**14**	96	47	49	0	33	0	33	0	33	33	0	14	5	9	9	5	0	1	0	36	0	36	0	36	36	0	13	6	7	7	6	0	1
**15**	91	46	45	0	35	0	35	0	35	35	1	11	3	8	8	3	0	0	0	33	0	33	0	33	33	1	12	2	10	10	2	0	0
**16**	87	42	45	0	29	0	29	0	29	29	2	13	3	10	10	3	0	2	0	35	0	35	0	35	35	1	10	2	8	8	2	0	2
**TOTAL**	**1358**	**675**	**683**	**0**	**474**	**0**	**474**	**0**	**474**	**474**	**22**	**201**	**51**	**150**	**150**	**51**	**0**	**14**	**0**	**497**	**0**	**497**	**0**	**497**	**497**	**21**	**186**	**51**	**135**	**135**	**51**	**0**	**19**
**AVERAGE**	**84.9**	**42.2**	**42.7**	**0.0**	**29.6**	**0.0**	**29.6**	**0.0**	**29.6**	**29.6**	**1.4**	**12.6**	**3.2**	**9.4**	**9.4**	**3.2**	**0.0**	**0.9**	**0.0**	**31.1**	**0.0**	**31.1**	**0.0**	**31.1**	**31.1**	**1.3**	**11.6**	**3.2**	**8.4**	**8.4**	**3.2**	**0.0**	**1.2**
SEM	**1.2**	**0.6**	**0.7**	**0.0**	**0.6**	**0.0**	**0.6**	**0.0**	**0.6**	**0.6**	**0.2**	**0.5**	**0.3**	**0.4**	**0.4**	**0.3**	**0.0**	**0.2**	**0.0**	**0.7**	**0.0**	**0.7**	**0.0**	**0.7**	**0.7**	**0.2**	**0.4**	**0.3**	**0.4**	**0.4**	**0.3**	**0.0**	**0.2**
Stat. sig. (inter-clone) ‡p<0.05, ‡‡p<0.005		‡‡																															
p-value (2-tailed students t-test)		5.91E-01		1.00E+00	1.34E-01	1.00E+00	1.34E-01	1.00E+00	1.34E-01	1.34E-01	8.42E-01	1.76E-01	1.00E+00	1.10E-01	1.10E-01	1.00E+00	1.00E+00	3.33E-01															

W*wc2*-specific siRNA (10μM), 1in2 micro-injection, >64-cell (E4.5) stage, anti-GATA4 & anti-SOX2

#	Total cell number per embryo	Cells in injected clone	Cells in non-injected clone	INJECTED CLONE: Nuclear abnormality	INJECTED CLONE: OUTER CELLS							INJECTED CLONE: INNER CELLS							NON INJECTED CLONE: Nuclear abnormality	NON INJECTED CLONE: OUTER CELLS							NON INJECTED CLONE: INNER CELLS						
						GATA4+VE	GATA4-VE	SOX2+VE	SOX2-VE	GATA4 & SOX2 -VE	Apoptotic ♣		GATA4+VE	GATA4-VE	SOX2+VE	SOX2-VE	GATA4 & SOX2 -VE	Apoptotic ♣			GATA4+VE	GATA4-VE	SOX2+VE	SOX2-VE	GATA4 & SOX2 -VE	Apoptotic ♣		GATA4+VE	GATA4-VE	SOX2+VE	SOX2-VE	GATA4 & SOX2 -VE	Apoptotic ♣
**1**	64	26	38	2	22	0	22	0	22	22	2	4	1	3	1	3	2	3	0	27	0	27	0	27	27	1	11	2	9	9	2	0	2
**2**	66	22	44	0	17	0	17	0	17	17	1	5	3	2	1	4	1	4	0	30	0	30	0	30	30	3	14	3	11	11	3	0	2
**3**	66	23	43	1	19	0	19	0	19	19	2	4	2	2	2	2	0	1	0	32	0	32	0	32	32	1	11	4	7	7	4	0	0
**4**	76	26	50	2	20	0	20	0	20	20	1	6	2	4	3	3	1	2	0	36	0	36	0	36	36	1	14	4	10	10	4	0	1
**5**	69	26	43	1	25	0	25	0	25	25	1	1	1	0	0	1	0	3	0	29	0	29	0	29	29	2	14	3	11	11	3	0	1
**6**	64	20	44	1	15	0	15	0	15	15	2	5	1	4	3	2	1	4	0	31	0	31	0	31	31	1	13	4	9	9	4	0	2
**7**	63	21	42	3	16	0	16	0	16	16	0	5	3	2	2	3	0	2	0	28	0	28	0	28	28	0	14	2	12	12	2	0	1
**8**	72	27	45	4	21	0	21	0	21	21	3	6	4	2	1	5	1	4	0	33	0	33	0	33	33	1	12	3	9	9	3	0	2
**9**	70	27	43	2	23	0	23	0	23	23	1	4	2	2	2	2	0	2	0	29	0	29	0	29	29	3	14	4	10	10	4	0	0
**10**	65	22	43	1	19	0	19	0	19	19	2	3	0	3	3	0	0	3	0	31	0	31	0	31	31	1	12	3	9	9	3	0	0
**11**	64	23	41	0	18	0	18	0	18	18	2	5	2	3	1	4	2	4	0	28	0	28	0	28	28	1	13	2	11	11	2	0	2
**12**	64	24	40	3	18	0	18	0	18	18	1	6	3	3	2	4	1	1	0	27	0	27	0	27	27	2	13	1	12	12	1	0	0
**13**	68	26	42	2	21	0	21	0	21	21	2	5	4	1	1	4	0	5	0	31	0	31	0	31	31	1	11	2	9	9	2	0	2
**14**	70	29	41	1	23	0	23	0	23	23	3	6	3	3	3	3	0	4	0	26	0	26	0	26	26	3	15	4	11	11	4	0	0
**15**	70	22	48	4	18	0	18	0	18	18	1	4	2	2	0	4	2	3	0	32	0	32	0	32	32	2	16	6	10	10	6	0	1
**16**	71	27	44	2	22	0	22	0	22	22	1	5	2	3	2	3	1	2	0	29	0	29	0	29	29	0	15	4	11	11	4	0	1
**17**	74	30	44	2	24	0	24	0	24	24	2	6	3	3	2	4	1	1	0	30	0	30	0	30	30	1	14	2	12	12	2	0	0
**18**	73	23	50	1	19	0	19	0	19	19	2	4	4	0	0	4	0	3	0	38	0	38	0	38	38	2	12	4	8	8	4	0	1
**19**	71	28	43	0	23	0	23	0	23	23	0	5	2	3	2	3	1	2	0	28	0	28	0	28	28	1	15	3	12	12	3	0	2
**20**	73	26	47	2	21	0	21	0	21	21	3	5	4	1	1	4	0	0	0	33	0	33	0	33	33	3	14	5	9	9	5	0	0
**TOTAL**	**1373**	**498**	**875**	**34**	**404**	**0**	**404**	**0**	**404**	**404**	**32**	**94**	**48**	**46**	**32**	**62**	**14**	**53**	**0**	**608**	**0**	**608**	**0**	**608**	**608**	**30**	**267**	**65**	**202**	**202**	**65**	**0**	**20**
**AVERAGE**	**68.7**	**24.9**	**43.8**	**1.7**	**20.2**	**0.0**	**20.2**	**0.0**	**20.2**	**20.2**	**1.6**	**4.7**	**2.4**	**2.3**	**1.6**	**3.1**	**0.7**	**2.7**	**0.0**	**30.4**	**0.0**	**30.4**	**0.0**	**30.4**	**30.4**	**1.5**	**13.4**	**3.3**	**10.1**	**10.1**	**3.3**	**0.0**	**1.0**
SEM	**0.9**	**0.6**	**0.7**	**0.3**	**0.6**	**0.0**	**0.6**	**0.0**	**0.6**	**0.6**	**0.2**	**0.3**	**0.3**	**0.3**	**0.2**	**0.3**	**0.2**	**0.3**	**0.0**	**0.7**	**0.0**	**0.7**	**0.0**	**0.7**	**0.7**	**0.2**	**0.3**	**0.3**	**0.3**	**0.3**	**0.3**	**0.0**	**0.2**
Stat. sig. (inter-clone) ‡p<0.05, ‡‡p<0.005		‡‡		‡‡	‡‡		‡‡		‡‡	‡‡		‡‡	‡	‡‡	‡‡		‡‡	‡‡															
p-value (2-tailed students t-test)		5.35E-22		1.28E-07	1.52E-13	1.00E+00	1.52E-13	1.00E+00	1.52E-13	1.52E-13	7.31E-01	5.05E-22	2.79E-02	5.28E-21	5.67E-23	6.97E-01	1.25E-04	3.23E-05															
Stat. sig. (exp. vs. con embryo) *p<0.05, **p<0.005	**	**		**	**		**		**	**		**		**	**		**	**									**		**	**			
p-value (2-tailed students t-test)	8.29E-13	3.96E-20	2.92E-01	1.69E-06	1.64E-12	1.00E+00	1.64E-12	1.00E+00	1.64E-12	1.64E-12	4.35E-01	6.86E-16	5.45E-02	7.03E-17	1.06E-18	8.31E-01	5.56E-04	3.67E-05	1.00E+00	5.08E-01	1.00E+00	5.08E-01	1.00E+00	5.08E-01	5.08E-01	5.59E-01	2.76E-03	8.79E-01	2.41E-03	2.41E-03	8.79E-01	1.00E+00	5.45E-01

Supplementary Tables ST23

Control siRNA (10µM), 1in2 micro-injection, 32-cell (E3.5) stage, anti-CDX2

#	Total cell number per embryo	Cells in injected clone	Cells in non-injected clone	INJECTED CLONE							NON INJECTED CLONE						
				Nuclear abnormality	OUTER CELLS			INNER CELLS			Nuclear abnormality	OUTER CELLS			INNER CELLS		
						CDX2+VE	CDX2-VE		CDX2+VE	CDX2-VE			CDX2+VE	CDX2-VE		CDX2+VE	CDX2-VE
**1**	33	16	17	0	9	9	0	7	0	7	0	9	9	0	8	0	8
**2**	31	16	15	0	11	11	0	5	0	5	0	9	9	0	6	0	6
**3**	31	15	16	0	8	8	0	7	0	7	0	8	8	0	8	0	8
**4**	39	20	19	0	11	11	0	9	0	9	0	12	12	0	7	0	7
**5**	32	15	17	0	9	9	0	6	0	6	0	8	8	0	9	0	9
**6**	39	22	17	0	14	14	0	8	0	8	0	12	12	0	5	0	5
**7**	33	17	16	0	12	12	0	5	0	5	0	9	9	0	7	0	7
**8**	41	21	20	0	12	12	0	9	0	9	0	11	11	0	9	0	9
**9**	32	16	16	0	10	10	0	6	0	6	0	9	9	0	7	0	7
**10**	32	16	16	0	8	8	0	8	0	8	0	8	8	0	8	0	8
**11**	36	18	18	0	10	10	0	8	0	8	0	11	11	0	7	0	7
**12**	42	22	20	0	13	13	0	9	0	9	0	12	12	0	8	0	8
**13**	44	24	20	0	16	16	0	8	0	8	0	14	14	0	6	0	6
**14**	35	18	17	0	11	11	0	7	0	7	0	9	9	0	8	0	8
**TOTAL**	**500**	**256**	**244**	**0**	**154**	**154**	**0**	**102**	**0**	**102**	**0**	**141**	**141**	**0**	**103**	**0**	**103**
**AVERAGE**	**35.7**	**18.3**	**17.4**	**0.0**	**11.0**	**11.0**	**0.0**	**7.3**	**0.0**	**7.3**	**0.0**	**10.1**	**10.1**	**0.0**	**7.4**	**0.0**	**7.4**
SEM	**1.0**	**0.7**	**0.4**	**0.0**	**0.5**	**0.5**	**0.0**	**0.4**	**0.0**	**0.4**	**0.0**	**0.4**	**0.4**	**0.0**	**0.3**	**0.0**	**0.3**
Stat. sig. (inter-clone) ‡p<0.05, ‡‡p<0.005																	
p-value (2-tailed students t-test)		3.57E-01		1.00E+00	2.53E-01	2.53E-01	1.00E+00	8.83E-01	1.00E+00	8.83E-01							

Wwc2-specific siRNA (10µM), 1in2 micro-injection, 32-cell (E3.5) stage, anti-CDX2

#	Total cell number per embryo	Cells in injected clone	Cells in non-injected clone	INJECTED CLONE							NON INJECTED CLONE						
				Nuclear abnormality	OUTER CELLS			INNER CELLS			Nuclear abnormality	OUTER CELLS			INNER CELLS		
						CDX2+VE	CDX2-VE		CDX2+VE	CDX2-VE			CDX2+VE	CDX2-VE		CDX2+VE	CDX2-VE
**1**	33	11	22	1	9	6	3	2	0	2	0	14	14	0	8	0	8
**2**	32	13	19	2	10	8	2	3	0	3	0	12	12	0	7	0	7
**3**	29	10	19	0	9	7	2	1	0	1	0	13	13	0	6	0	6
**4**	29	11	18	0	9	6	3	2	0	2	0	11	11	0	7	0	7
**5**	34	12	22	1	10	10	0	2	0	2	0	13	13	0	9	0	9
**6**	28	10	18	0	10	8	2	0	0	0	0	12	12	0	6	0	6
**7**	35	11	24	0	9	6	3	2	0	2	0	16	16	0	8	0	8
**8**	28	10	18	2	7	7	0	3	0	3	0	12	12	0	6	0	6
**9**	25	9	16	1	8	6	2	1	0	1	0	11	11	0	5	0	5
**10**	25	10	15	0	10	9	1	0	0	0	0	9	9	0	6	0	6
**11**	29	14	15	2	12	9	3	2	0	2	0	9	9	0	6	0	6
**12**	29	13	16	1	10	8	2	3	0	3	0	8	8	0	8	0	8
**13**	34	14	20	2	12	9	3	2	0	2	0	13	13	0	7	0	7
**14**	30	11	19	0	11	10	1	0	0	0	0	11	11	0	8	0	8
**15**	31	13	18	0	12	8	4	1	0	1	0	12	12	0	6	0	6
**16**	35	11	24	2	9	6	3	2	0	2	0	16	16	0	8	0	8
**17**	26	9	17	0	8	8	0	1	0	1	0	10	10	0	7	0	7
**18**	28	11	17	1	9	7	2	2	0	2	0	12	12	0	5	0	5
**TOTAL**	**540**	**203**	**337**	**15**	**174**	**138**	**36**	**29**	**0**	**29**	**0**	**214**	**214**	**0**	**123**	**0**	**123**
**AVERAGE**	**30.0**	**11.3**	**18.7**	**0.8**	**9.7**	**7.7**	**2.0**	**1.6**	**0.0**	**1.6**	**0.0**	**11.9**	**11.9**	**0.0**	**6.8**	**0.0**	**6.8**
SEM	**0.8**	**0.4**	**0.7**	**0.2**	**0.3**	**0.3**	**0.3**	**0.2**	**0.0**	**0.2**	**0.0**	**0.5**	**0.5**	**0.0**	**0.3**	**0.0**	**0.3**
Stat. sig. (inter-clone) ‡p<0.05, ‡‡p<0.005		**‡‡**		**‡‡**	**‡‡**	**‡‡**	**‡‡**	**‡‡**		**‡‡**							
p-value (2-tailed students t-test)		1.32E-11		2.27E-04	8.86E-04	4.66E-08	2.96E-08	2.86E-16	1.00E+00	2.86E-16							
Stat. sig. (exp. vs. con embryo) *p<0.05, **p<0.005	**	**		**	*	**	**	**		**		*	*				
p-value (2-tailed students t-test)	2.18E-04	1.32E-09	1.34E-01	1.06E-03	5.14E-02	1.64E-05	6.48E-07	2.30E-14	1.00E+00	2.30E-14	1.00E+00	1.89E-02	1.89E-02	1.00E+00	2.11E-01	1.00E+00	2.11E-01

Supplementary Tables ST24

Control siRNA (10μM), 1in2 micro-injection, 32-cell (E3.5) stage

#	Total cell number per embryo	Cells in injected clone	Cells in non-injected clone	INJECTED CLONE			NON INJECTED CLONE		
				Nuclear abnormality	OUTER CELLS	INNER CELLS	Nuclear abnormality	OUTER CELLS	INNER CELLS
**1**	38	19	19	0	12	7	0	12	7
**2**	31	14	17	0	8	6	0	8	9
**3**	35	17	18	0	11	6	0	12	6
**4**	37	19	18	0	12	7	0	12	6
**5**	31	14	17	0	8	6	0	8	9
**6**	32	15	17	0	10	5	0	10	7
**7**	32	18	14	0	11	7	0	9	5
**8**	36	19	17	0	11	8	0	11	6
**9**	31	15	16	0	9	6	0	9	7
**10**	34	16	18	0	10	6	0	10	8
**11**	37	19	18	0	11	8	0	11	7
**12**	31	13	18	0	10	3	0	12	6
**13**	39	20	19	0	13	7	0	12	7
**14**	35	17	18	0	11	6	0	11	7
**15**	35	17	18	0	11	6	0	12	6
**TOTAL**	**514**	**252**	**262**	**0**	**158**	**94**	**0**	**159**	**103**
**AVERAGE**	**34.3**	**16.8**	**17.5**	**0.0**	**10.5**	**6.3**	**0.0**	**10.6**	**6.9**
SEM	**0.7**	**0.6**	**0.3**	**0.0**	**0.4**	**0.3**	**0.0**	**0.4**	**0.3**
Stat. sig. (inter-clone) ‡p<0.05, ‡‡p<0.005									
p-value (2-tailed students t-test)		3.18E-01		1.00E+00	9.01E-01	1.73E-01			

Wwc2-specific siRNA (10μM), 1in2 micro-injection, 32-cell (E3.5) stage

#	Total cell number per embryo	Cells in injected clone	Cells in non-injected clone	INJECTED CLONE			NON INJECTED CLONE		
				Nuclear abnormality	OUTER CELLS	INNER CELLS	Nuclear abnormality	OUTER CELLS	INNER CELLS
**1**	38	16	22	2	12	4	0	15	7
**2**	33	13	20	1	11	2	0	12	8
**3**	28	11	17	0	9	2	0	11	6
**4**	33	14	19	3	11	3	0	12	7
**5**	38	16	22	0	12	4	0	13	9
**6**	29	11	18	1	11	0	0	11	7
**7**	28	13	15	0	10	3	0	10	5
**8**	28	11	17	1	8	3	0	11	6
**9**	29	11	18	0	10	1	0	10	8
**10**	25	9	16	2	9	0	0	11	5
**11**	32	14	18	1	12	2	0	11	7
**12**	32	13	19	1	10	3	0	13	6
**13**	30	11	19	0	10	1	0	12	7
**14**	40	16	24	2	12	4	0	16	8
**15**	31	13	18	0	12	1	0	12	6
**16**	39	17	22	1	14	3	0	14	8
**17**	31	12	19	0	12	0	0	12	7
**18**	34	13	21	1	9	4	0	14	7
**19**	28	10	18	1	9	1	0	12	6
**TOTAL**	**606**	**244**	**362**	**17**	**203**	**41**	**0**	**232**	**130**
**AVERAGE**	**31.9**	**12.8**	**19.1**	**0.9**	**10.7**	**2.2**	**0.0**	**12.2**	**6.8**
SEM	**1.0**	**0.5**	**0.5**	**0.2**	**0.4**	**0.3**	**0.0**	**0.4**	**0.2**
Stat. sig. (inter-clone) ‡p<0.05, ‡‡p<0.005		‡‡		‡‡	‡	‡‡			
p-value (2-tailed students t-test)		4.78E-10		7.81E-05	5.04E-03	1.41E-13			
Stat. sig. (exp. vs. con embryo) *p<0.05, **p<0.005		**	*	**		**		*	
p-value (2-tailed students t-test)	7.23E-02	1.32E-05	2.21E-02	4.07E-04	7.69E-01	3.84E-10	1.00E+00	5.58E-03	9.49E-01

Wwc2-specific siRNA (10μM) +HA-Wwc2 (siRNA resistant) mRNA (80ng/μL), 1in2 micro-injection, 32-cell (E3.5) stage

#	Total cell number per embryo	Cells in injected clone	Cells in non-injected clone	INJECTED CLONE			NON INJECTED CLONE		
				Nuclear abnormality	OUTER CELLS	INNER CELLS	Nuclear abnormality	OUTER CELLS	INNER CELLS
**1**	35	18	17	0	12	6	0	10	7
**2**	32	16	16	0	10	6	0	11	5
**3**	32	17	15	0	10	7	0	9	6
**4**	33	16	17	1	12	4	0	9	8
**5**	35	18	17	0	11	7	0	10	7
**6**	32	15	17	0	11	4	0	8	9
**7**	37	18	19	0	10	8	0	15	4
**8**	29	14	15	0	9	5	0	11	4
**9**	33	16	17	1	11	5	0	10	7
**10**	32	17	15	0	10	7	0	9	6
**11**	37	20	17	0	12	8	0	11	6
**12**	33	16	17	0	10	6	0	11	6
**13**	34	17	17	0	11	6	0	9	8
**14**	42	21	21	0	13	8	0	14	7
**15**	32	16	16	0	10	6	0	11	5
**16**	39	19	20	0	13	6	0	12	8
**17**	30	14	16	1	9	5	0	9	7
**18**	37	19	18	0	12	7	0	11	7
**19**	37	18	19	0	11	7	0	13	6
**20**	35	17	18	0	10	7	0	12	6
**21**	36	17	19	0	11	6	0	12	7
**22**	36	19	17	0	13	6	0	11	6
**TOTAL**	**758**	**378**	**380**	**3**	**241**	**137**	**0**	**238**	**142**
**AVERAGE**	**34.5**	**17.2**	**17.3**	**0.1**	**11.0**	**6.2**	**0.0**	**10.8**	**6.5**
SEM	**0.7**	**0.4**	**0.3**	**0.1**	**0.3**	**0.2**	**0.0**	**0.4**	**0.3**
Stat. sig. (inter-clone) ‡p<0.05, ‡‡p<0.005									
p-value (2-tailed students t-test)		8.59E-01		7.57E-02	7.64E-01	5.36E-01			
Stat. sig. (exp. vs. con embryo) *p<0.05, **p<0.005									
p-value (2-tailed students t-test)	8.50E-01	5.66E-01	6.93E-01	1.43E-01	3.38E-01	9.21E-01	1.00E+00	6.95E-01	3.16E-01
Stat. sig. (exp. vs. exp/KD embryo) §p<0.05, §§p<0.005	§	§§	§§	§§		§§		§	
p-value (2-tailed students t-test)	3.15E-02	3.05E-08	5.73E-03	5.97E-04	5.32E-01	1.86E-12	1.00E+00	1.18E-02	2.99E-01

Supplementary Tables ST25

Control siRNA (10µM), 1in2 micro-injection, >64-cell (E4.5) stage

#	Total cell number per embryo	Cells in injected clone	Cells in non-injected clone	INJECTED CLONE			NON INJECTED CLONE		
				Nuclear abnormality	OUTER CELLS	INNER CELLS	Nuclear abnormality	OUTER CELLS	INNER CELLS
**1**	74	36	38	0	26	10	0	29	9
**2**	72	34	38	0	25	9	0	28	10
**3**	76	39	37	0	29	10	0	28	9
**4**	79	39	40	0	28	11	0	30	10
**5**	73	36	37	0	27	9	0	29	8
**6**	80	39	41	0	29	10	0	32	9
**7**	73	36	37	0	28	8	0	28	9
**8**	79	40	39	0	28	12	0	29	10
**9**	78	40	38	0	31	9	0	26	12
**10**	82	42	40	0	31	11	0	30	10
**11**	78	38	40	0	28	10	0	28	12
**12**	81	41	40	0	30	11	0	31	9
**13**	74	37	37	0	29	8	0	30	7
**TOTAL**	**999**	**497**	**502**	**0**	**369**	**128**	**0**	**378**	**124**
**AVERAGE**	**76.8**	**38.2**	**38.6**	**0.0**	**28.4**	**9.8**	**0.0**	**29.1**	**9.5**
SEM	**0.9**	**0.6**	**0.4**	**0.0**	**0.5**	**0.3**	**0.0**	**0.4**	**0.4**
Stat. sig. (inter-clone) ‡p<0.05, ‡‡p<0.005									
p-value (2-tailed students t-test)		6.16E-01		1.00E+00	2.98E-01	5.54E-01			

Wwc2-specific siRNA (10µM), 1in2 micro-injection, >64-cell (E4.5) stage

#	Total cell number per embryo	Cells in injected clone	Cells in non-injected clone	INJECTED CLONE			NON INJECTED CLONE		
				Nuclear abnormality	OUTER CELLS	INNER CELLS	Nuclear abnormality	OUTER CELLS	INNER CELLS
**1**	54	18	36	2	17	1	0	27	9
**2**	64	23	41	1	21	2	0	30	11
**3**	73	26	47	2	24	2	0	35	12
**4**	57	19	38	1	19	0	0	28	10
**5**	57	21	36	1	20	1	0	27	9
**6**	68	27	41	3	25	2	0	30	11
**7**	66	25	41	0	24	1	0	31	10
**8**	54	18	36	2	18	0	0	28	8
**9**	61	21	40	0	19	2	0	29	11
**10**	60	23	37	2	22	1	0	27	10
**11**	72	29	43	4	26	3	0	36	7
**12**	64	21	43	1	21	0	0	33	10
**13**	68	25	43	2	23	2	0	31	12
**TOTAL**	**818**	**296**	**522**	**21**	**279**	**17**	**0**	**392**	**130**
**AVERAGE**	**62.9**	**22.8**	**40.2**	**1.6**	**21.5**	**1.3**	**0.0**	**30.2**	**10.0**
SEM	**1.8**	**1.0**	**0.9**	**0.3**	**0.8**	**0.3**	**0.0**	**0.8**	**0.4**
Stat. sig. (inter-clone) ‡p<0.05, ‡‡p<0.005		‡‡		‡‡	‡‡	‡‡			
p-value (2-tailed students t-test)		3.04E-12		2.53E-05	7.36E-08	2.17E-15			
Stat. sig. (exp. vs. con embryo) *p<0.05, **p<0.005	**	**		**	**	**			
p-value (2-tailed students t-test)	3.40E-07	1.43E-12	1.47E-01	2.53E-05	9.32E-08	1.81E-16	1.00E+00	2.61E-01	4.19E-01

Wwc2-specific siRNA (10µM) +HA-Wwc2 (siRNA resistant) mRNA (80ng/µL), 1in2 micro-injection, >64-cell (E4.5) stage

#	Total cell number per embryo	Cells in injected clone	Cells in non-injected clone	INJECTED CLONE			NON INJECTED CLONE		
				Nuclear abnormality	OUTER CELLS	INNER CELLS	Nuclear abnormality	OUTER CELLS	INNER CELLS
**1**	57	28	29	1	20	8	0	22	7
**2**	83	40	43	0	25	15	0	36	7
**3**	60	29	31	0	21	8	0	23	8
**4**	83	38	45	1	24	14	0	35	10
**5**	79	38	41	0	27	11	0	32	9
**6**	71	35	36	0	26	9	0	29	7
**7**	89	42	47	0	30	12	0	37	10
**8**	65	33	32	0	23	10	0	24	8
**9**	55	27	28	1	19	8	0	22	6
**10**	80	44	36	0	34	10	0	28	8
**11**	71	34	37	0	25	9	0	27	10
**12**	82	40	42	0	28	12	0	34	8
**13**	75	38	37	0	29	9	0	30	7
**14**	74	37	37	0	25	12	0	27	10
**15**	79	39	40	0	32	7	0	31	9
**16**	74	37	37	0	27	10	0	30	7
**17**	69	35	34	0	28	7	0	25	9
**18**	76	42	34	0	32	10	0	26	8
**TOTAL**	**1322**	**656**	**666**	**3**	**475**	**181**	**0**	**518**	**148**
**AVERAGE**	**73.4**	**36.4**	**37.0**	**0.2**	**26.4**	**10.1**	**0.0**	**28.8**	**8.2**
SEM	**2.2**	**1.1**	**1.2**	**0.1**	**1.0**	**0.5**	**0.0**	**1.1**	**0.3**
Stat. sig. (inter-clone) ‡p<0.05, ‡‡p<0.005						‡‡			
p-value (2-tailed students t-test)		7.44E-01		7.39E-02	1.17E-01	4.99E-03			
Stat. sig. (exp. vs. con embryo) *p<0.05, **p<0.005									*
p-value (2-tailed students t-test)	2.24E-01	2.28E-01	2.94E-01	1.30E-01	1.16E-01	7.64E-01	1.00E+00	8.29E-01	1.03E-02
Stat. sig. (exp. vs. exp/KD embryo) §p<0.05, §§p<0.005	§§	§§	§§	§§	§§	§§			§§
p-value (2-tailed students t-test)	1.59E-03	1.46E-09	7.01E-02	1.86E-05	9.12E-04	1.06E-13	1.00E+00	3.65E-01	1.14E-03

Supplementary Tables ST26

Control siRNA (10μM), 2in2 micro-injection, 32-cell (E3.5) stage

#	Total cell number per embryo	Total midbody number per embryo	Midbodies per total cell number	OUTER CELLS			INNER CELLS		
				Cell number	OUTER midbody	Midbodies per OUTER cell	Cell number	INNER midbody	Midbodies per INNER cell
**1**	33	12	0.36	21	9	0.43	12	3	0.25
**2**	38	7	0.18	24	7	0.29	14	0	0.00
**3**	39	10	0.26	26	8	0.31	13	2	0.15
**4**	43	17	0.40	28	11	0.39	15	6	0.40
**5**	33	14	0.42	23	10	0.43	10	4	0.40
**6**	31	12	0.39	22	9	0.41	9	3	0.33
**7**	41	13	0.32	26	8	0.31	15	5	0.33
**8**	35	13	0.37	24	9	0.38	11	4	0.36
**9**	37	12	0.32	25	7	0.28	12	5	0.42
**10**	33	10	0.30	23	8	0.35	10	2	0.20
**11**	40	8	0.20	26	7	0.27	14	1	0.07
**12**	31	6	0.19	22	6	0.27	9	0	0.00
**13**	34	11	0.32	23	8	0.35	11	3	0.27
**14**	33	14	0.42	23	10	0.43	10	4	0.40
**15**	37	12	0.32	24	9	0.38	13	3	0.23
**16**	36	8	0.22	25	6	0.24	11	2	0.18
**17**	35	10	0.29	24	10	0.42	11	0	0.00
**18**	33	7	0.21	23	6	0.26	10	1	0.10
**TOTAL**	**642**	**196**	**5.51**	**432**	**148**	**6.19**	**210**	**48**	**4.11**
**AVERAGE**	**35.7**	**10.9**	**0.31**	**24.0**	**8.2**	**0.34**	**11.7**	**2.7**	**0.23**
SEM	**0.8**	**0.7**	**0.02**	**0.4**	**0.4**	**0.02**	**0.5**	**0.4**	**0.03**

Wwc2-specific siRNA (10μM), 2in2 micro-injection, 32-cell (E3.5) stage

#	Total cell number per embryo	Total midbody number per embryo	Midbodies per total cell number	OUTER CELLS			INNER CELLS		
				Cell number	OUTER midbody	Midbodies per OUTER cell	Cell number	INNER midbody	Midbodies per INNER cell
**1**	16	2	0.13	14	2	0.14	2	0	0.00
**2**	16	5	0.31	15	4	0.27	1	1	1.00
**3**	13	4	0.31	12	4	0.33	1	0	0.00
**4**	24	5	0.21	19	5	0.26	5	0	0.00
**5**	19	6	0.32	17	6	0.35	2	0	0.00
**6**	18	7	0.39	16	7	0.44	2	0	0.00
**7**	25	5	0.20	20	4	0.20	5	1	0.20
**8**	18	5	0.28	15	5	0.33	3	0	0.00
**9**	9	3	0.33	9	3	0.33	0	0	0.00
**10**	15	5	0.33	14	5	0.36	1	0	0.00
**11**	16	5	0.31	13	5	0.38	3	0	0.00
**12**	15	4	0.27	14	4	0.29	1	0	0.00
**13**	14	4	0.29	12	4	0.33	2	0	0.00
**14**	11	2	0.18	11	2	0.18	0	0	0.00
**15**	17	3	0.18	16	3	0.19	1	0	0.00
**16**	16	4	0.25	15	3	0.20	1	1	1.00
**17**	14	2	0.14	13	2	0.15	1	0	0.00
**18**	14	2	0.14	13	2	0.15	1	0	0.00
**TOTAL**	**290**	**73**	**4.56**	**258**	**70**	**4.90**	**32**	**3**	**2.20**
**AVERAGE**	**16.1**	**4.1**	**0.25**	**14.3**	**3.9**	**0.27**	**1.8**	**0.2**	**0.12**
SEM	**0.9**	**0.3**	**0.02**	**0.6**	**0.3**	**0.02**	**0.3**	**0.1**	**0.08**
Stat. sig. (inter-clone) ‡$p<0.05$, ‡‡$p<0.005$	‡‡	‡‡		‡‡	‡‡	‡	‡‡	‡‡	
p-value (2-tailed students t-test)	2.68E-17	2.16E-10	5.29E-02	1.66E-14	3.09E-10	1.05E-02	1.75E-18	2.00E-06	2.14E-01
